# Supplementary material for: The Karnataka Individual Genome Project expands the human reference landscape to include South Asia
Source: HGG Adv. 2025 Sep 18;7(1):100516. doi: 10.1016/j.xhgg.2025.100516 (PMC12513208; doi:10.1016/j.xhgg.2025.100516)
Supplement: Document S1. Figures S1–S4, Table S1, and supplemental methods [file mmc1.pdf]

HGGA, Volume 7

Supplemental information

The Karnataka Individual Genome Project expands the human reference landscape to include South Asia

Apoorva Ganesh, Anisha Mhatre, Yash Chindarkar, Moushmi Goswami, Prakruti Mishra, Aditya Sharma, Manjushri Kalpande, Febina Ravindran, Subhashini Srinivasan, and Bibha Choudhary

Table of Contents

Supplementary Figure S1 (A-W): shows comparative dot plot of individuals' chromosomes from several genomes against virtual markers every 1Mb from T2T assembly. 13

Supplementary Figure S2 (A-E): shows comparative dot plot of individuals' chromosomes from several genomes against virtual markers every 100kb from hg38 assembly. 16

Supplementary Figure S3: Comparison of chromosome 3 and 5 of 69 accession of Arabidopsis (PMID: 38605175) compared to the virtual markers from the reference Arabidopsis genome. 17

Supplementary Figure S4: Comparison of PJL1.12x against PJL1.v1: Bench marking results for 12x coverage by down-sampled assembly from 30x raw reads of PJL1........ 29

Supplementary Table S1: 30

Supplementary Methods: 30

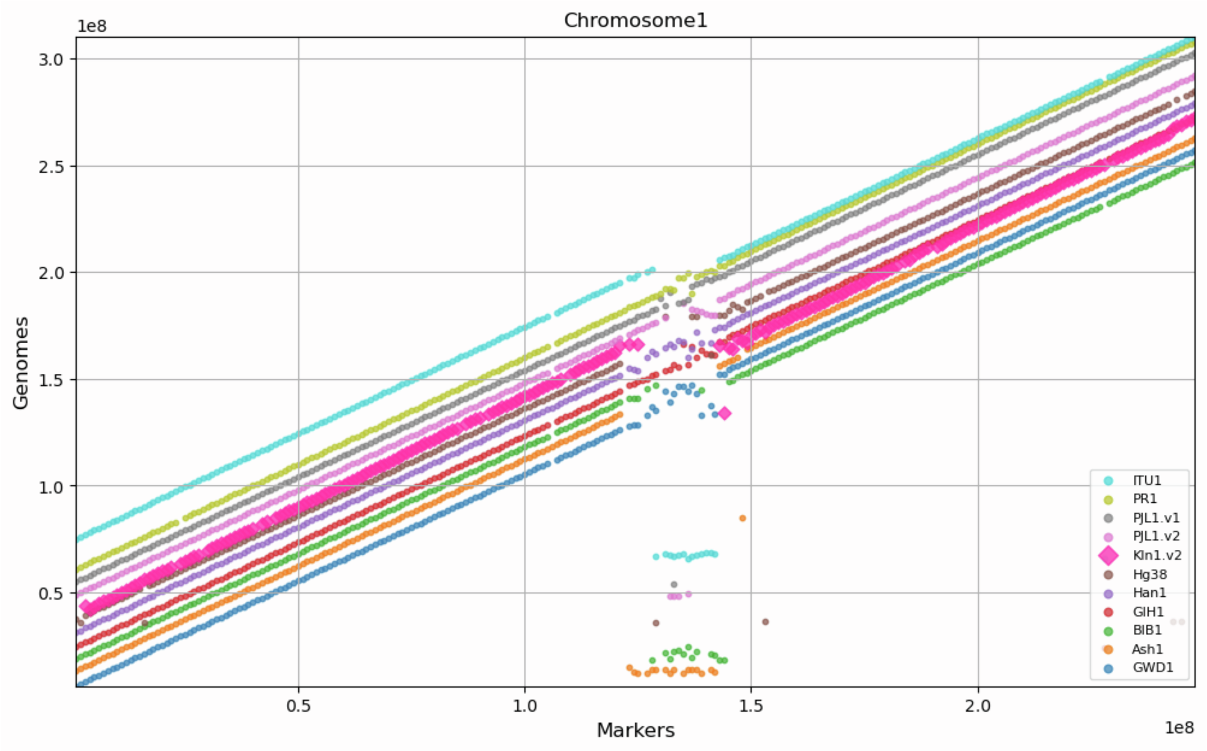
Chromosome1
1e8
3.0
2.5
2.0
1.5
1.0
0.5
Genomes
0.5
1.0
1.5
2.0
Markers
1e8
ITU1
PR1
PJL1.v1
PJL1.v2
Kln1.v2
Hg38
Han1
GIH1
BIB1
Ash1
GWD1

(A)

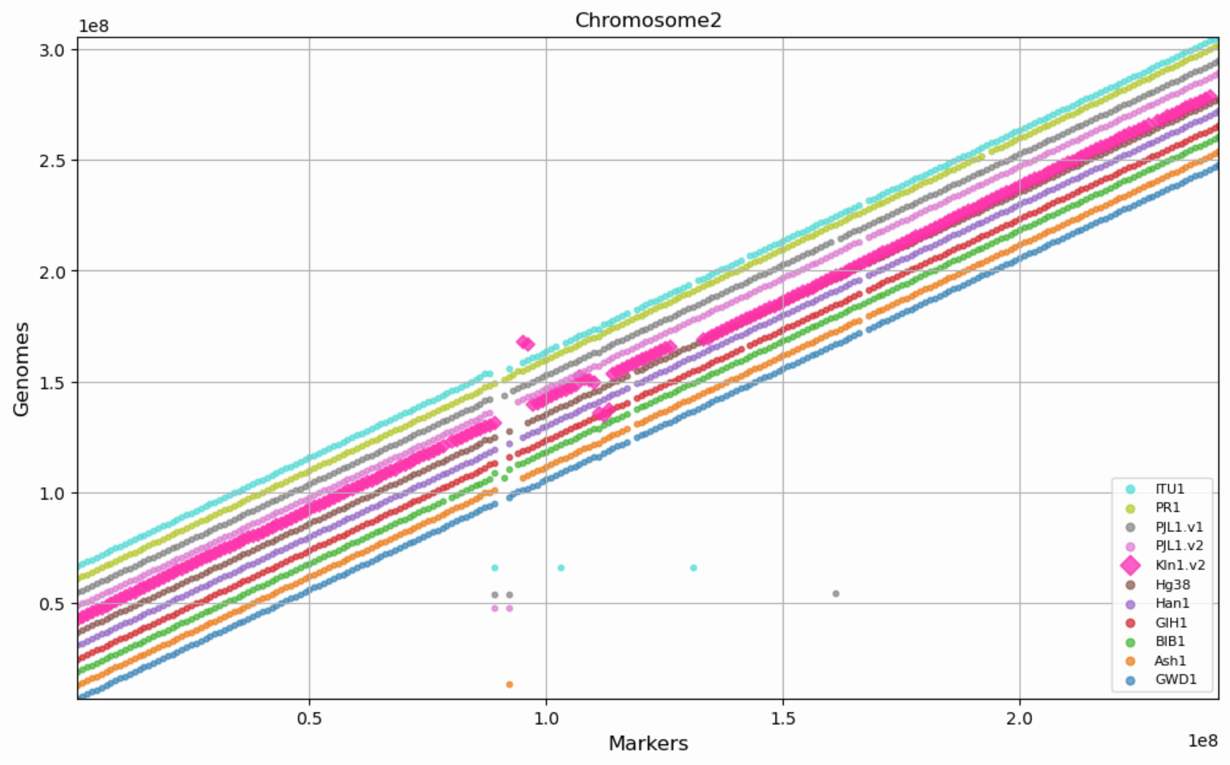
Chromosome2
1e8
3.0
2.5
2.0
1.5
1.0
0.5
Genomes
0.5
1.0
1.5
2.0
Markers
1e8
ITU1
PR1
PJL1.v1
PJL1.v2
Kln1.v2
Hg38
Han1
GIH1
BIB1
Ash1
GWD1

(B)

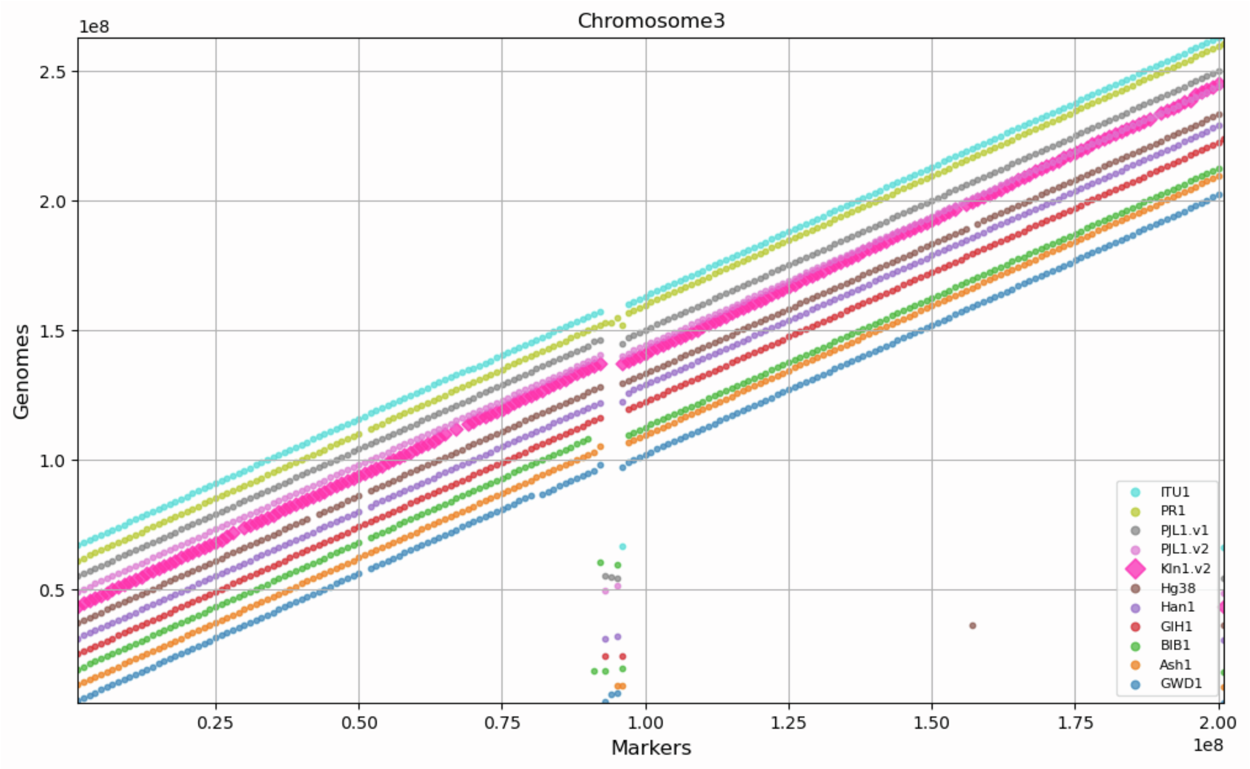
Chromosome3
1e8
2.5
2.0
1.5
1.0
0.5
Genomes
0.25
0.50
0.75
1.00
1.25
1.50
1.75
2.00
Markers
1e8
ITU1
PR1
PJL1.v1
PJL1.v2
Kln1.v2
Hg38
Han1
GIH1
BIB1
Ash1
GWD1

(C)

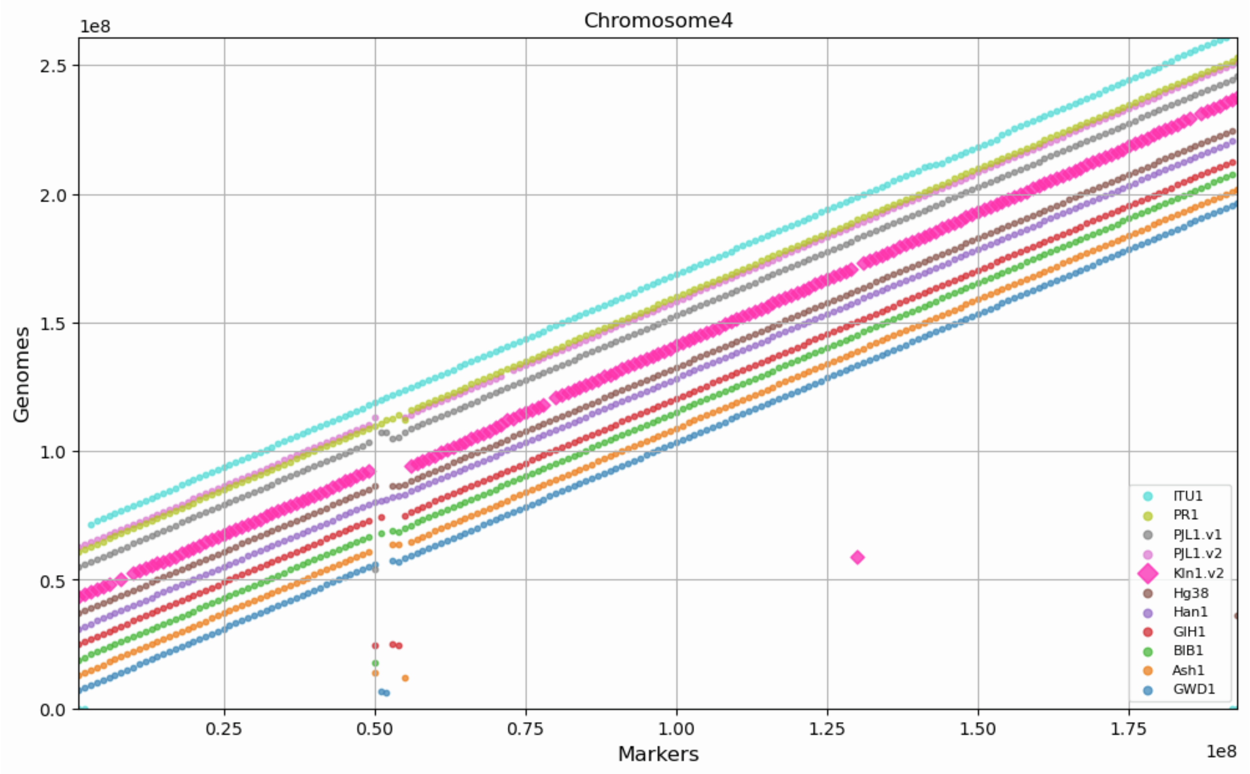
Chromosome4
1e8
2.5
2.0
1.5
1.0
0.5
0.0
Genomes
0.25
0.50
0.75
1.00
1.25
1.50
1.75
Markers
1e8
ITU1
PR1
PJL1.v1
PJL1.v2
Kln1.v2
Hg38
Han1
GIH1
BIB1
Ash1
GWD1

(D)

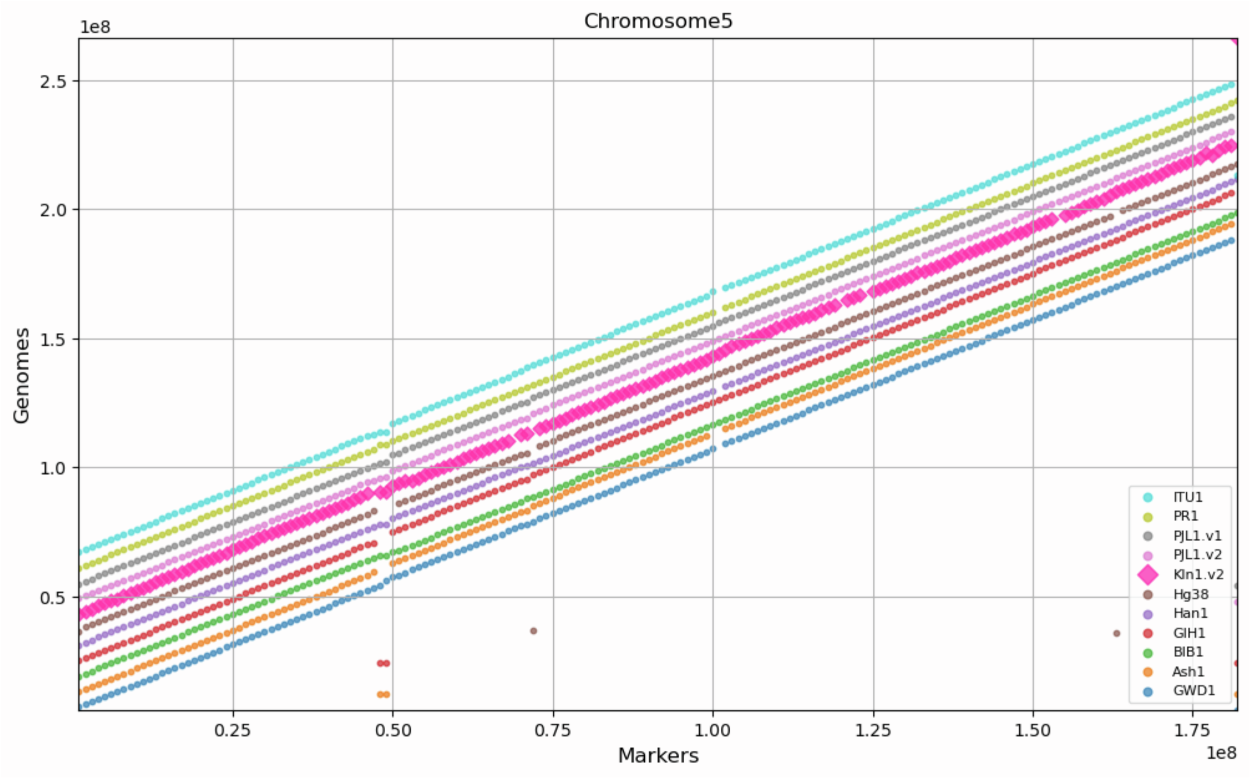
Chromosome5
1e8
2.5
2.0
1.5
1.0
0.5
Genomes
0.25
0.50
0.75
1.00
1.25
1.50
1.75
1e8
Markers
ITU1
PR1
PJL1.v1
PJL1.v2
Kln1.v2
Hg38
Han1
GIH1
BIB1
Ash1
GWD1

(E)

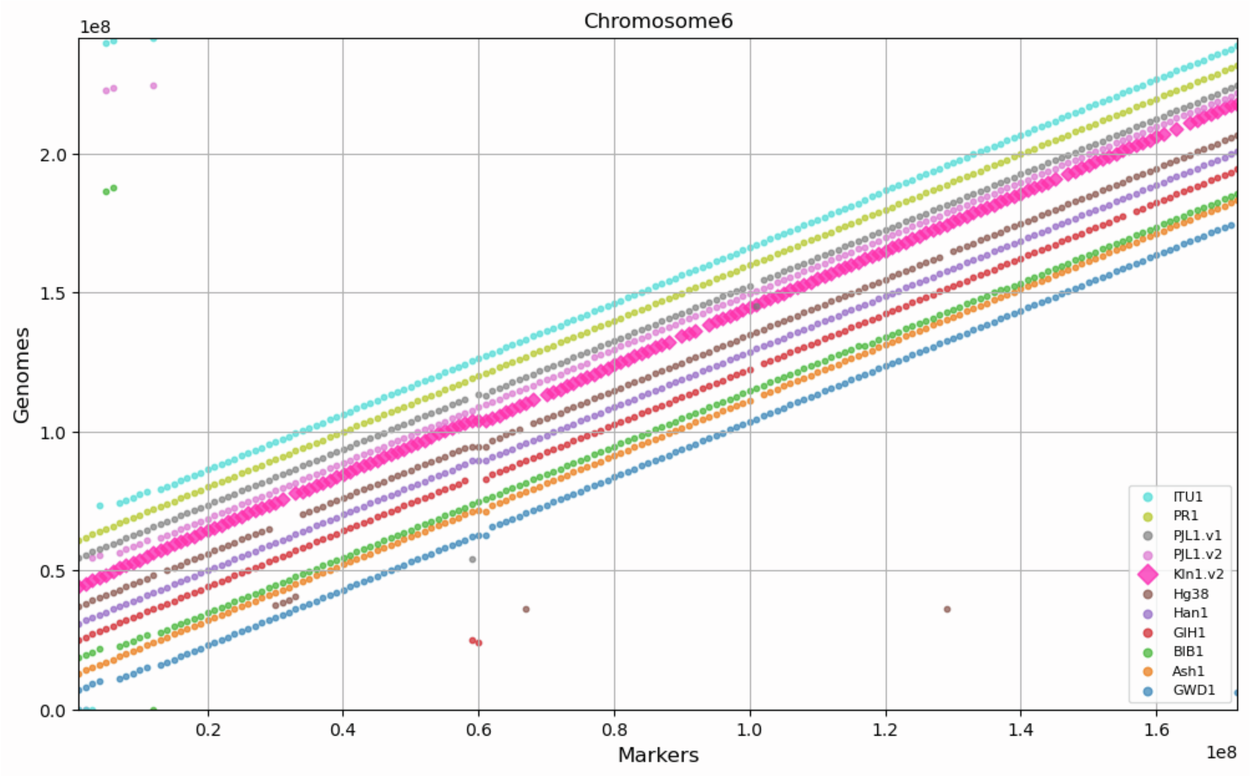
Chromosome6
1e8
2.0
1.5
1.0
0.5
0.0
Genomes
0.2
0.4
0.6
0.8
1.0
1.2
1.4
1.6
1e8
Markers
ITU1
PR1
PJL1.v1
PJL1.v2
Kln1.v2
Hg38
Han1
GIH1
BIB1
Ash1
GWD1

(F)

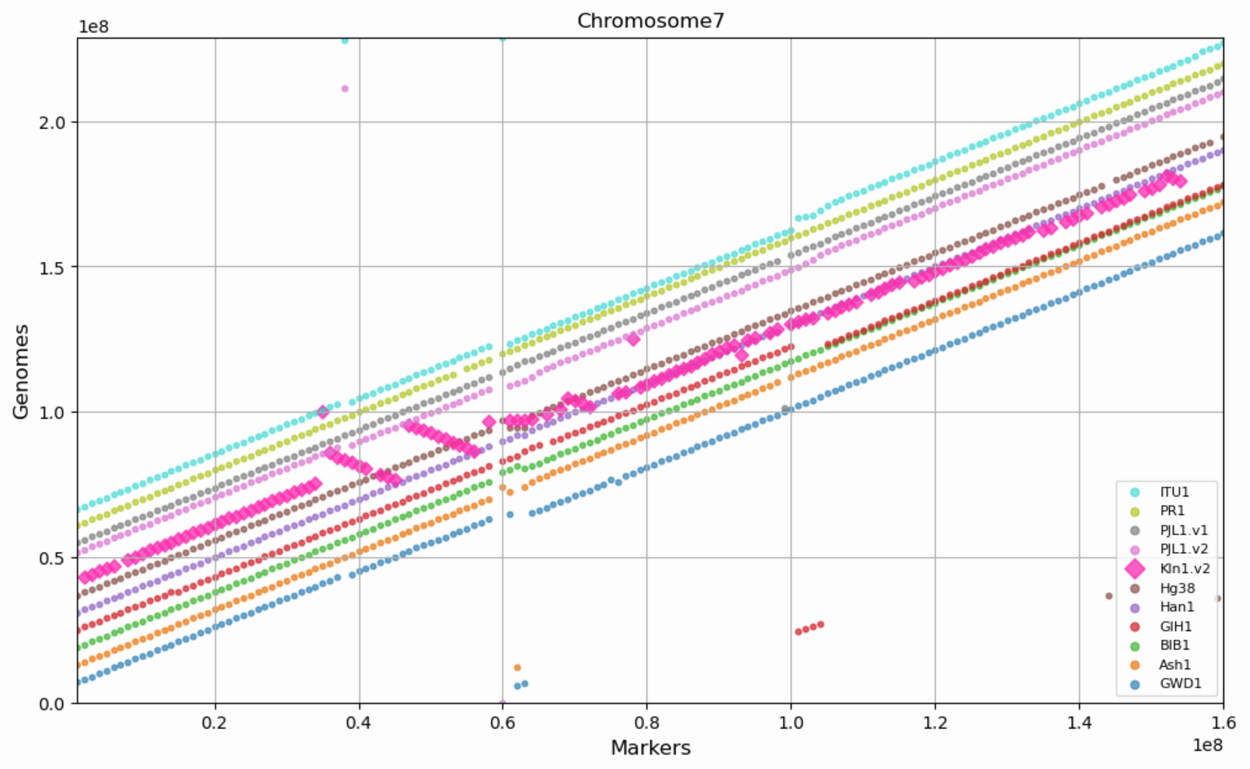
Chromosome7
1e8
2.0
1.5
1.0
0.5
0.0
Genomes
0.2
0.4
0.6
0.8
1.0
1.2
1.4
1.6
Markers
1e8
ITU1
PR1
PJL1.v1
PJL1.v2
Kln1.v2
Hg38
Han1
GIH1
BIB1
Ash1
GWD1

(G)

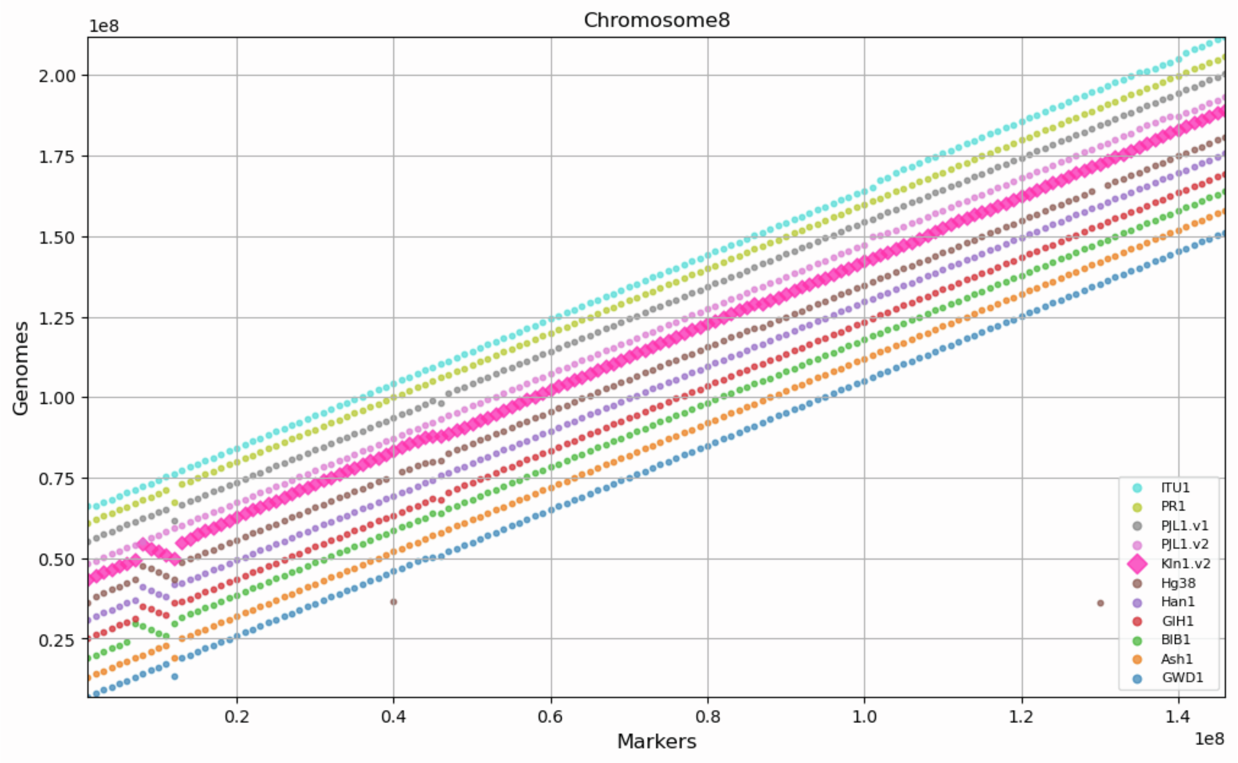
Chromosome8
1e8
2.00
1.75
1.50
1.25
1.00
0.75
0.50
0.25
Genomes
0.2
0.4
0.6
0.8
1.0
1.2
1.4
Markers
1e8
ITU1
PR1
PJL1.v1
PJL1.v2
Kln1.v2
Hg38
Han1
GIH1
BIB1
Ash1
GWD1

(H)

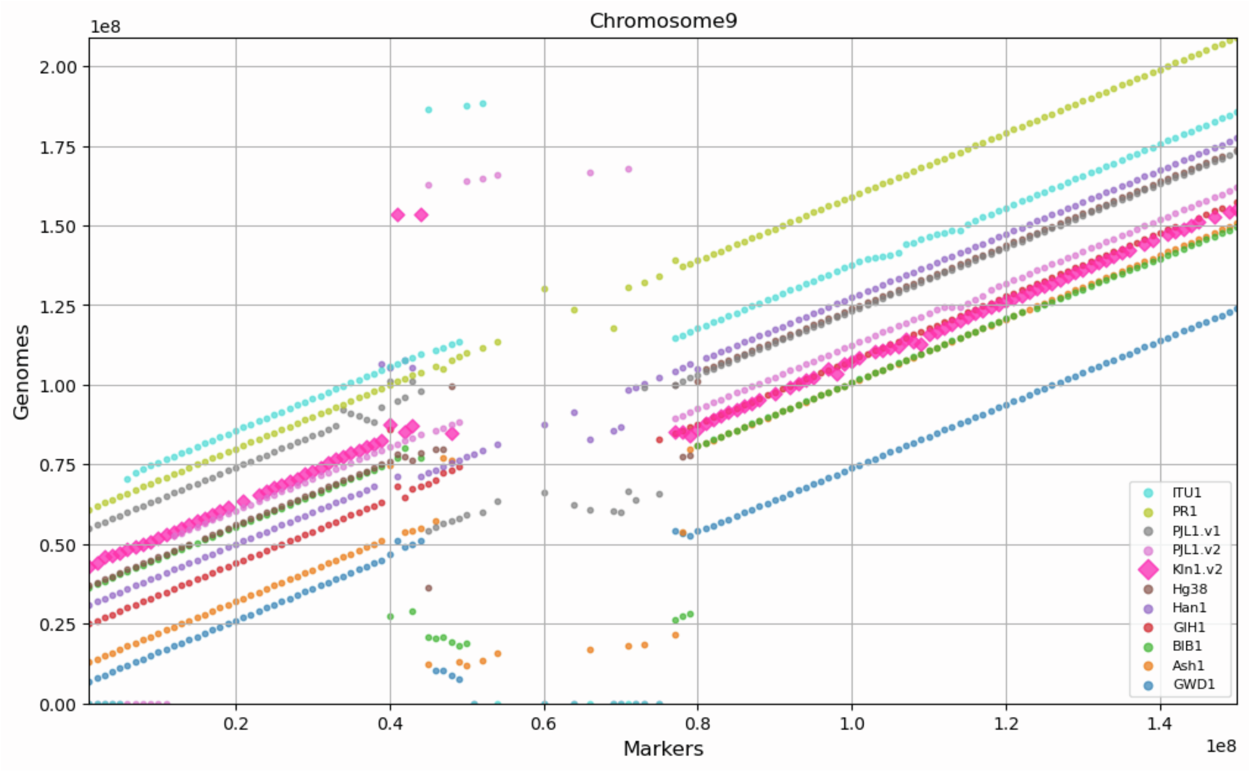
Chromosome9
1e8
2.00
1.75
1.50
1.25
1.00
0.75
0.50
0.25
0.00
Genomes
0.2
0.4
0.6
0.8
1.0
1.2
1.4
Markers
1e8
ITU1
PR1
PJL1.v1
PJL1.v2
Kln1.v2
Hg38
Han1
GIH1
BIB1
Ash1
GWD1

(I)

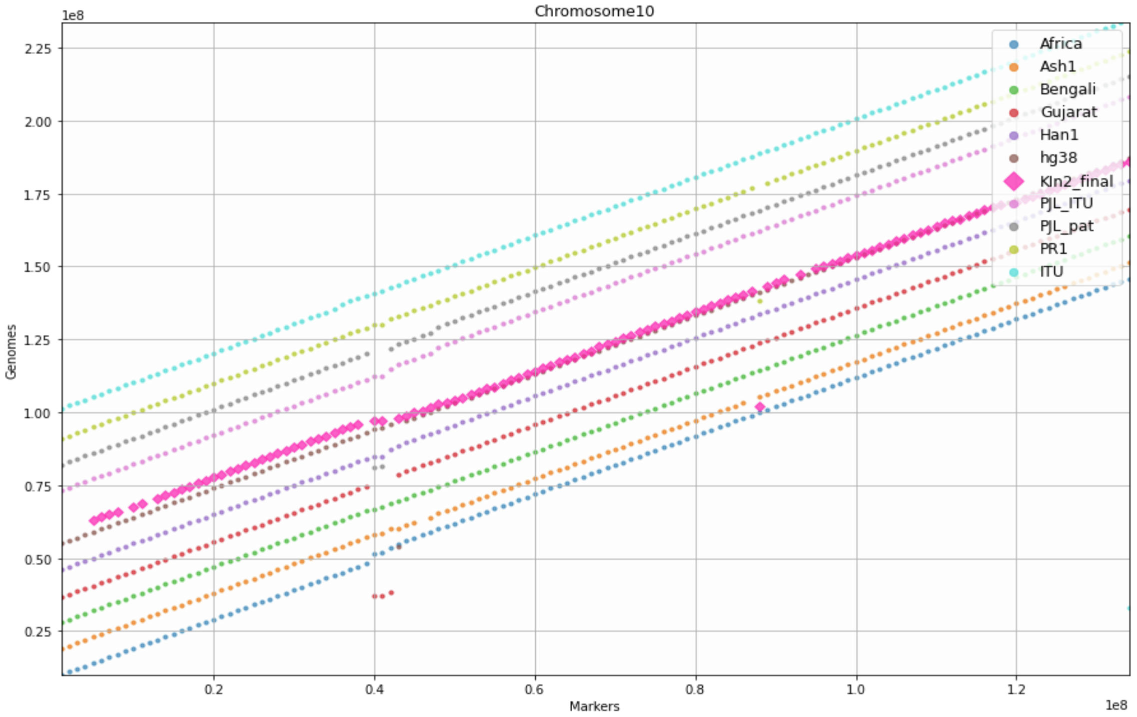
Chromosome10
1e8
2.25
2.00
1.75
1.50
1.25
1.00
0.75
0.50
0.25
Genomes
0.2
0.4
0.6
0.8
1.0
1.2
Markers
1e8
Africa
Ash1
Bengali
Gujarat
Han1
hg38
Kln2_final
PJL_ITU
PJL_pat
PR1
ITU

(J)

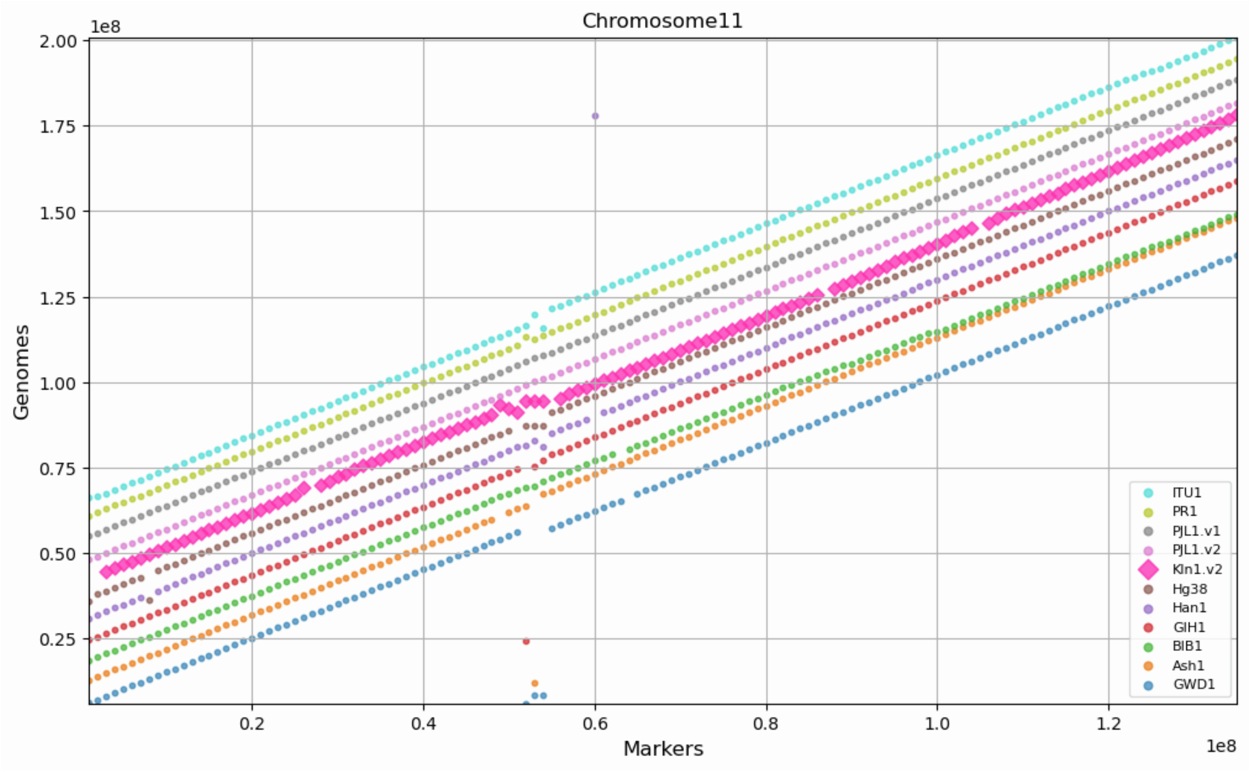
Chromosome11
1e8
2.00
1.75
1.50
1.25
1.00
0.75
0.50
0.25
Genomes
0.2
0.4
0.6
0.8
1.0
1.2
Markers
1e8
ITU1
PR1
PJL1.v1
PJL1.v2
Kln1.v2
Hg38
Han1
GIH1
BIB1
Ash1
GWD1

(K)

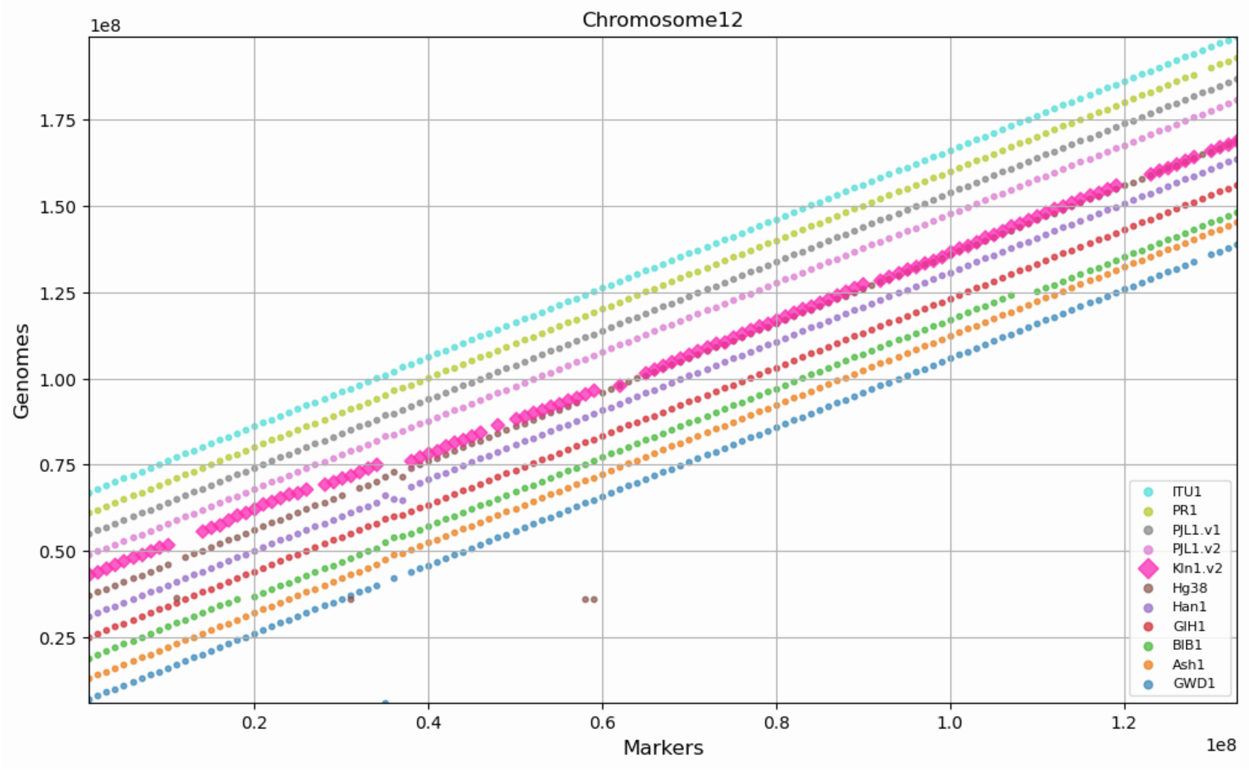
Chromosome12
1e8
1.75
1.50
1.25
1.00
0.75
0.50
0.25
Genomes
0.2
0.4
0.6
0.8
1.0
1.2
Markers
1e8
ITU1
PR1
PJL1.v1
PJL1.v2
Kln1.v2
Hg38
Han1
GIH1
BIB1
Ash1
GWD1

(L)

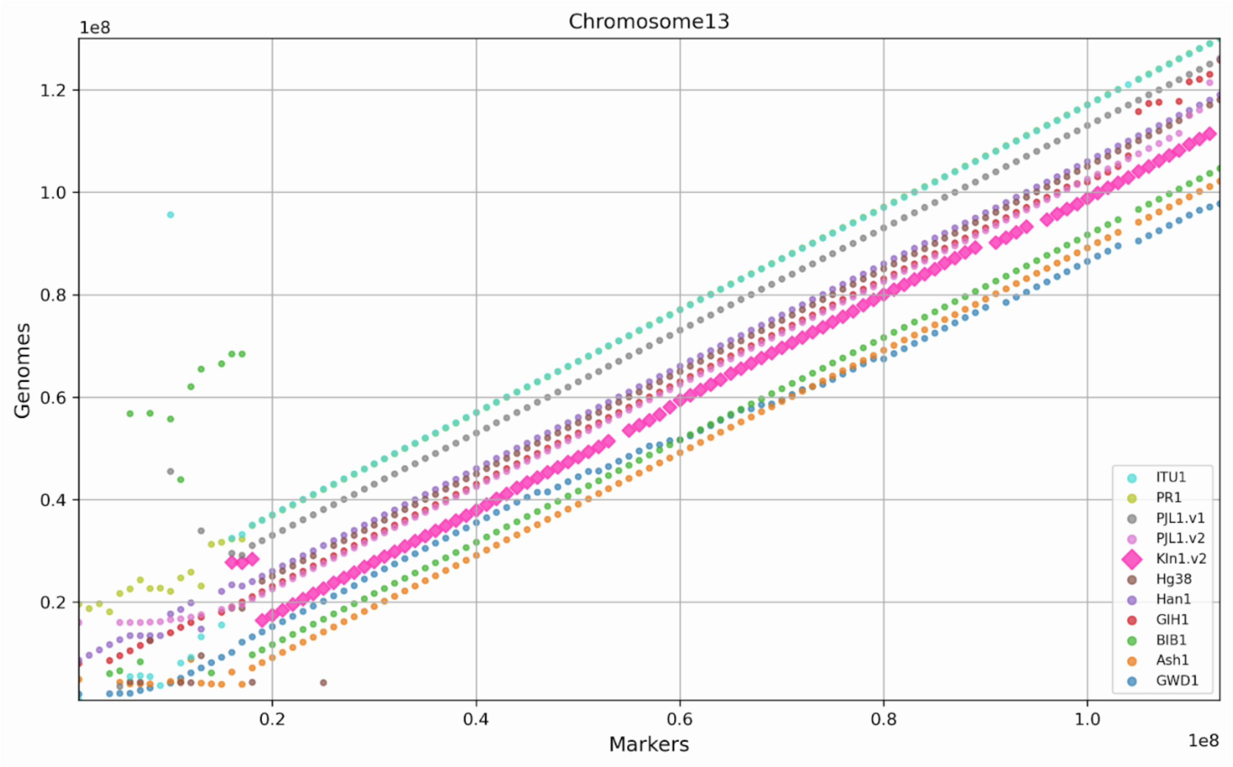
Chromosome13
1e8
1.2
1.0
0.8
0.6
0.4
0.2
Genomes
0.2
0.4
0.6
0.8
1.0
1e8
Markers
ITU1
PR1
PJL1.v1
PJL1.v2
Kln1.v2
Hg38
Han1
GIH1
BIB1
Ash1
GWD1

(M)

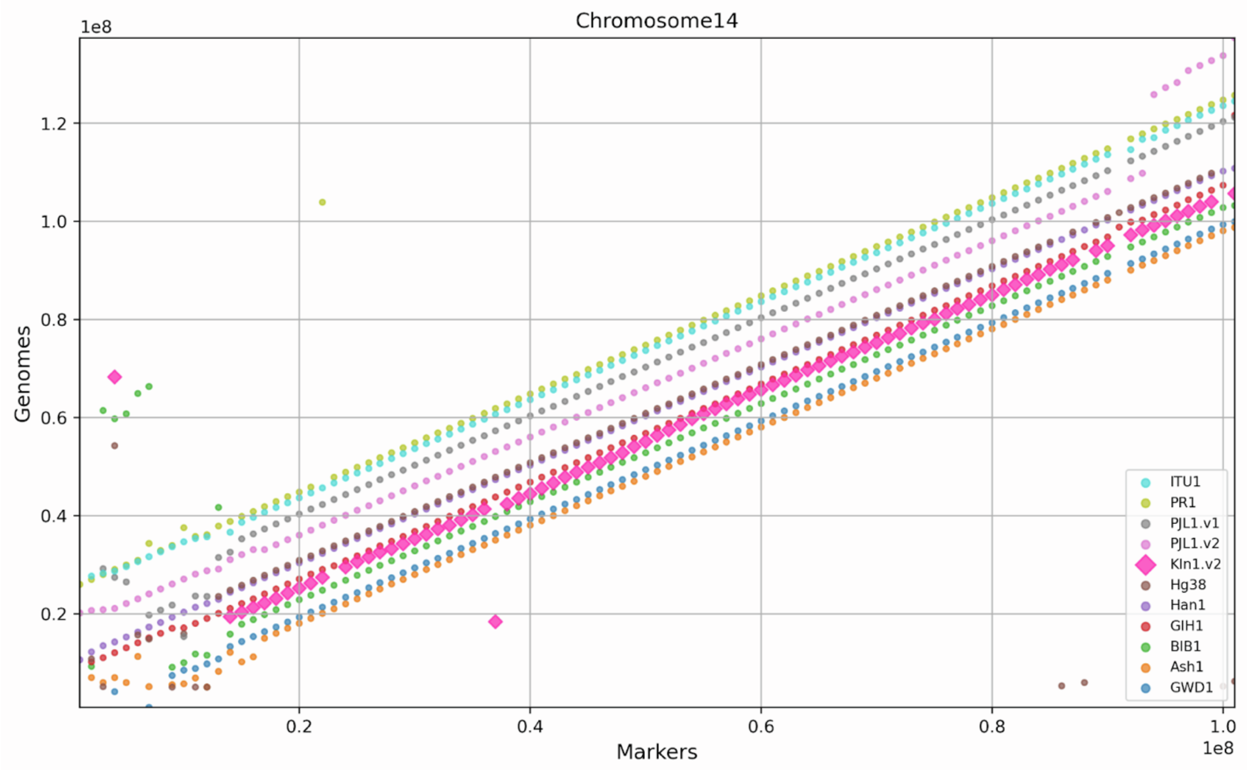
Chromosome14
1e8
1.2
1.0
0.8
0.6
0.4
0.2
Genomes
0.2
0.4
0.6
0.8
1.0
1e8
Markers
ITU1
PR1
PJL1.v1
PJL1.v2
Kln1.v2
Hg38
Han1
GIH1
BIB1
Ash1
GWD1

(N)

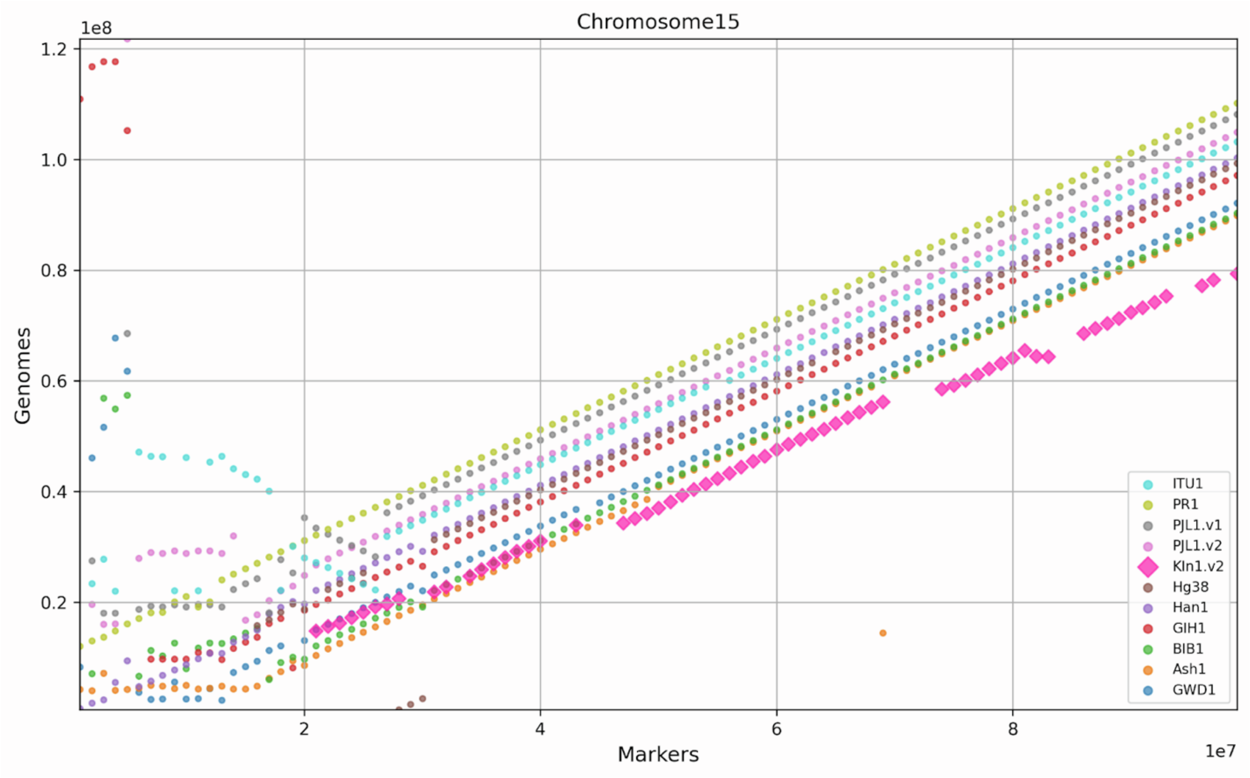

Chromosome15
1e8
1.2
1.0
0.8
0.6
0.4
0.2
Genomes
2
4
6
8
Markers
1e7
ITU1
PR1
PJL1.v1
PJL1.v2
KIn1.v2
Hg38
Han1
GIH1
BIB1
Ash1
GWD1

(O)

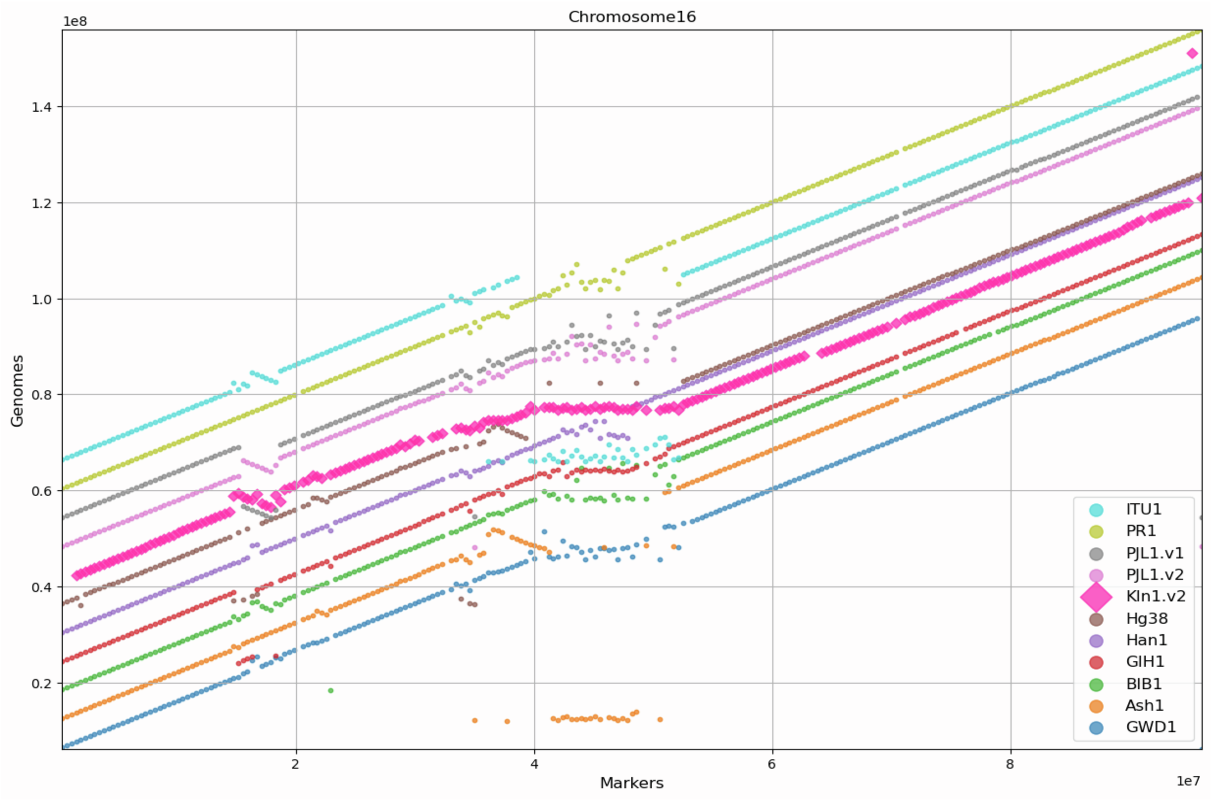

Chromosome16
1e8
1.4
1.2
1.0
0.8
0.6
0.4
0.2
Genomes
2
4
6
8
Markers
1e7
ITU1
PR1
PJL1.v1
PJL1.v2
KIn1.v2
Hg38
Han1
GIH1
BIB1
Ash1
GWD1

(P)

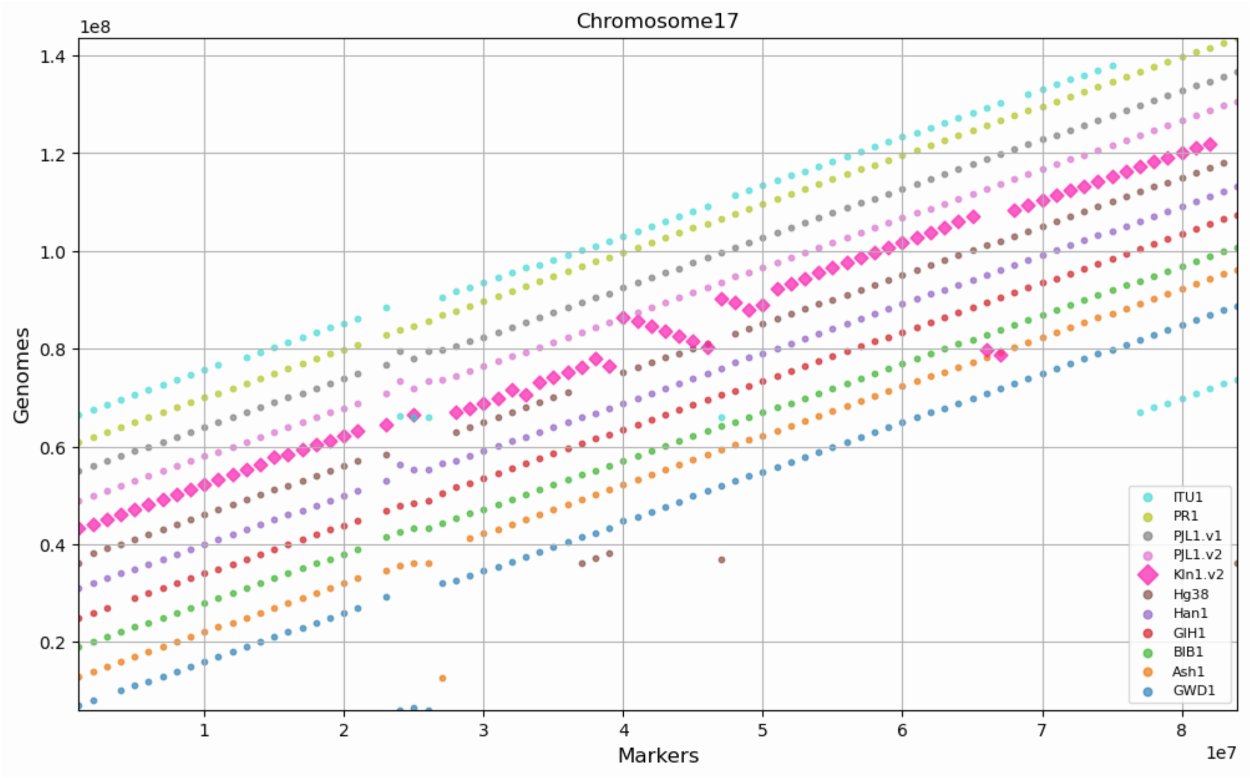
Chromosome17
1e8
1.4
1.2
1.0
0.8
0.6
0.4
0.2
Genomes
1
2
3
4
5
6
7
8
1e7
Markers
ITU1
PR1
PJL1.v1
PJL1.v2
Kln1.v2
Hg38
Han1
GIH1
BIB1
Ash1
GWD1

(Q)

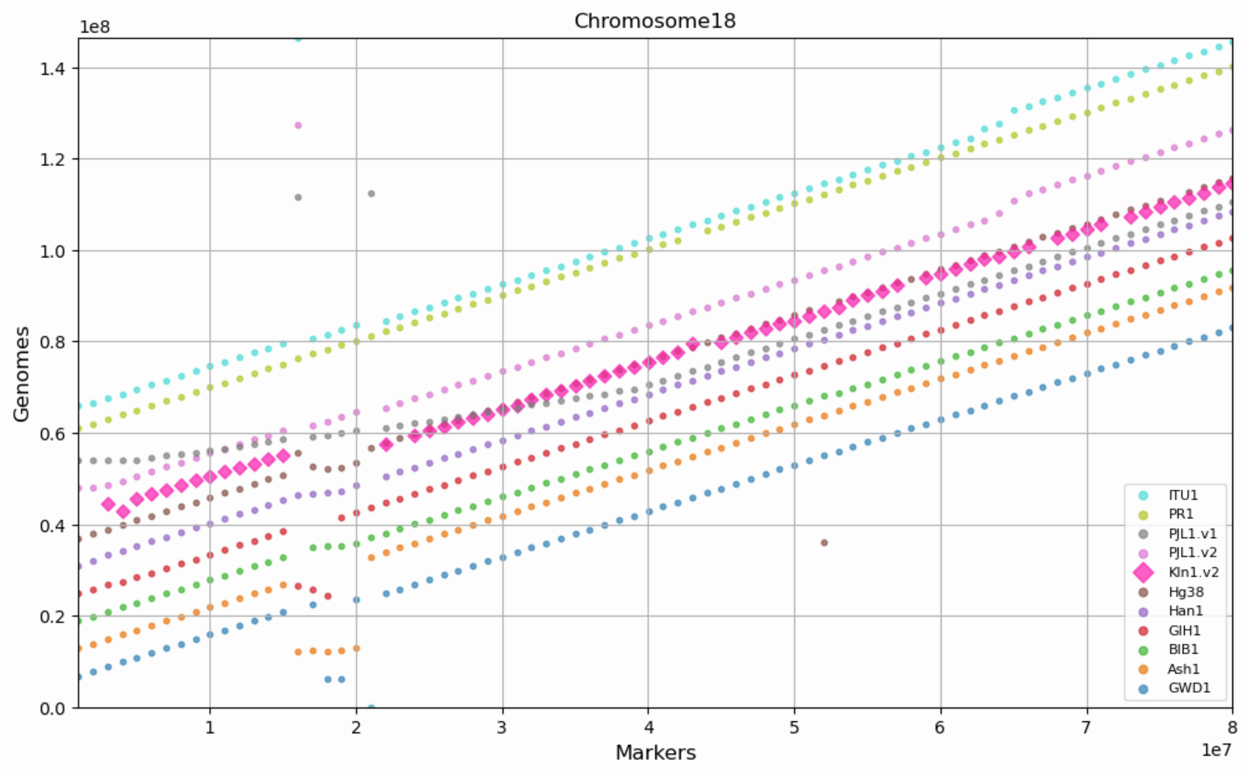
Chromosome18
1e8
1.4
1.2
1.0
0.8
0.6
0.4
0.2
0.0
Genomes
1
2
3
4
5
6
7
8
1e7
Markers
ITU1
PR1
PJL1.v1
PJL1.v2
Kln1.v2
Hg38
Han1
GIH1
BIB1
Ash1
GWD1

(R)

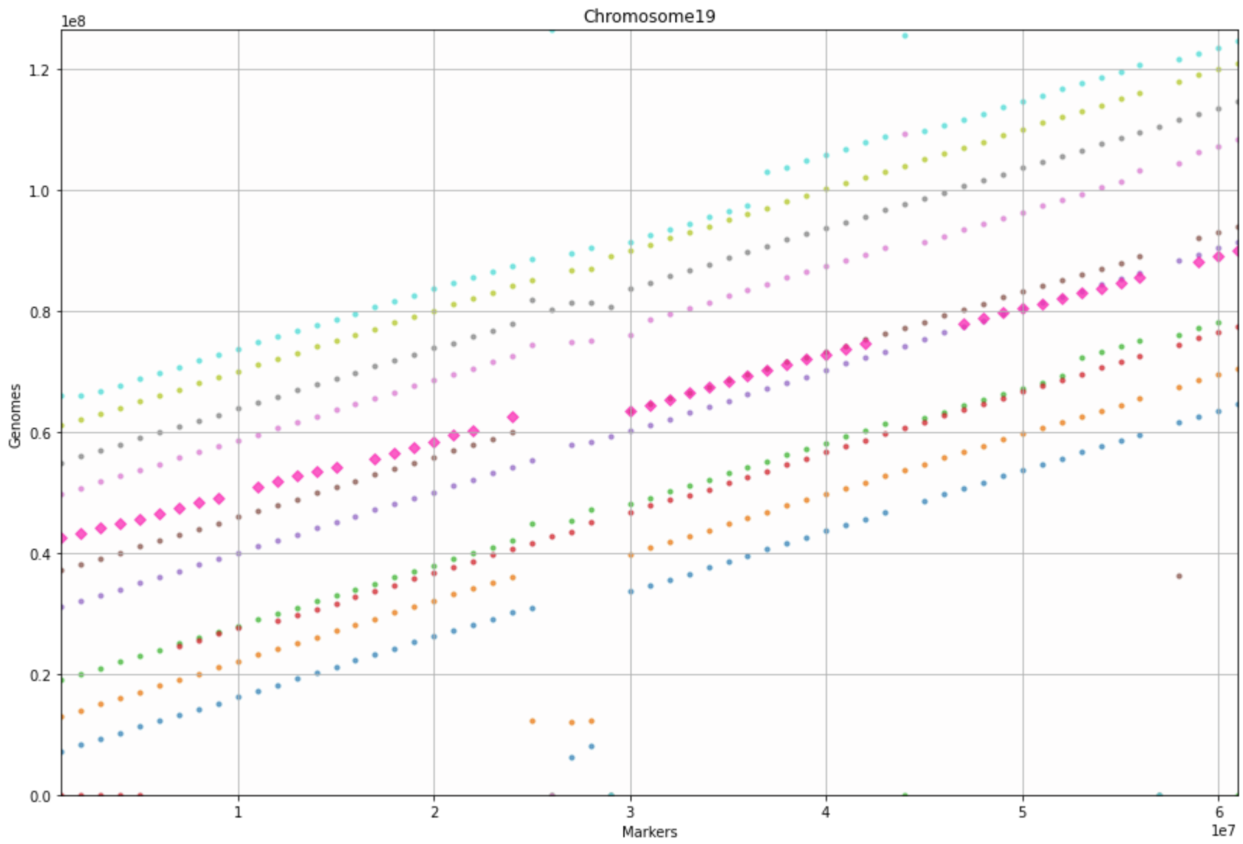
Chromosome19
1e8
1.2
1.0
0.8
0.6
0.4
0.2
0.0
Genomes
1
2
3
4
5
6
Markers
1e7

(S)

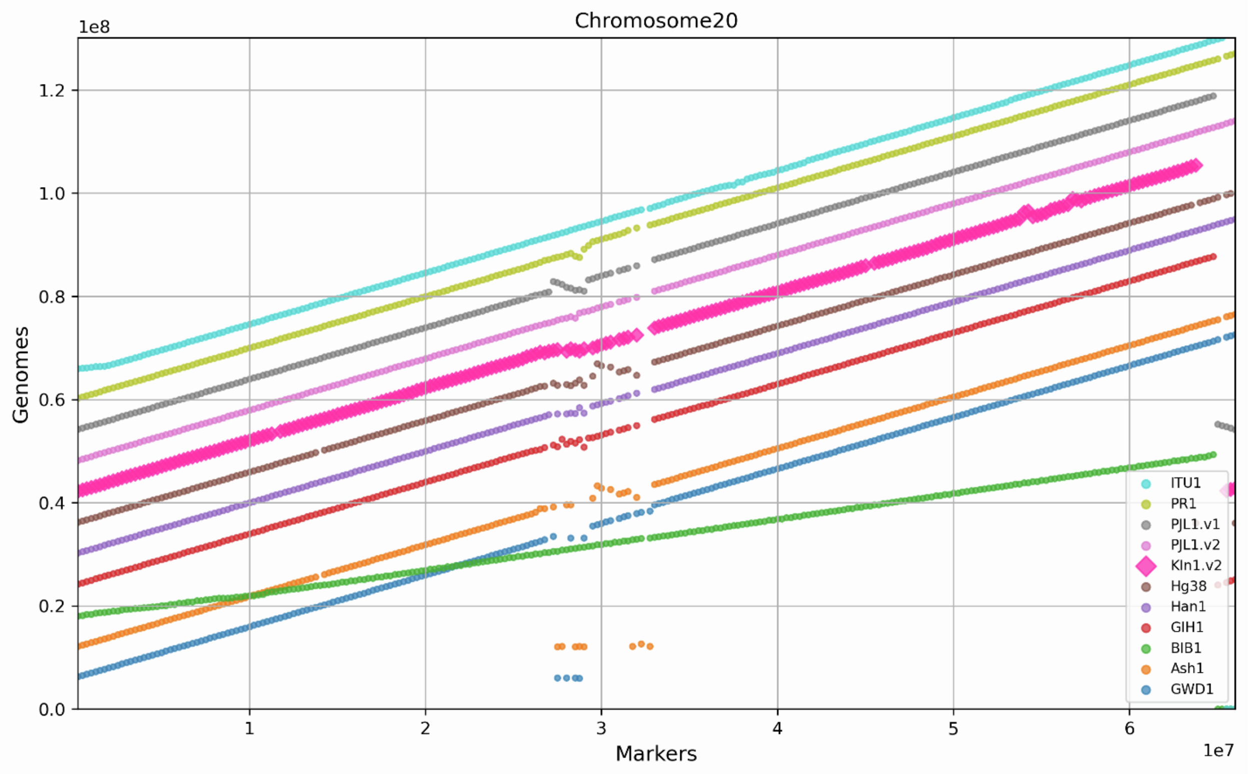
Chromosome20
1e8
1.2
1.0
0.8
0.6
0.4
0.2
0.0
Genomes
1
2
3
4
5
6
Markers
1e7
ITU1
PR1
PJL1.v1
PJL1.v2
Kln1.v2
Hg38
Han1
GIH1
BIB1
Ash1
GWD1

(T)

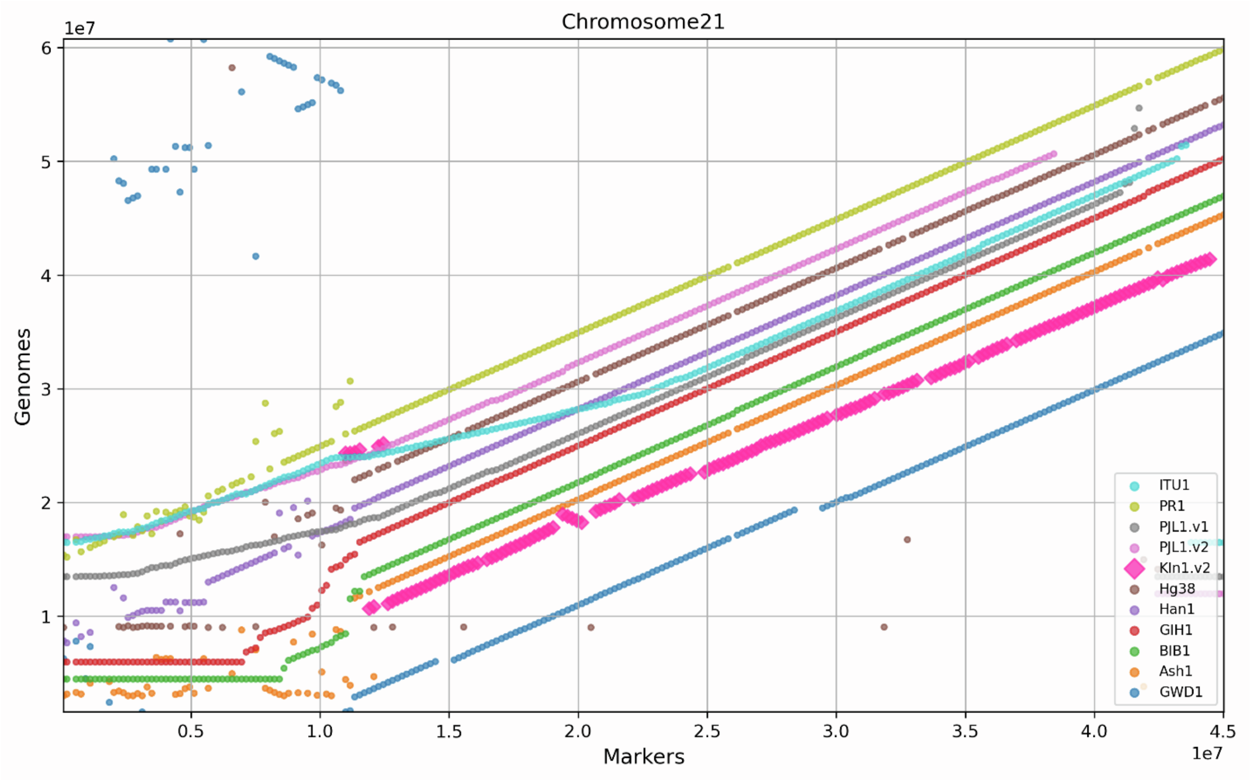
Chromosome21
1e7
6
5
4
3
2
1
Genomes
0.5
1.0
1.5
2.0
2.5
3.0
3.5
4.0
4.5
1e7
Markers
ITU1
PR1
PJL1.v1
PJL1.v2
Kln1.v2
Hg38
Han1
GIH1
BIB1
Ash1
GWD1

(U)

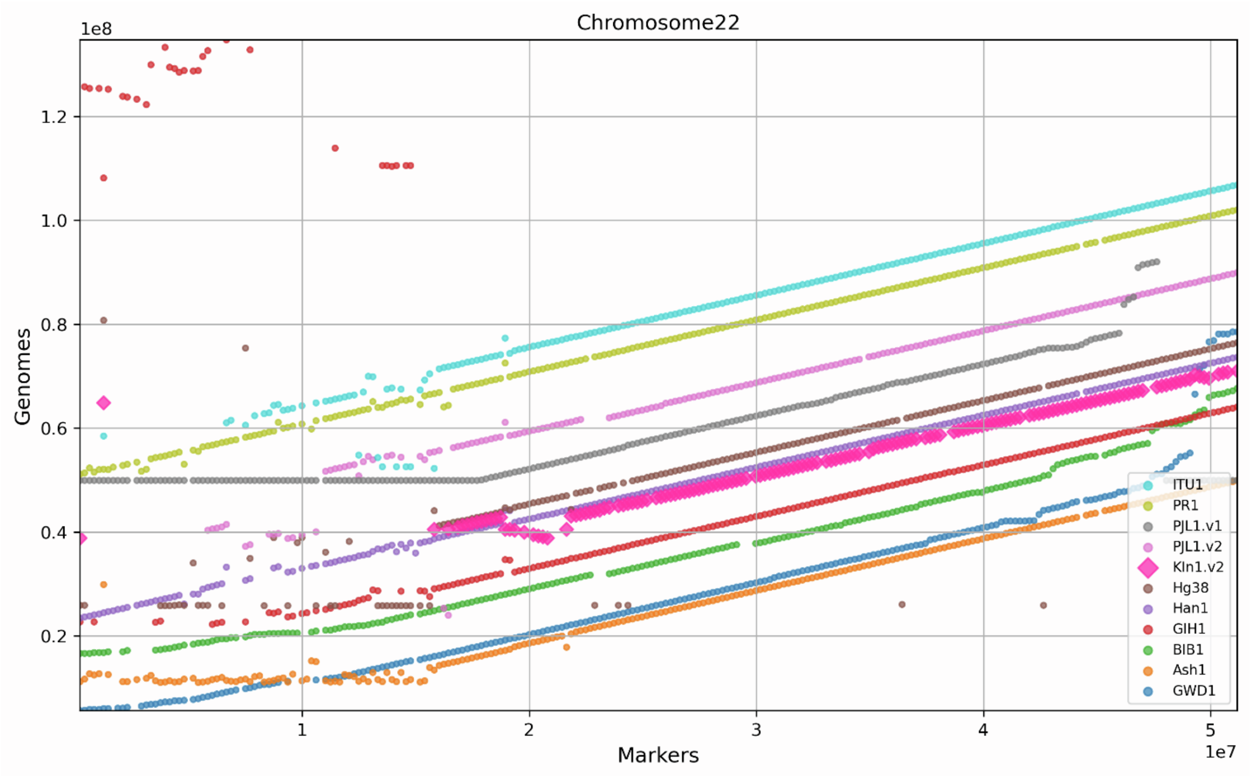
Chromosome22
1e8
1.2
1.0
0.8
0.6
0.4
0.2
Genomes
1
2
3
4
5
1e7
Markers
ITU1
PR1
PJL1.v1
PJL1.v2
Kln1.v2
Hg38
Han1
GIH1
BIB1
Ash1
GWD1

(V)

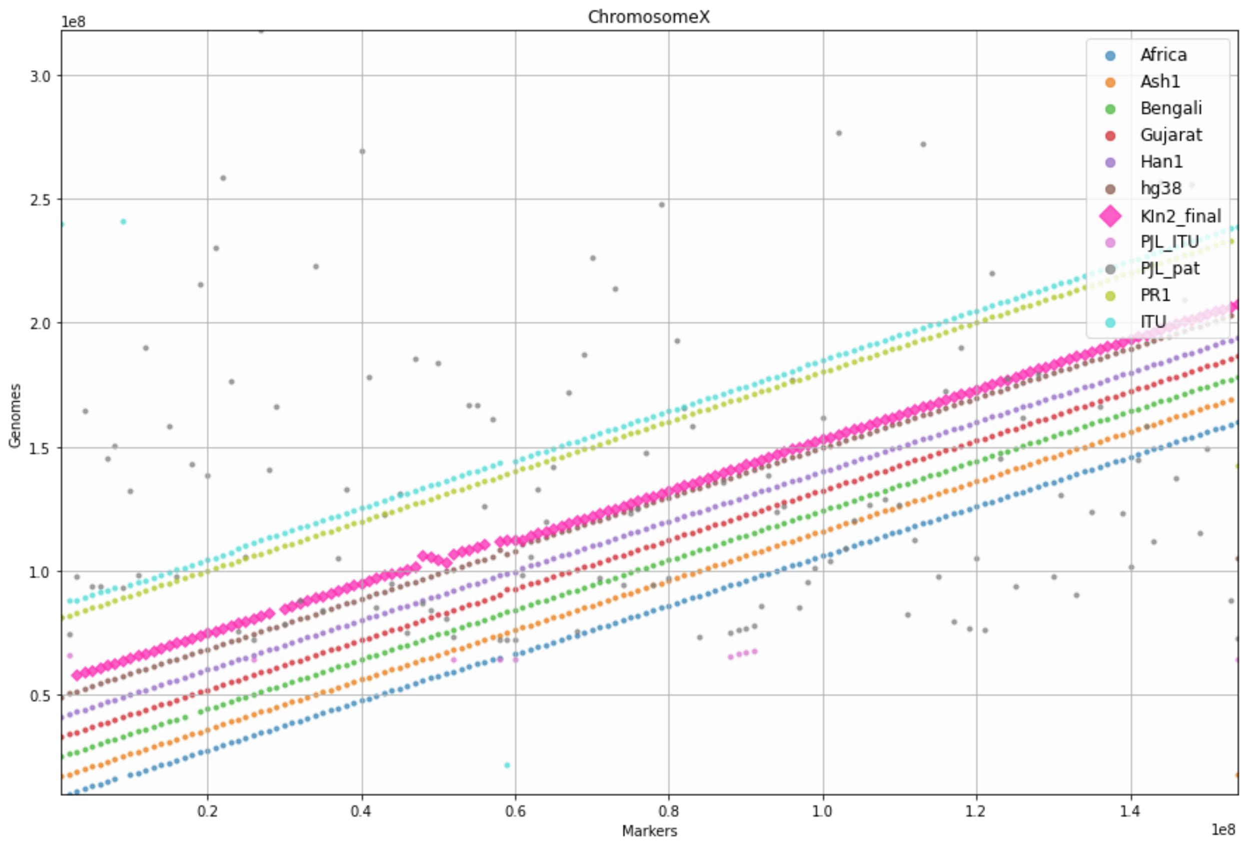

(W)

Supplementary Figure S1 (A-W): shows comparative dot plot of individuals' chromosomes from several genomes against virtual markers every 1Mb from T2T assembly.

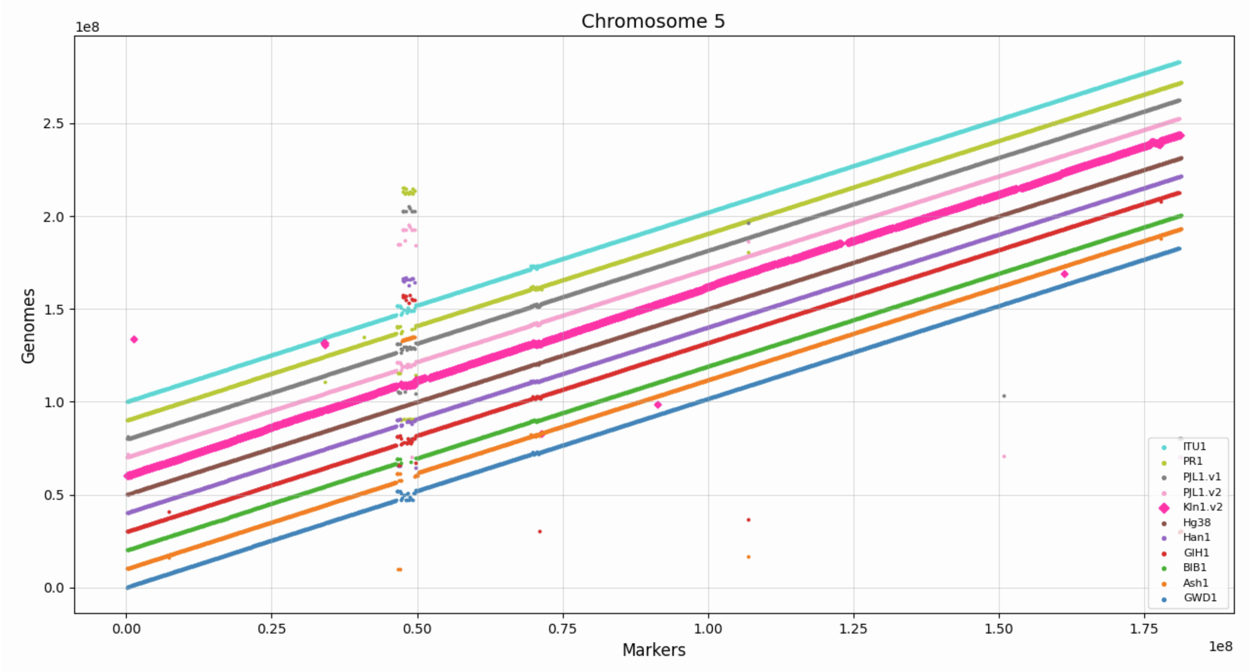

Chromosome 5
1e8
Genomes
Markers
1e8
0.0
0.5
1.0
1.5
2.0
2.5
0.00
0.25
0.50
0.75
1.00
1.25
1.50
1.75
ITU1
PR1
PJL1.v1
PJL1.v2
Kln1.v2
Hg38
Han1
GIH1
BIB1
Ash1
GWD1

(A)

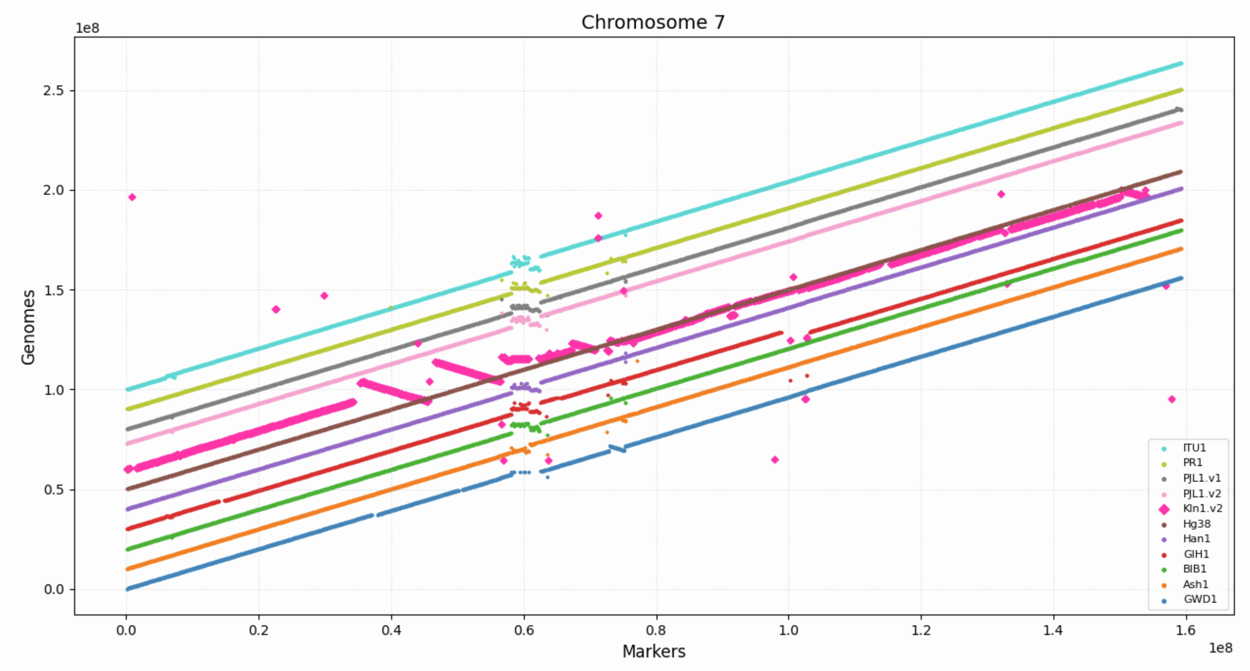

Chromosome 7
1e8
Genomes
Markers
1e8
0.0
0.5
1.0
1.5
2.0
2.5
0.0
0.2
0.4
0.6
0.8
1.0
1.2
1.4
1.6
ITU1
PR1
PJL1.v1
PJL1.v2
Kln1.v2
Hg38
Han1
GIH1
BIB1
Ash1
GWD1

(B)

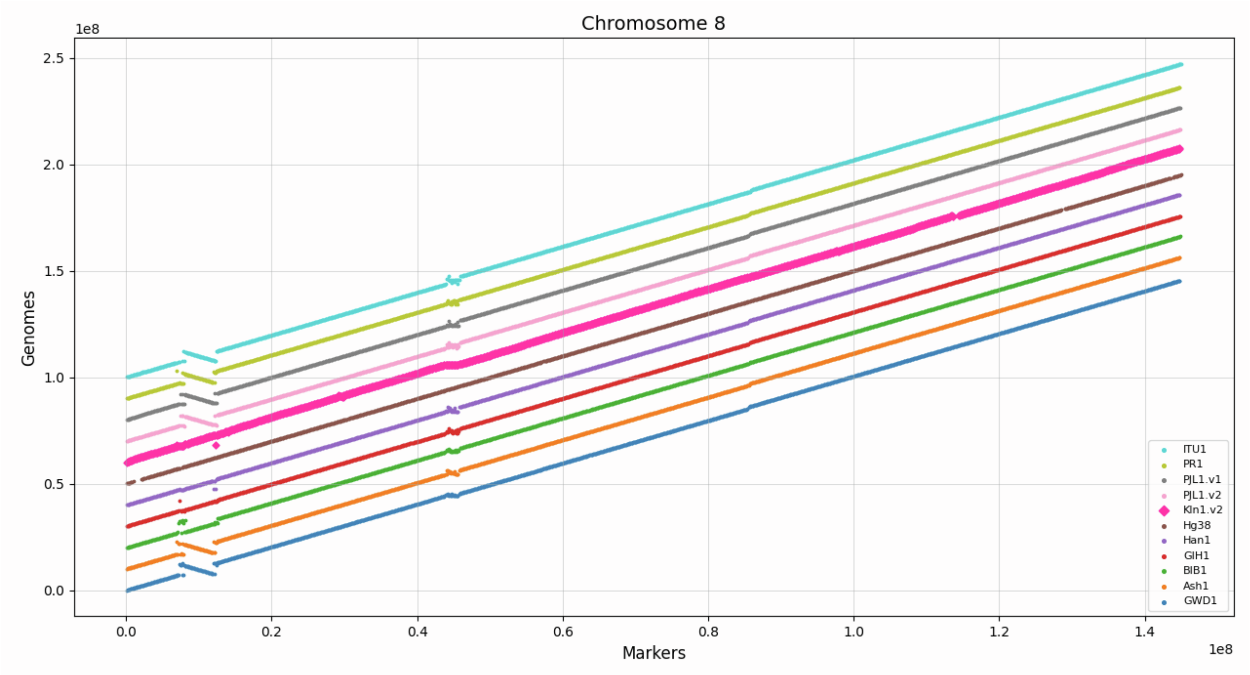

Chromosome 8
1e8
2.5
2.0
1.5
1.0
0.5
0.0
Genomes
0.0
0.2
0.4
0.6
0.8
1.0
1.2
1.4
Markers
1e8
ITU1
PR1
PJL1.v1
PJL1.v2
Kln1.v2
Hg38
Han1
GIH1
BIB1
Ash1
GWD1

(C)

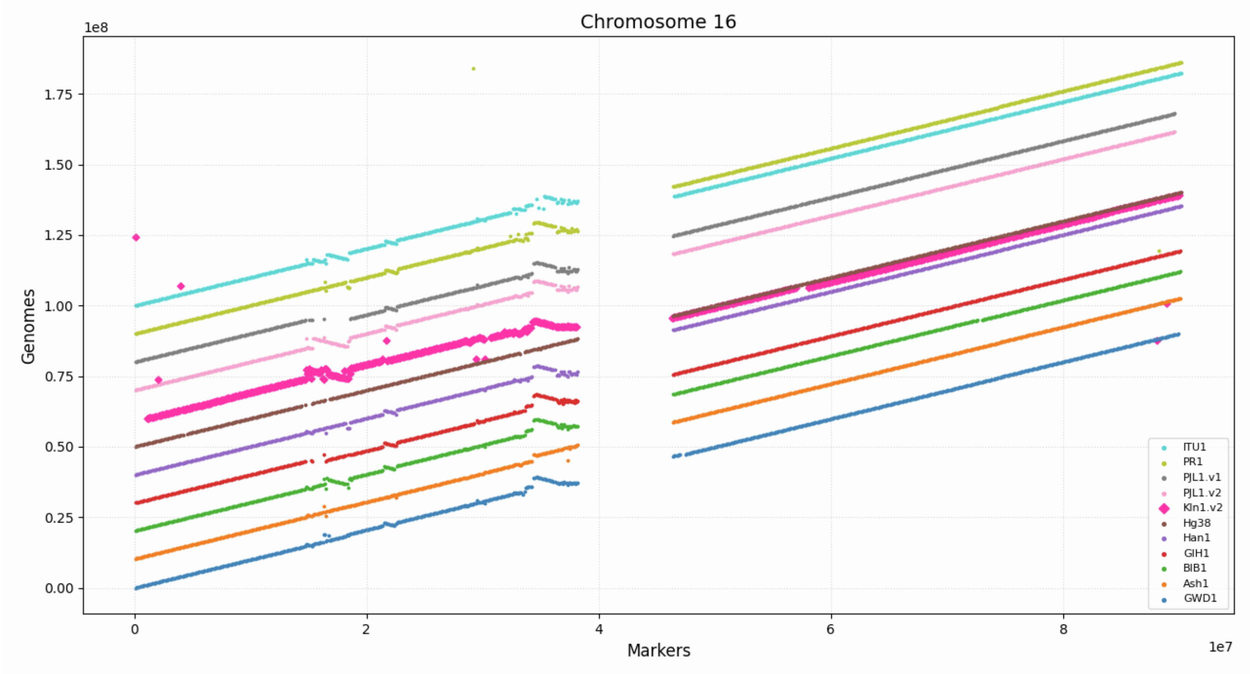

Chromosome 16
1e8
1.75
1.50
1.25
1.00
0.75
0.50
0.25
0.00
Genomes
0
2
4
6
8
Markers
1e7
ITU1
PR1
PJL1.v1
PJL1.v2
Kln1.v2
Hg38
Han1
GIH1
BIB1
Ash1
GWD1

(D)

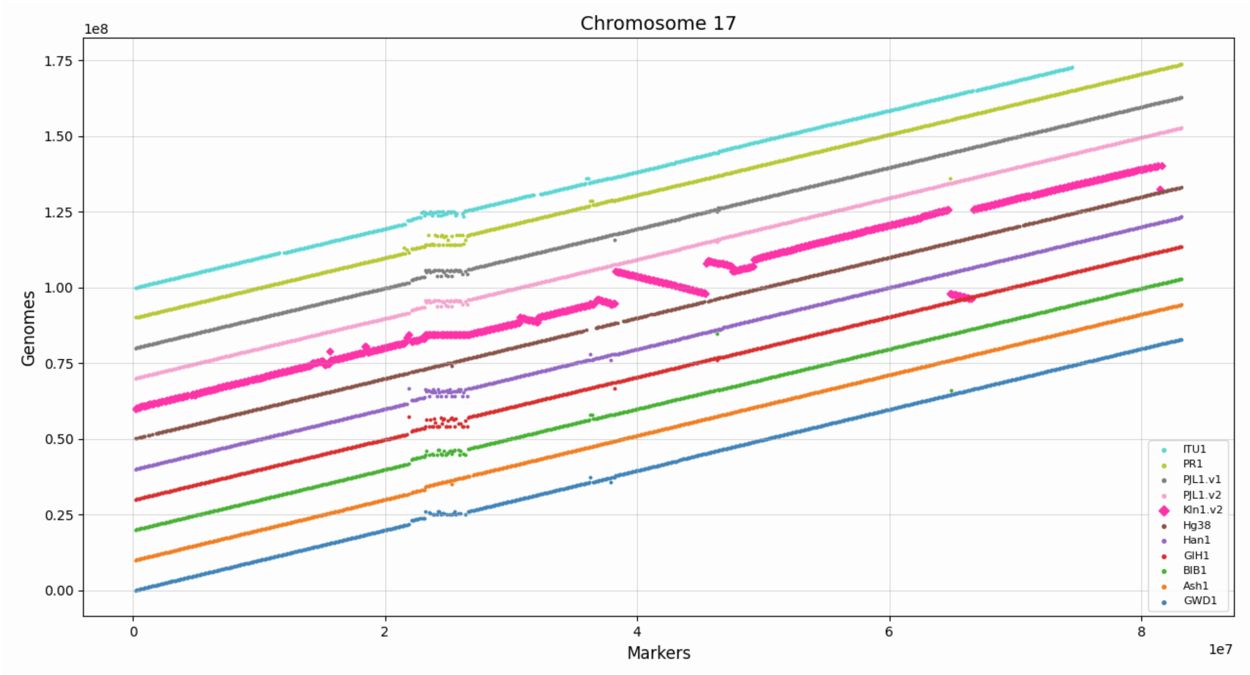

(E)

Supplementary Figure S2 (A-E): shows comparative dot plot of individuals' chromosomes from several genomes against virtual markers every 100kb from hg38 assembly.

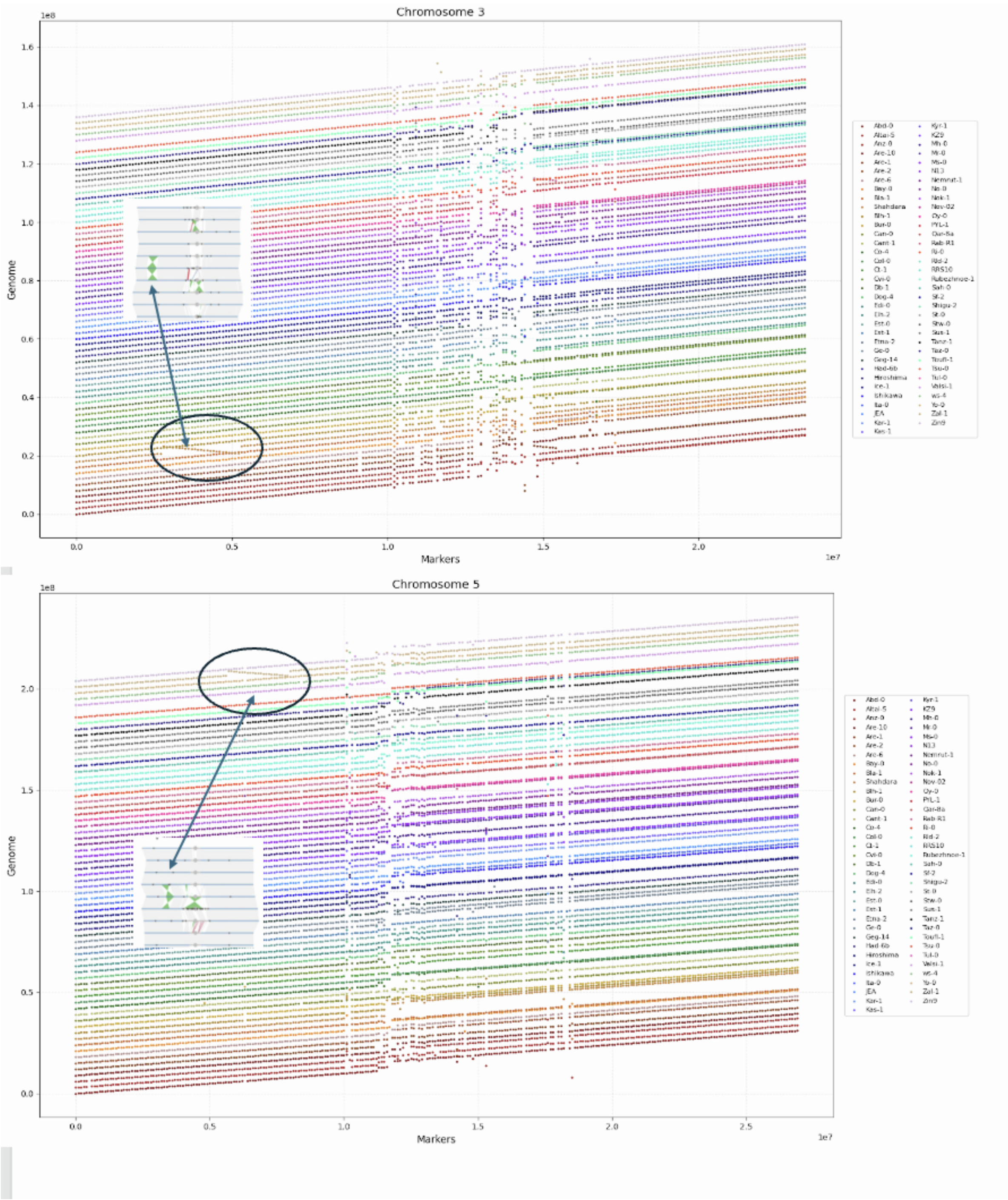

Supplementary Figure S3: Comparison of chromosome 3 and 5 of 69 accession of Arabidopsis (PMID: 38605175) compared to the virtual markers from the reference Arabidopsis genome.

(https://ftp.ncbi.nlm.nih.gov/genomes/all/GCF/000/001/735/GCF_000001735.4_TAIR10.1/GCF_000001735.4_TAIR10.1_genomic.fna.gz). Inset shows the part of Figure 3 from PMID: 38605175

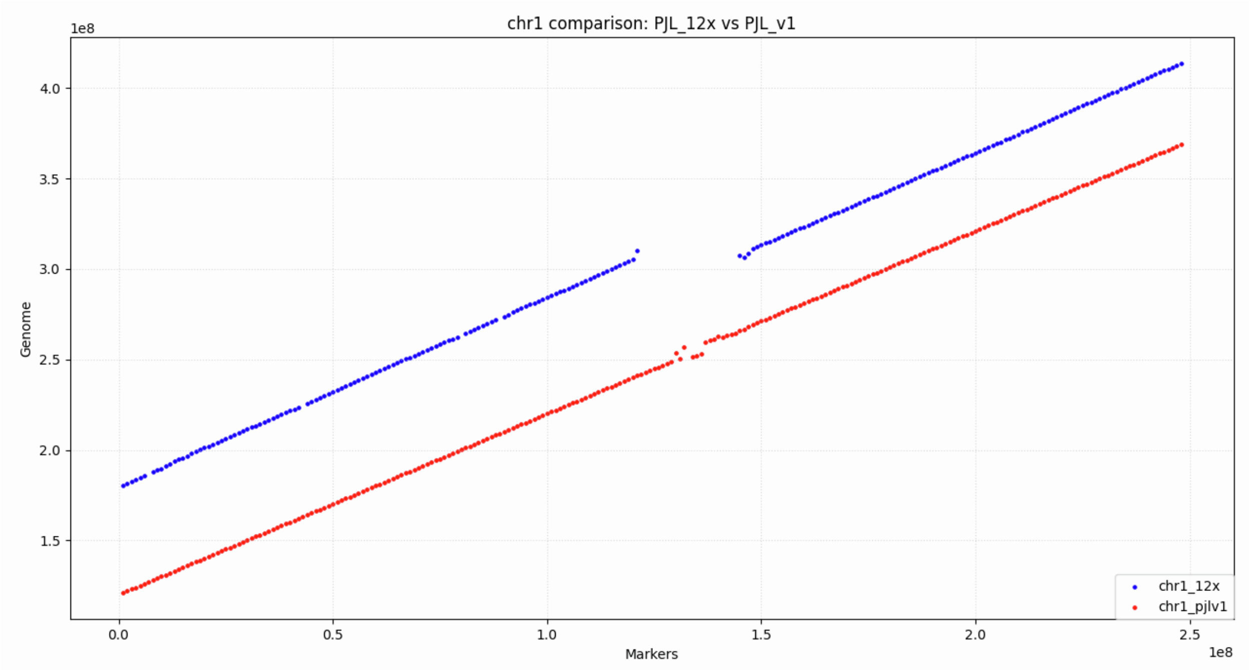
chr1 comparison: PJL_12x vs PJL_v1
1e8
4.0
3.5
3.0
2.5
2.0
1.5
Genome
0.0
0.5
1.0
1.5
2.0
2.5
1e8
Markers
chr1_12x
chr1_pjlv1

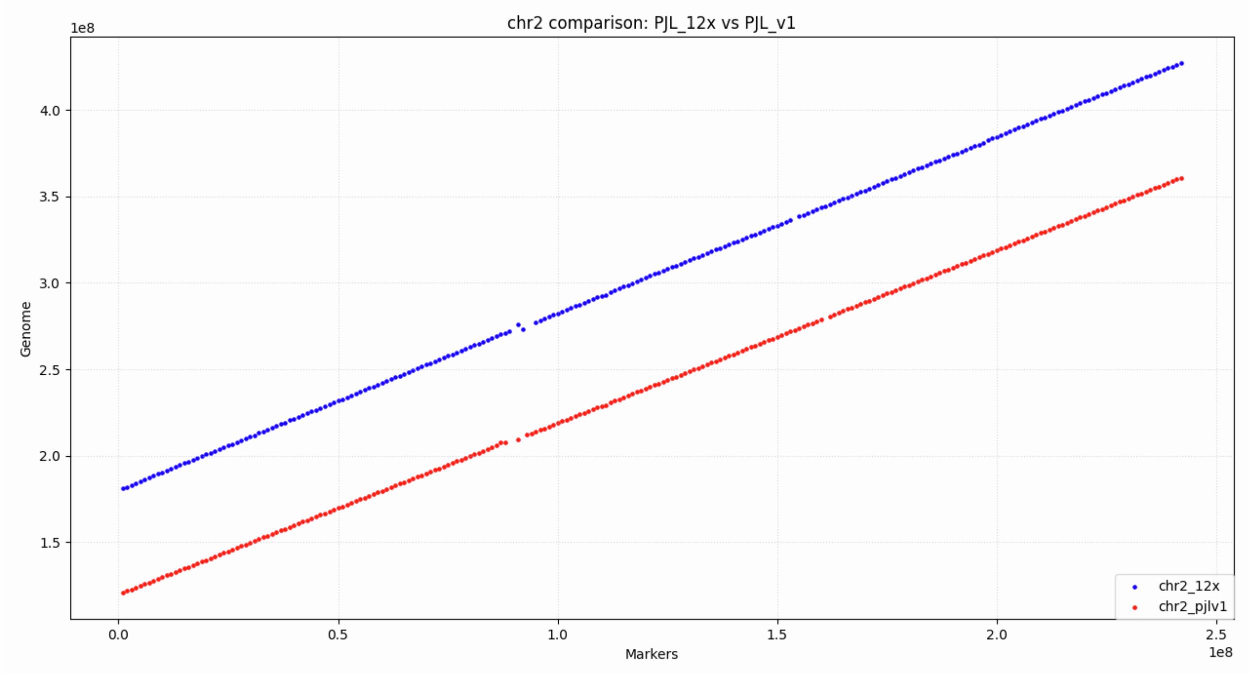
chr2 comparison: PJL_12x vs PJL_v1
1e8
4.0
3.5
3.0
2.5
2.0
1.5
Genome
0.0
0.5
1.0
1.5
2.0
2.5
1e8
Markers
chr2_12x
chr2_pjlv1

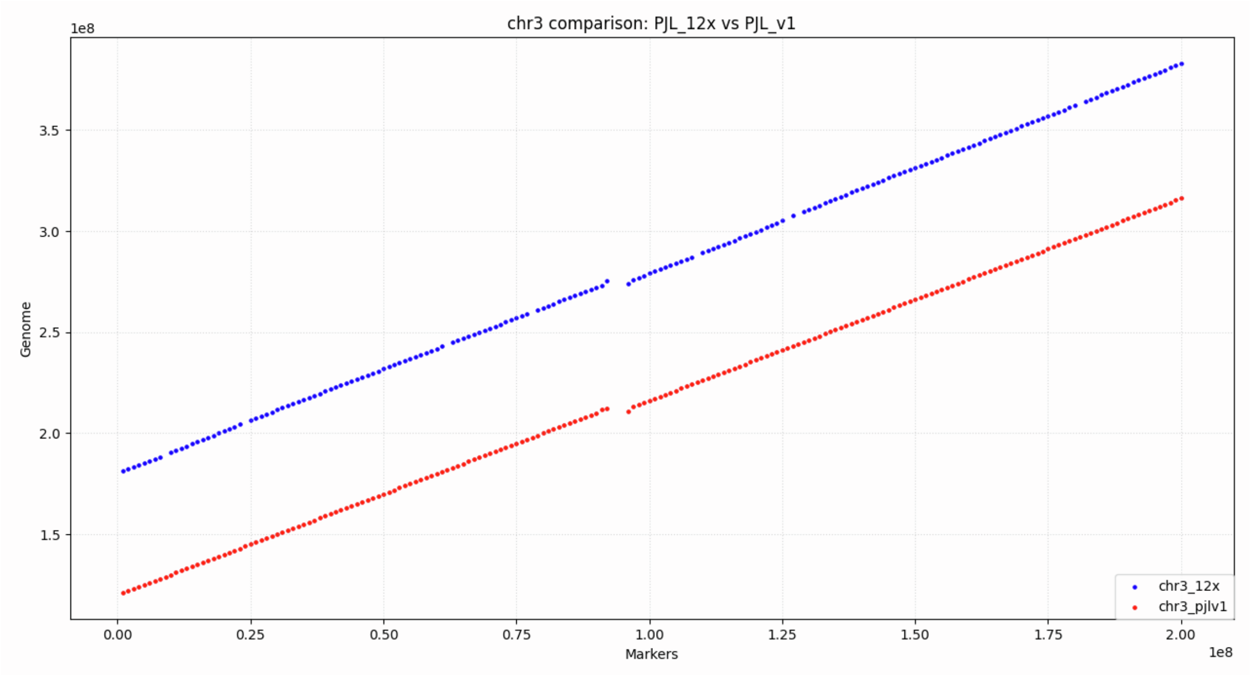

chr3 comparison: PJL_12x vs PJL_v1
1e8
3.5
3.0
2.5
2.0
1.5
Genome
0.00
0.25
0.50
0.75
1.00
1.25
1.50
1.75
2.00
1e8
Markers
chr3_12x
chr3_pjlv1

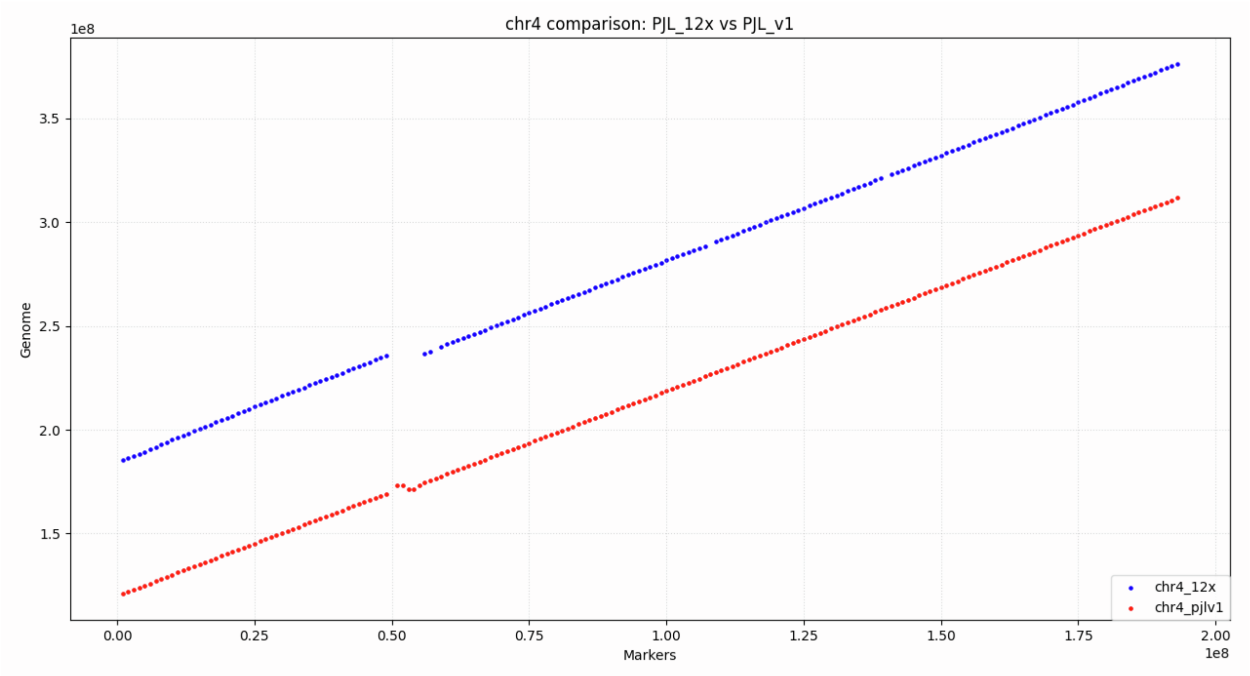

chr4 comparison: PJL_12x vs PJL_v1
1e8
3.5
3.0
2.5
2.0
1.5
Genome
0.00
0.25
0.50
0.75
1.00
1.25
1.50
1.75
2.00
1e8
Markers
chr4_12x
chr4_pjlv1

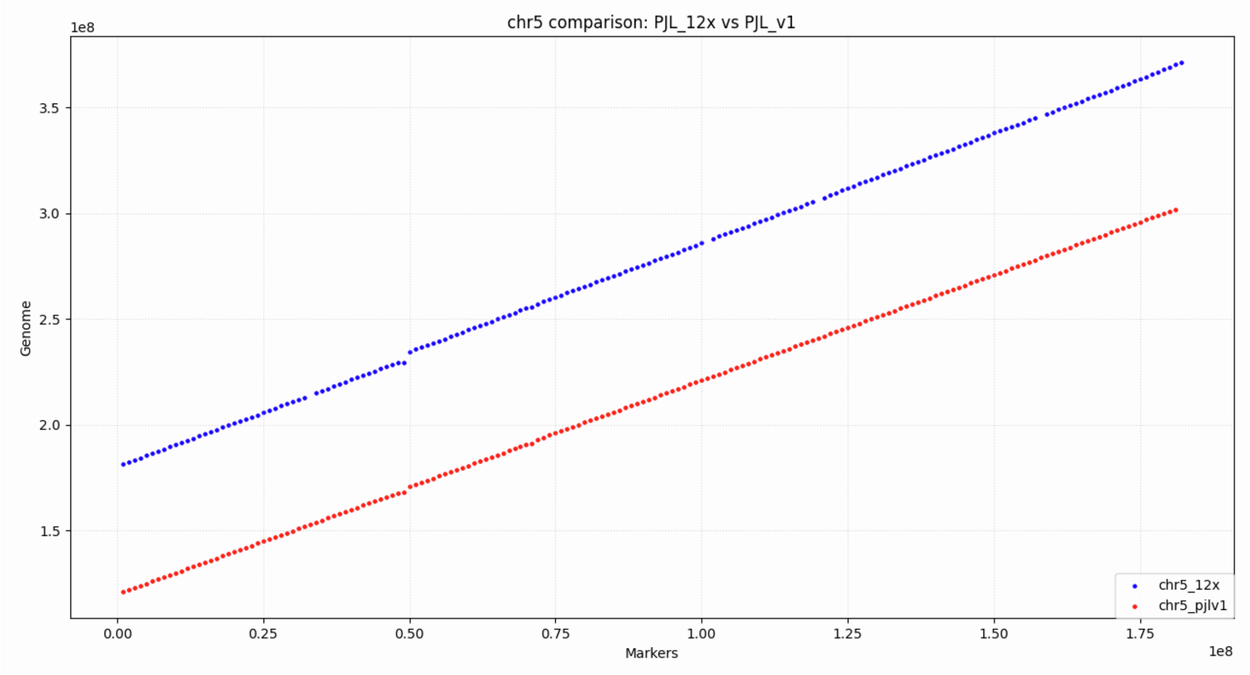
chr5 comparison: PJL_12x vs PJL_v1
1e8
3.5
3.0
2.5
2.0
1.5
Genome
chr5_12x
chr5_pjlv1
0.00
0.25
0.50
0.75
1.00
1.25
1.50
1.75
Markers
1e8

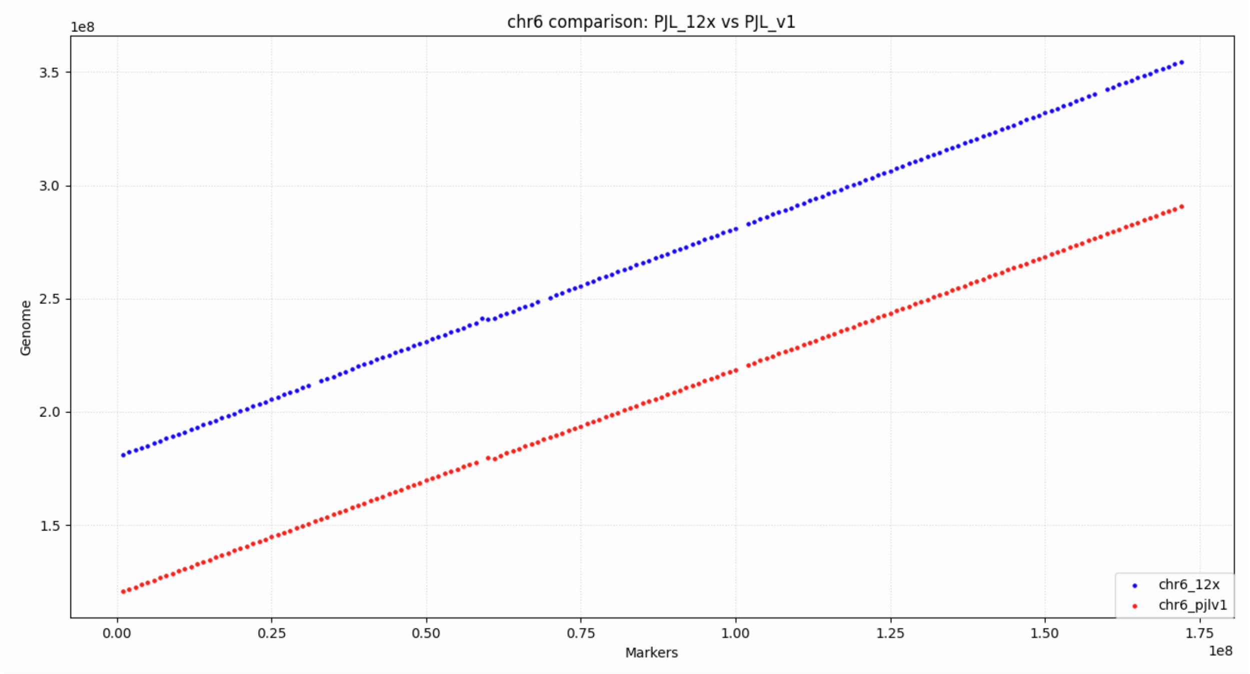
chr6 comparison: PJL_12x vs PJL_v1
1e8
3.5
3.0
2.5
2.0
1.5
Genome
chr6_12x
chr6_pjlv1
0.00
0.25
0.50
0.75
1.00
1.25
1.50
1.75
Markers
1e8

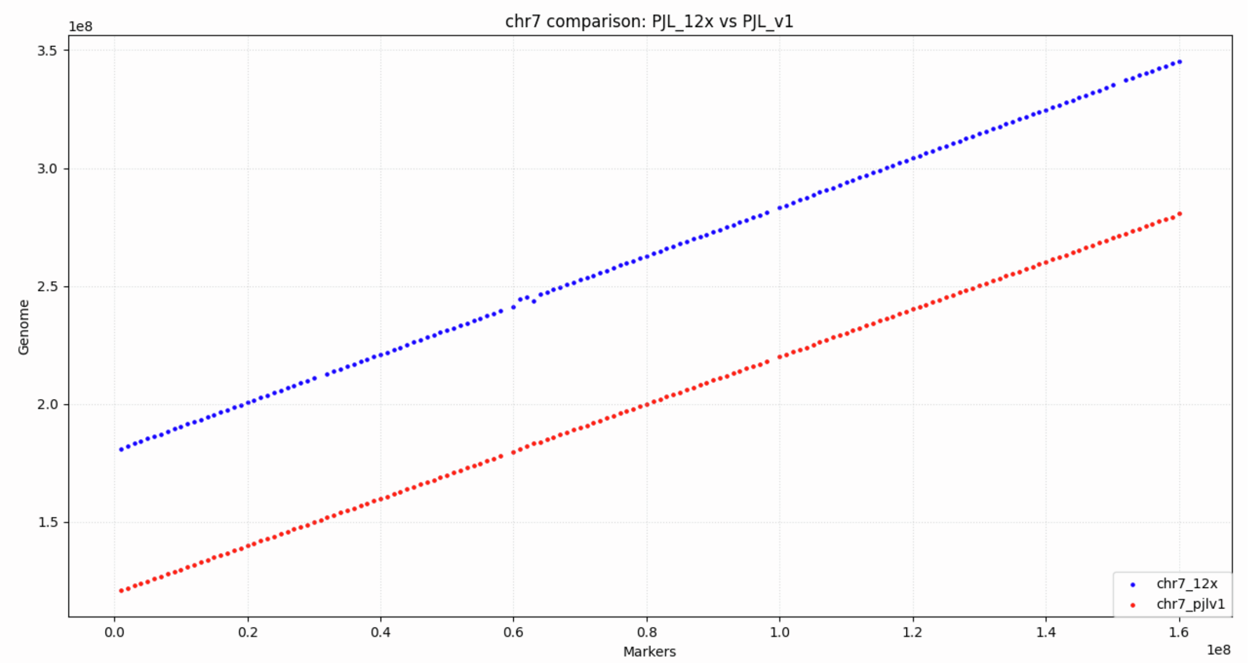
chr7 comparison: PJL_12x vs PJL_v1
1e8
3.5
3.0
2.5
2.0
1.5
Genome
0.0
0.2
0.4
0.6
0.8
1.0
1.2
1.4
1.6
1e8
Markers
chr7_12x
chr7_pjlv1

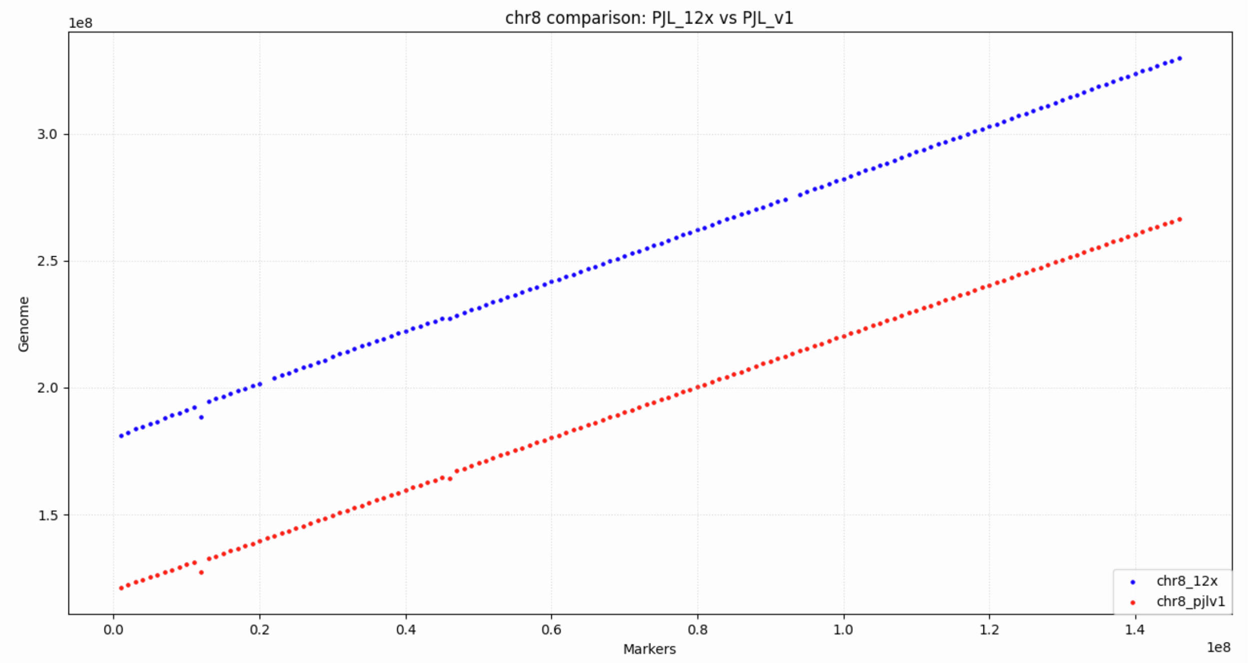
chr8 comparison: PJL_12x vs PJL_v1
1e8
3.0
2.5
2.0
1.5
Genome
0.0
0.2
0.4
0.6
0.8
1.0
1.2
1.4
1e8
Markers
chr8_12x
chr8_pjlv1

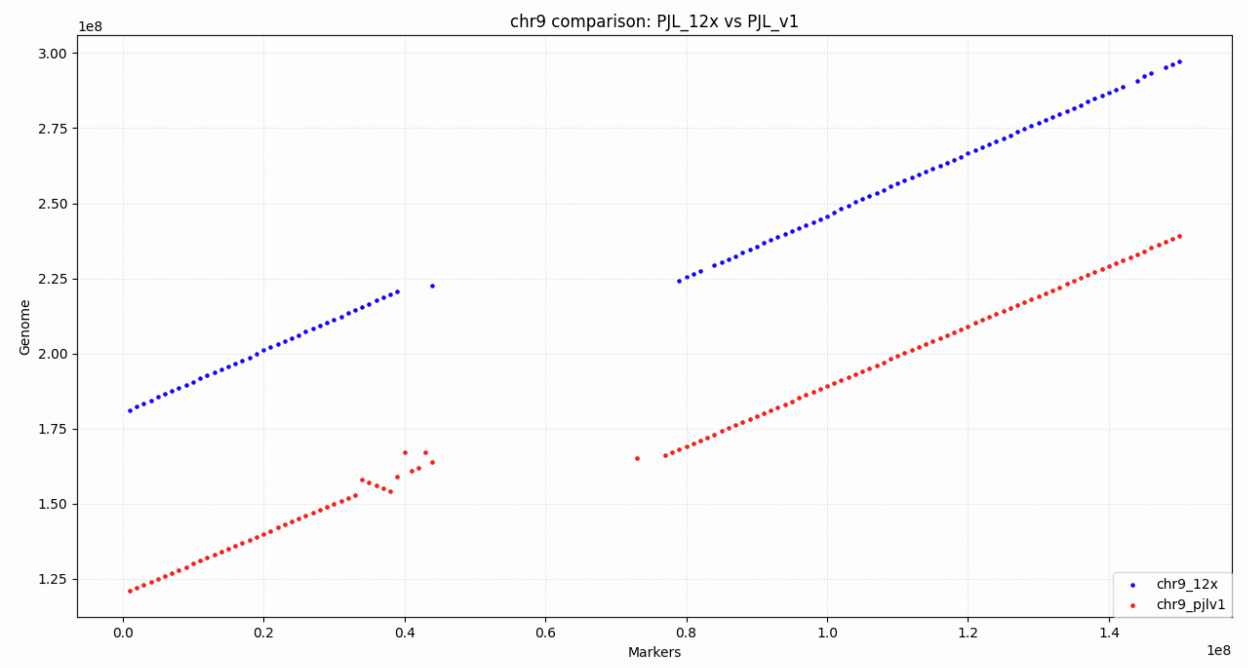
chr9 comparison: PJL_12x vs PJL_v1
1e8
3.00
2.75
2.50
2.25
2.00
1.75
1.50
1.25
Genome
0.0
0.2
0.4
0.6
0.8
1.0
1.2
1.4
Markers
1e8
chr9_12x
chr9_pjlv1

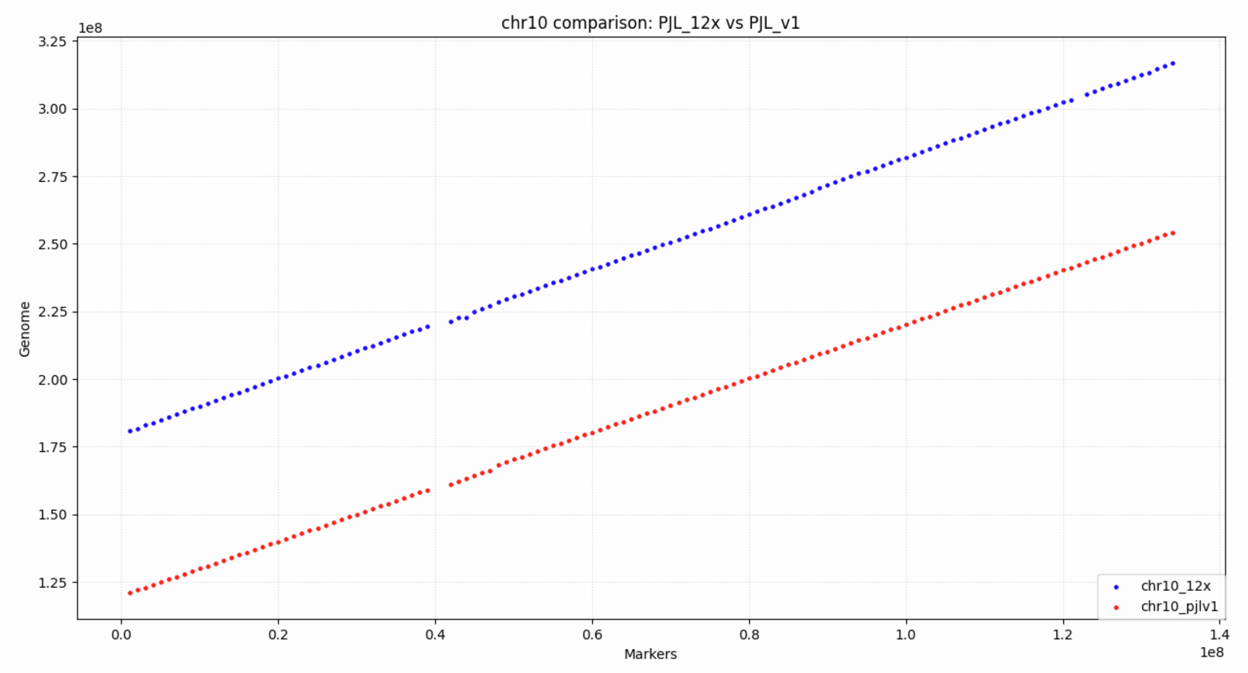
chr10 comparison: PJL_12x vs PJL_v1
1e8
3.25
3.00
2.75
2.50
2.25
2.00
1.75
1.50
1.25
Genome
0.0
0.2
0.4
0.6
0.8
1.0
1.2
1.4
Markers
1e8
chr10_12x
chr10_pjlv1

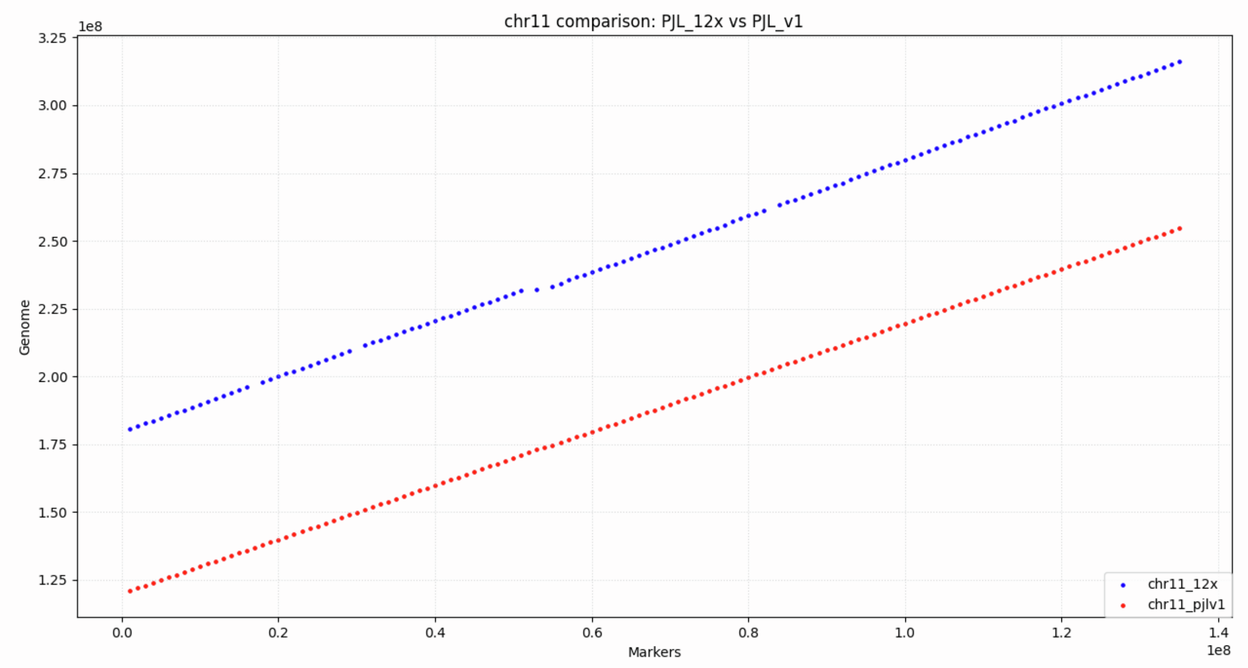
chr11 comparison: PJL_12x vs PJL_v1
1e8
3.25
3.00
2.75
2.50
2.25
2.00
1.75
1.50
1.25
Genome
0.0
0.2
0.4
0.6
0.8
1.0
1.2
1.4
1e8
Markers
chr11_12x
chr11_pjlv1

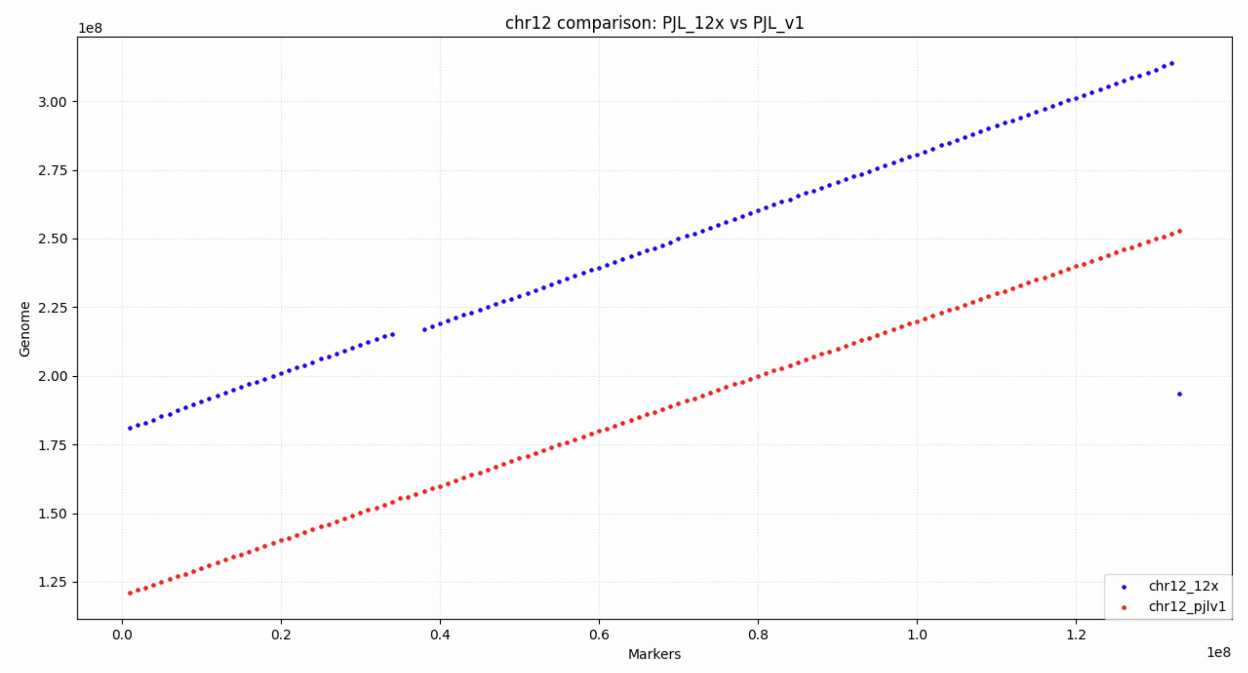
chr12 comparison: PJL_12x vs PJL_v1
1e8
3.00
2.75
2.50
2.25
2.00
1.75
1.50
1.25
Genome
0.0
0.2
0.4
0.6
0.8
1.0
1.2
1e8
Markers
chr12_12x
chr12_pjlv1

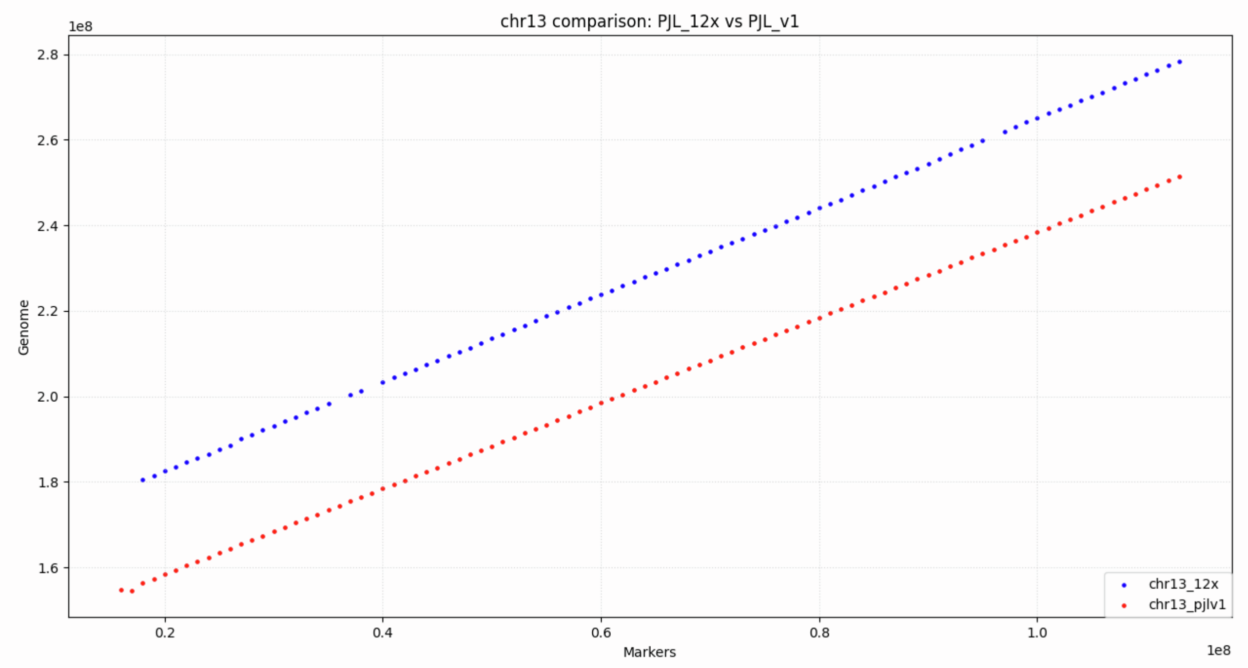
chr13 comparison: PJL_12x vs PJL_v1
1e8
2.8
2.6
2.4
2.2
2.0
1.8
1.6
Genome
0.2
0.4
0.6
0.8
1.0
Markers
1e8
chr13_12x
chr13_pjlv1

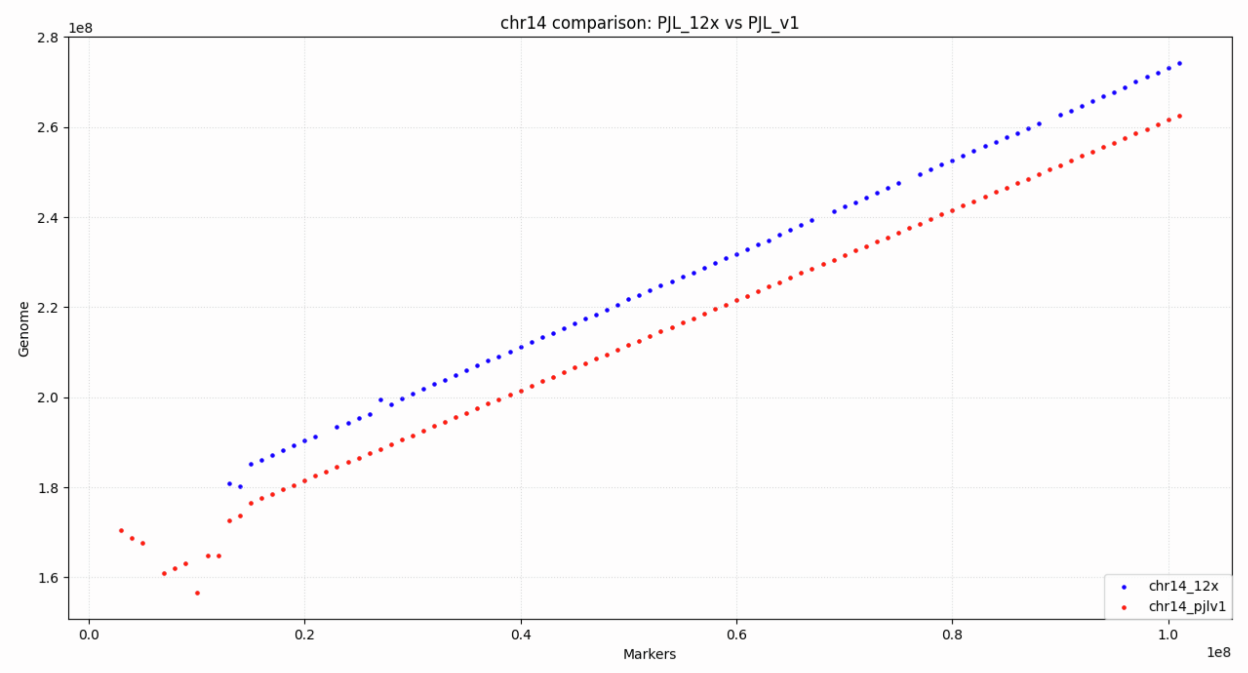
chr14 comparison: PJL_12x vs PJL_v1
1e8
2.8
2.6
2.4
2.2
2.0
1.8
1.6
Genome
0.0
0.2
0.4
0.6
0.8
1.0
Markers
1e8
chr14_12x
chr14_pjlv1

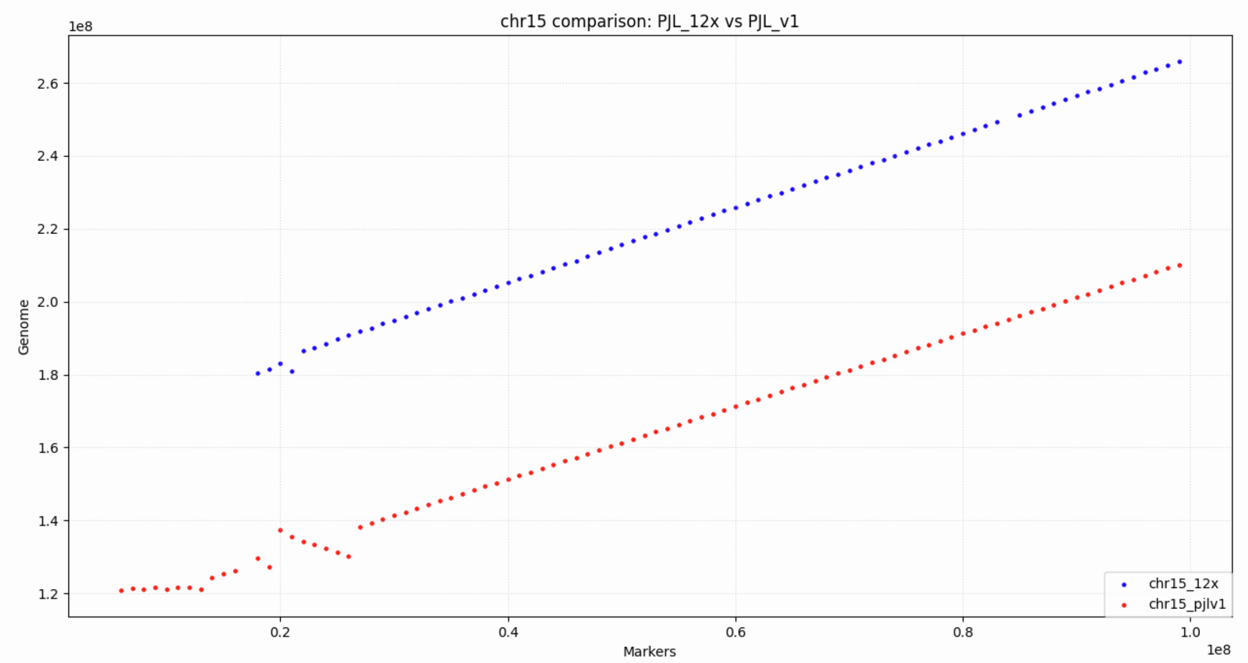
chr15 comparison: PJL_12x vs PJL_v1
1e8
2.6
2.4
2.2
2.0
1.8
1.6
1.4
1.2
Genome
0.2
0.4
0.6
0.8
1.0
1e8
Markers
chr15_12x
chr15_pjlv1

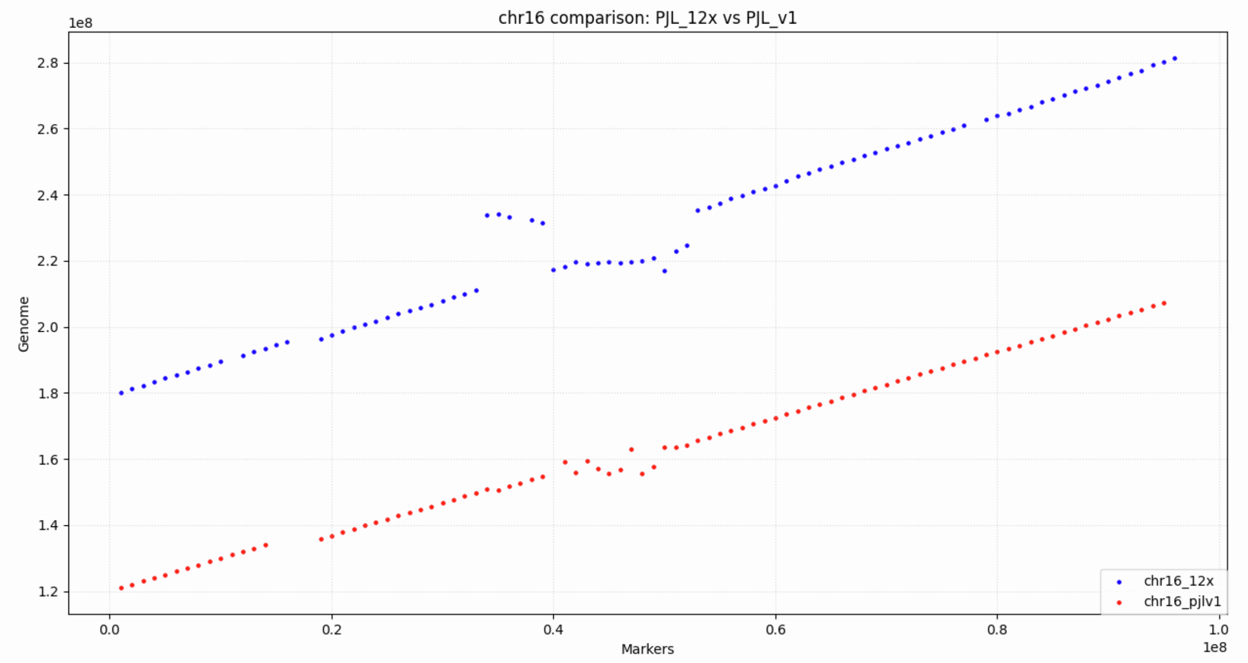
chr16 comparison: PJL_12x vs PJL_v1
1e8
2.8
2.6
2.4
2.2
2.0
1.8
1.6
1.4
1.2
Genome
0.0
0.2
0.4
0.6
0.8
1.0
1e8
Markers
chr16_12x
chr16_pjlv1

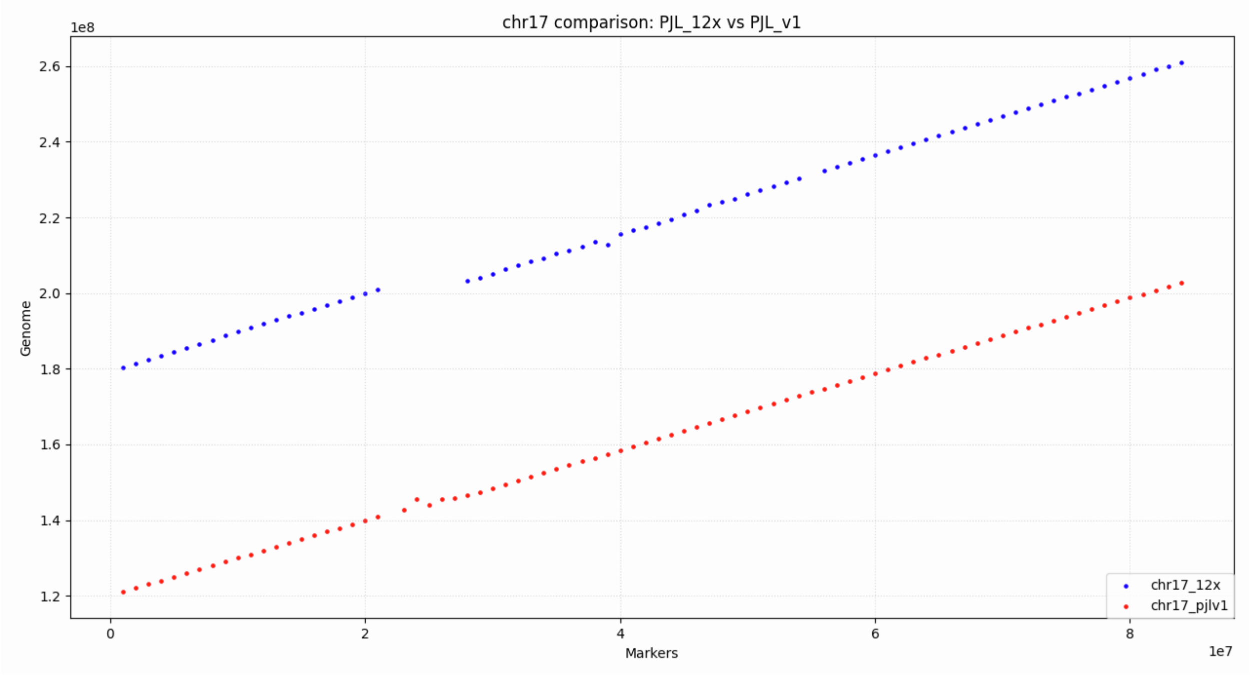
chr17 comparison: PJL_12x vs PJL_v1
1e8
2.6
2.4
2.2
2.0
1.8
1.6
1.4
1.2
Genome
0
2
4
6
8
1e7
Markers
chr17_12x
chr17_pjlv1

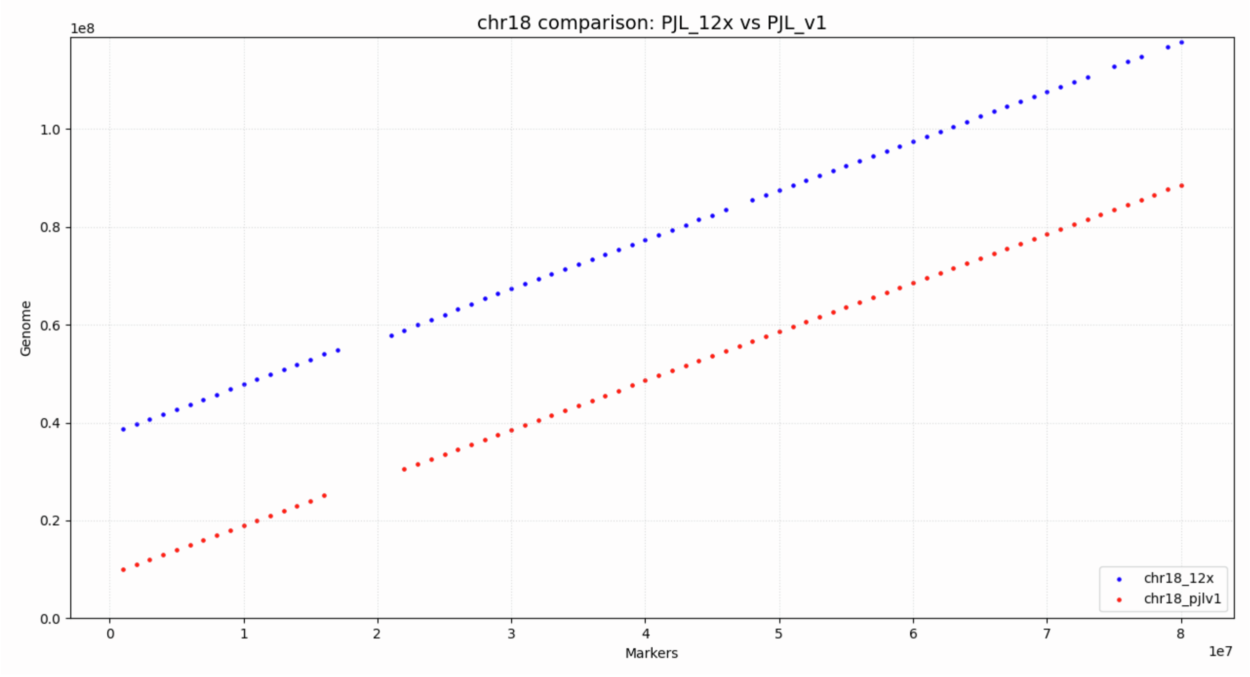
chr18 comparison: PJL_12x vs PJL_v1
1e8
1.0
0.8
0.6
0.4
0.2
0.0
Genome
0
1
2
3
4
5
6
7
8
1e7
Markers
chr18_12x
chr18_pjlv1

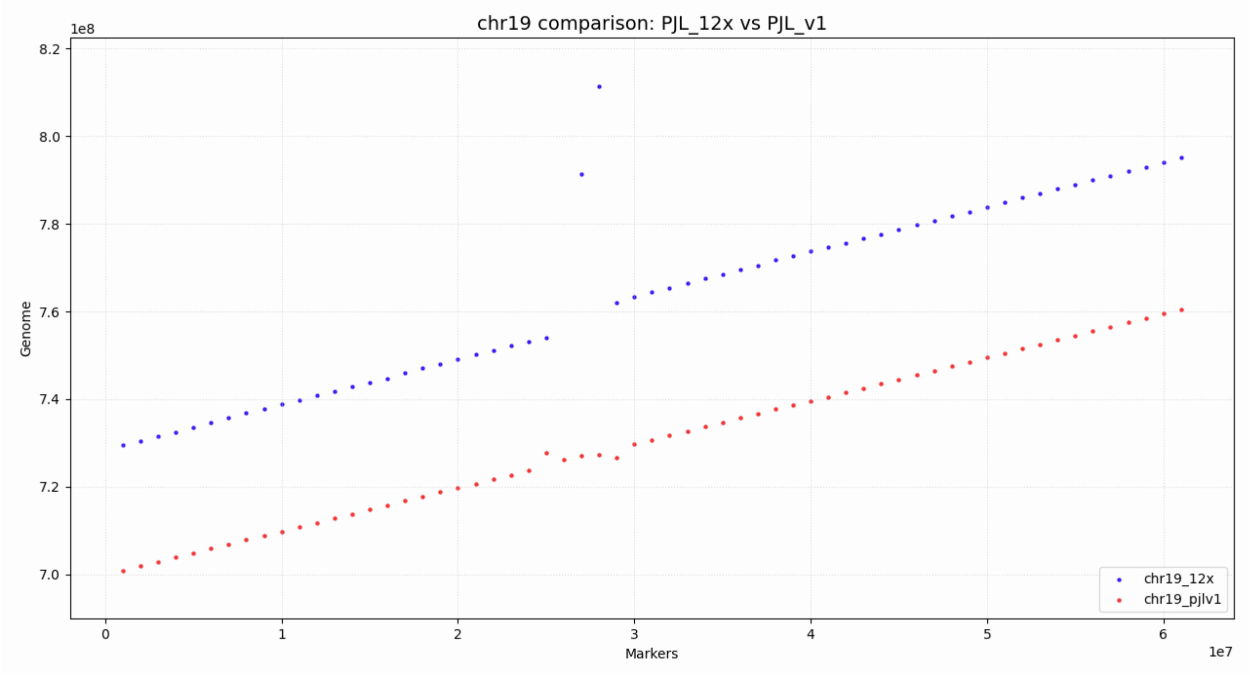
chr19 comparison: PJL_12x vs PJL_v1
1e8
8.2
8.0
7.8
7.6
7.4
7.2
7.0
Genome
0
1
2
3
4
5
6
Markers
1e7
chr19_12x
chr19_pjlv1

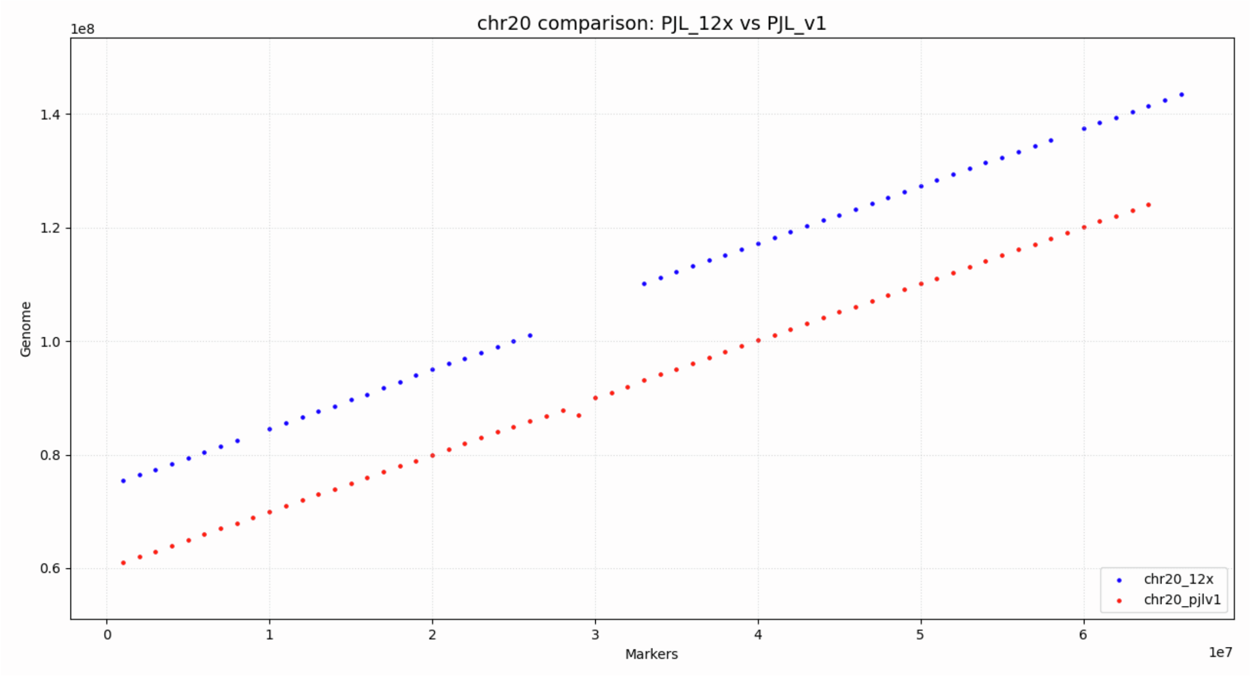
chr20 comparison: PJL_12x vs PJL_v1
1e8
1.4
1.2
1.0
0.8
0.6
Genome
0
1
2
3
4
5
6
Markers
1e7
chr20_12x
chr20_pjlv1

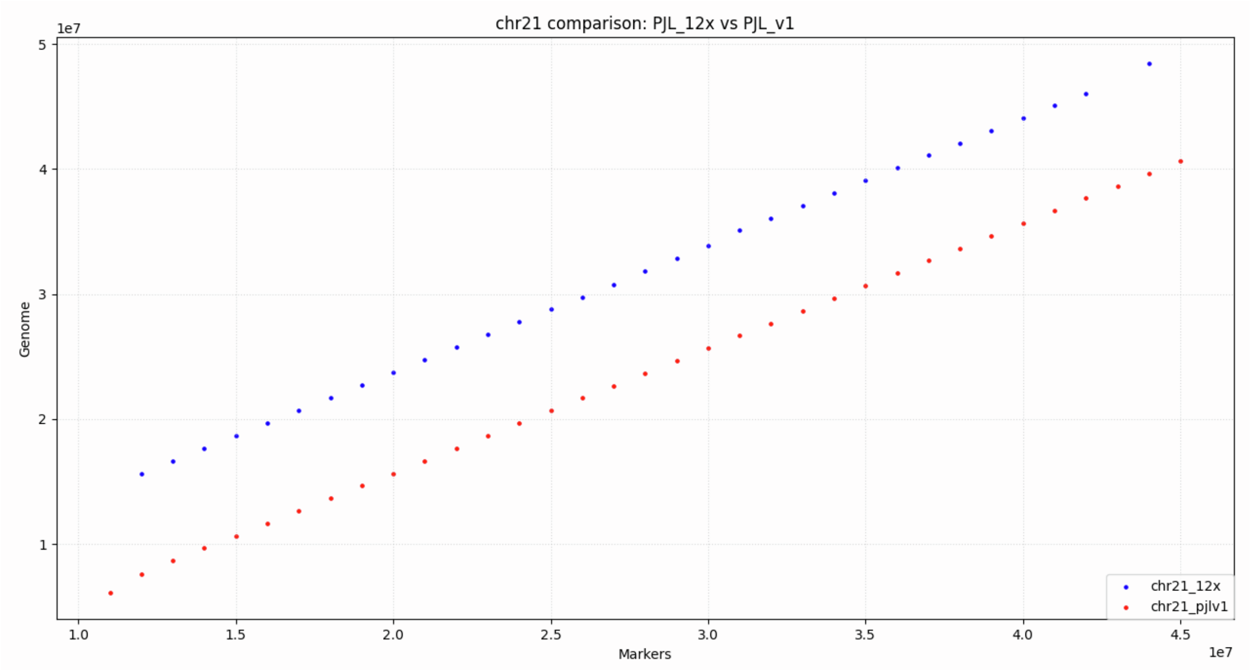
chr21 comparison: PJL_12x vs PJL_v1
1e7
Genome
Markers
1e7
chr21_12x
chr21_pjlv1

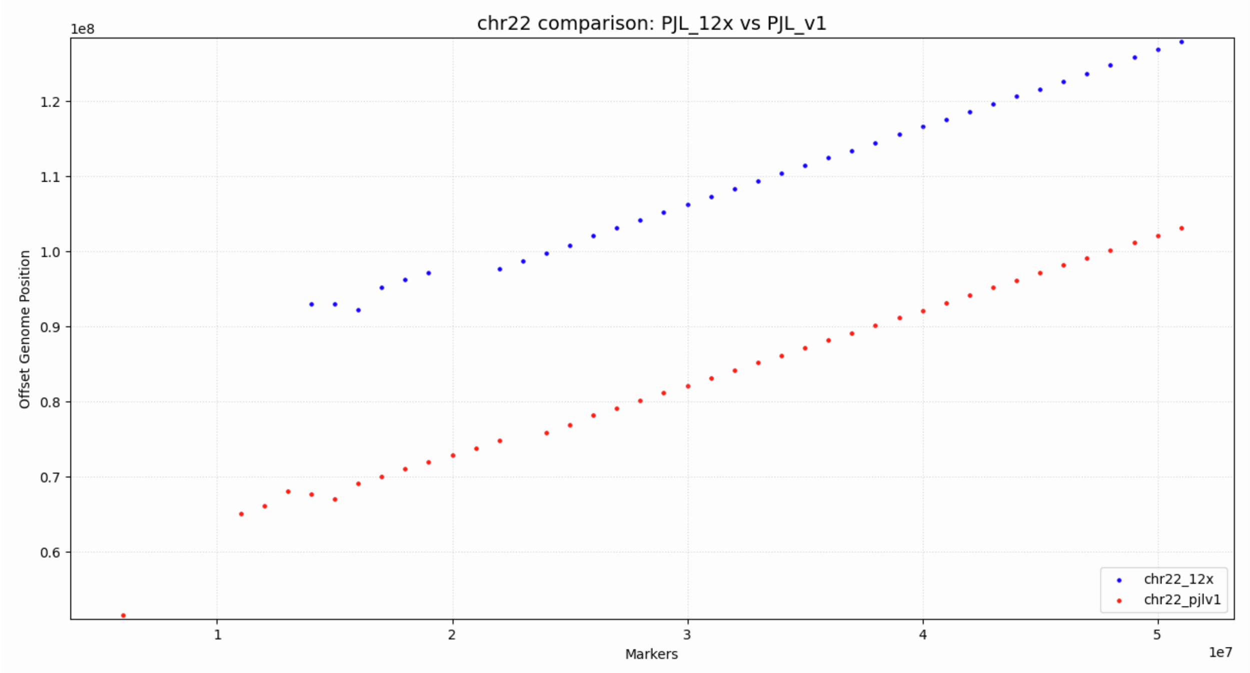
chr22 comparison: PJL_12x vs PJL_v1
1e8
Offset Genome Position
Markers
1e7
chr22_12x
chr22_pjlv1

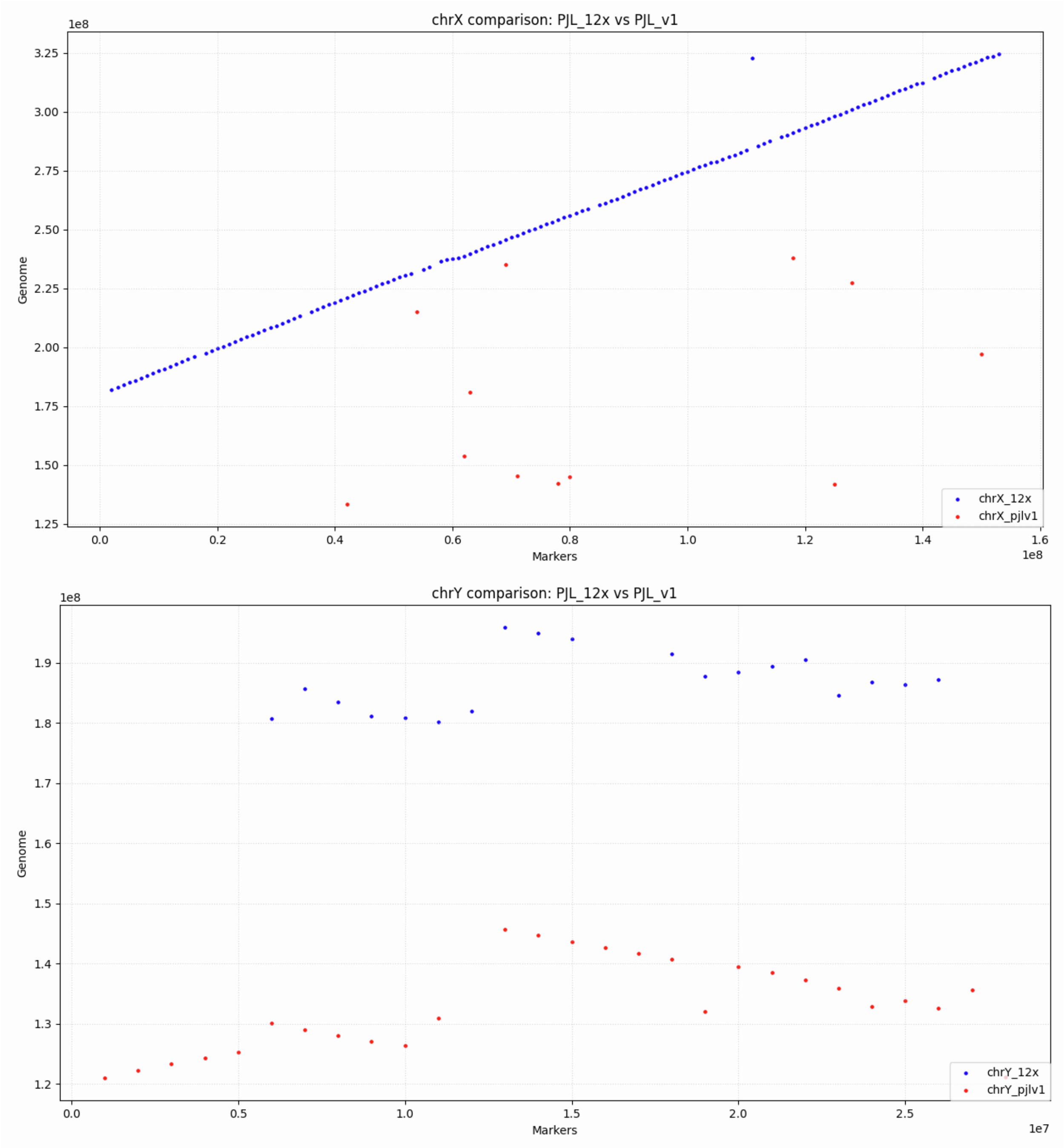

Supplementary Figure S4: Comparison of PJL1.12x against PJL1.v1: Bench marking results for 12x coverage by down-sampled assembly from 30x raw reads of PJL1

Supplementary Table S1:

rsID	Location	Expected Allele	hg38 Allele	Spanish homoIN V	T2T Allele	KIn2 Allele	BIB1 Allele	GIH1 Allele	Han1 Allele	ITU1 Allele	PJL_ITU Allele	PJL_Pat Allele	PR1 Allele	Ash1 Allele	GWD1 Allele
rs17627505	chr8:8656549	C>G/C>T	C	C	C	C	C	T	C	C	C	C	C	C	C
rs10503393	chr8:8744686	C>A/C>G	C	G	C	C	C	C	C	C	C	C	C	C	C
rs2428	chr8:8783635	C>A/C>G/C>T	C	T	C	T	C	T	T	C	C	C	C	C	T
rs11774860	chr8:8794369	C>A/C>T	C	C	T	C	C	C	C	C	C	C	C	C	C
rs3827811	chr8:8801528	C>G/C>T	C	G	T	T	C	C	C	T	T	T	C	T	C
rs171547	chr8:8802049	A>G	A	A	A	A	G	A	A	G	G	G	A	G	A
rs1876836	chr8:8824246	T>A/T>C/T>G	T	G	C	C	T	C	C	T	T	T	T	C	C
rs1039916	chr8:8828344	A>C/A>G/A>T	A	G	A	G	A	G	G	A	A	A	A	A	G
rs1178061	chr6: 50946658	C>T	C	T	T	C	C	C	T	C(Hits on chr8)	C(Hits on chr8)	C	T	C	T
rs1178247	not available			C											
rs3885690	chr8:10969359	T>C/T>G	T	A	C	No hits	T	T	T	C		C	C	T	T
rs2409691	chr8:11085766	C>A/C>T/C>G	C	C	T	C	T	C	C	T	T	T	T	T	T
rs13266785	chr8:11132011	G>A	G	G	A	G	A	G	G	G	A	A	A	A	G
rs10282848	chr8:11139157	A>G	A	A	A	A	G	A	A	A	A	A	A	A	A
rs10503417	chr8:11139303	G>A	G	G	A	G	G	G	G	G	A	A	A	A	G
rs2409719	chr8:11159379	A>C/A>G	A	A	G	A	A	A	A	A	G	G	A	A	A

Supplementary Methods:

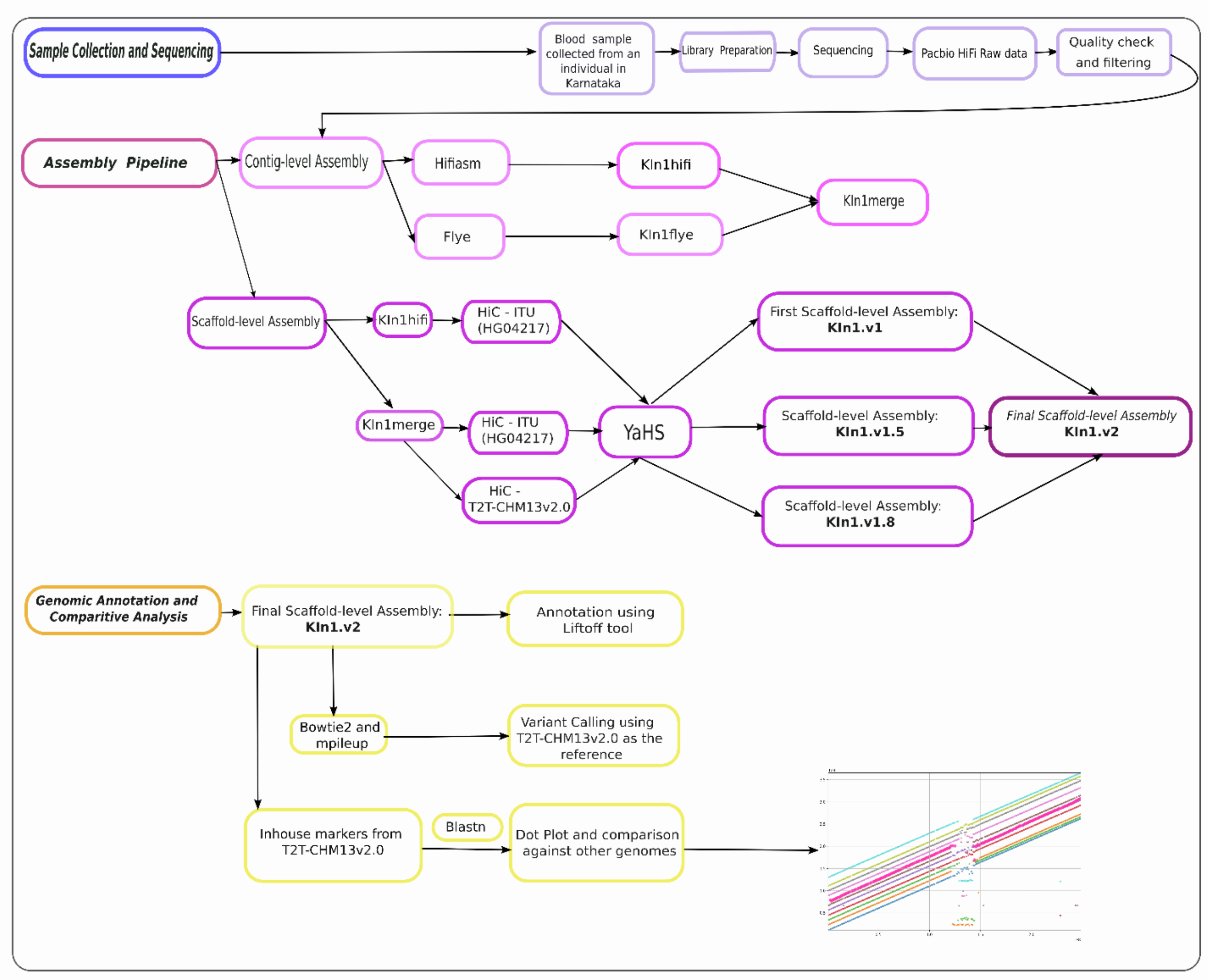

Methodology Flowchart

Commands used in Methods Section:

Liftoff:

- liftofftools variants -r <reference>.fa -t <target.fa> -rg c<reference>.gff3 -tg<target>.gff3
- liftofftools synteny -r <reference>.fa -t <target>.fa -rg<reference>.gff3 -tg<target>.gff3

Seqtk:

- seqtk sample -s100 merged.ccs.fastq.gz N > 12x_merge_ccs.fastq.
