# Supplementary material for: The Karnataka Individual Genome Project expands the human reference landscape to include South Asia
Source: HGG Adv. 2025 Sep 18;7(1):100516. doi: 10.1016/j.xhgg.2025.100516 (PMC12513208; doi:10.1016/j.xhgg.2025.100516)
Supplement: Document S2. Article plus supplemental information [file mmc2.pdf]

HGG Advances

ARTICLE

The Karnataka Individual Genome Project expands the human reference landscape to include South Asia

Apoorva Ganesh,[1] Anisha Mhatre,[1] Yash Chindarkar,[1] Moushmi Goswami,[1] Prakruti Mishra,[1] Aditya Sharma,[1] Manjushri Kalpande,[1] Febina Ravindran,[1] Subhashini Srinivasan,[1,2,*] and Bibha Choudhary[1,*]

Summary

Assembling individual genomes remains an expensive endeavor, hindering large-scale comparative human genomics. So far, chromosome-level assemblies of only a few individuals, including PR1 (Puerto Rican), Ash1 (Ashkenazi Jew), Han1 (Southern Han Chinese), and CHM13 (Northern European) have been reported. Here, we present a chromosome-level genome assembly of a non-International Genome Sample Resource (IGSR) and non-Genome in a Bottle individual from the Indian subcontinent (KIn1) obtained using a cost-effective approach. We achieved an N50 of 141 Mb and an L50 of 9—very close to the maximum achievable N50 of 147 Mb and minimum achievable L50 of 8, respectively, for human genomes. We also generated chromosome-level assemblies for other individuals from the Indian diaspora, including PJL1 from Punjab, Lahore (HG03492), GIH1 from Gujarat (NA20847), BIB1 from Bangladesh (HG03009), and ITU1 from Andhra Pradesh (HG04217), all represented in IGSR, by scaffolding the publicly available respective contigs and Hi-C data. Here, we demonstrate that by comparing these individual genomes with those reported elsewhere, the configuration of inversion 8p23.1 in KIn1, Han1, GIH1, and BIB1 is similar to that in hg38, here to referred as 8p23.1std. The inverted configuration, 8p23.1inv, is present in CHM13, PJL1, Ash1, and PR1. We also find evidence of all three large known inversions in the p-arm of chromosome 16, with prevalence among South Asians. In chromosome 5, one of the reported inversions is present in all assemblies except hg38 and Ash1. Finally, we investigate the large inversions that are unique to KIn1.

Introduction

The order of the euchromatic regions of the human genome was first deciphered and reported by two independent groups in 2001.[1,2] This momentous achievement led to the envisioning of a day when individuals' genomes could be deciphered for as little as $1,000. With substantial funding to fulfill this vision, sequencing technologies now allow chromosomal-level assembly of individual genomes for as low as tens of thousands of US dollars. This is a considerable achievement, considering the first human genome assembly cost $3 billion US dollars. Additionally, it took 2 decades to close numerous gaps in the genome published in 2001; the current version is known as GRCh38 (Genome Reference Consortium human build 38).[3] The improved version, a mosaic of several individuals, still retains hundreds of gaps, including those at centromeres and the short arms of acrocentric chromosomes.

More recently, advances in second-generation long-read sequencing technologies have made a gapless assembly of individual genomes a reality. A telomere-to-telomere (T2T-CHM13v2.0) assembly of a Northern European individual (CHM13) offers a new reference against which other individual genomes can now be compared.[4]

Currently, chromosome-level genomes of three individuals have been reported, including those from a Puerto Rican individual (PR1),[5] a Southern Han Chinese individual (Han1),[6] and an Ashkenazi Jew (Ash1),[7] all annotated against the GRCh38/T2T-CHM13v2.0 reference. Table 1 compares sequencing technologies, coverage, and assembly methods used in these reports and those presented here.

The reports from the three individual genome assembly efforts completed so far and reviewed above underscore the high cost of sequencing using diverse technologies and assembling using non-standard pipelines. These reports suggest that PacBio HiFi reads are both necessary and sufficient to obtain high-quality contig-level assemblies. During scaffolding and gap-filling, these efforts mixed individual genomes with DNA sequences from the reference. Furthermore, manual corrections used in these reports are tedious and do not scale well. In 2021, an effort to optimize the methodology for human genome assemblies demonstrated that PacBio HiFi reads at 30× coverage, along with Hi-C reads from the same individual, are sufficient to achieve chromosome-level phased assemblies,[8] without the need for pedigree data or raw reads from other technologies. Additionally, using only high-coverage PacBio long-reads (not HiFi) for generating

[1]Institute of Bioinformatics and Applied Biotechnology, Biotech Park, GN Ramachandran Rd, Electronic City Phase I, Electronic City, Bengaluru, Karnataka 560100, India
[2]Lead contact
*Correspondence: ssubha@ibab.ac.in (S.S.), vibha@ibab.ac.in (B.C.)
https://doi.org/10.1016/j.xhgg.2025.100516.
© 2025 The Author(s). Published by Elsevier Inc. on behalf of American Society of Human Genetics.
This is an open access article under the CC BY license (http://creativecommons.org/licenses/by/4.0/).

Table 1. Assembly statistics, coverage, and assembly methods used for individual genomes reported here

Genome	Accession	Ethnicity/sample	Genome size, Gb	Scaffold N50, Mb	Sequencing technologies	Method to obtain contigs	Method to obtain scaffolds	Gap filling	Manual correction
KIn1.v1[a]	None	South Asian (Indian)/ blood from an individual	2.8	137	PacBio HiFi (12×) + HiC from HG04217	Hifiasm	YaHS	no	yes
KIn1.v2[a]			2.9	141	PacBio HiFi (12×) + HiC from HG04217	Hifiasm, Flye, and Quickmerge			no
PJL1[a]	HG03492	Punjabi, Lahore	2.9	203	pangenome contigs + HiC	trio-Hifiasm	YaHS	none	splitting fused chromosomes
BIB1[a]	HG03009	Bengali, Bangladesh	3.0	154					
ITU1[a]	HG04217	Telugu, UK	3.0	198					
GIH1[a]	NA20847	Gujrati, Houston, Texas	3.0	154					
Han1	HG00621	Southern Han Chinese	3.10	148	PacBio HiFi (39.45×), ONT (34.7×)	assembler: Hifiasm and Flye	MaSuRCA with T2T-CHM13	reference-guided gap filling using T2T-CHM13	yes
Ash1	HG002	Ashkenazi Jew	2.97		Illumina (71×), ONT (23×), and PacBio HiFi (29×)	assembler: MaSuRCA v3.3.4, Mummer4 for aligning to GRCh38	MaSuRCA with GRCh38	reference guided and incorporating 58.3 Mb from GRCh38	yes
PR1	HG01243	Puerto Rican	3.1		Illumina (59×), ONT (35×), PacBio HiFi (34×), and PacBio CLR (113×)	Hifiasm and Flye	MaSuRCA with T2T-CHM13	reference guided and incorporating 280 Mb from T2T-CHM13	yes

[a]Including Han1, Ash1, and PR1 from the literature.

contigs, which were then scaffolded into chromosomes using Hi-C reads, the authors' laboratory has successfully obtained chromosome-level assemblies of several strains of malaria vectors approaching the assembly quality of the fly genome, which is considered the gold standard.[9–12]

In 2023, the first draft of the human pangenome was published using data from emerging sequencing technologies generated as part of the International Genome Sample Resource (IGSR) samples.[13] This work capitalized on the ethnic diversity within the IGSR sample collection to achieve a phased assembly of 47 individuals using state-of-the-art sequencing technologies. PacBio HiFi data were generated at a coverage of approximately 40×, complemented by Illumina reads from both parents. Although many samples included Oxford Nanopore Technologies (ONT), Illumina, and Bionano reads publicly available through IGSR, these were not utilized in the report. The authors employed the trio-hifiasm tool, which utilizes short-read Illumina data from both parents to produce phased contigs from assemblies generated by PacBio HiFi data. These assemblies remain at the contig level and have not yet been scaffolded to achieve chromosome-level representations, hindering comparative genomics.

Between the world of karyotype disorders, small structural variants, and SNPs/SNVs, there are other types of large variations, including large deletions, copy-number variations, and inversions, that are challenging to discover among individuals without chromosome-level completed genomes. Inversions larger than 1 Mb are either uncommon or poorly characterized. Structural variants within one of the longest inversions, 8p23.1 of 4 Mb size have been associated with congenital heart disease and neurodevelopmental disorders.[14] However, 8p23.1 inversion is found in assemblies, such as hg38 and Han1,[15] and missing in T2T-CHM13v2.0, which was assembled from an individual of Northern European descent.[15] It has been reported that the presence of this inversion in hg38 could result from the mixture of bacterial artificial chromosomes from multiple individuals, suggesting that at least the genome of one of the individuals used to create hg38 must have carried this inversion. The prevalence of this and many other large inversions across diverse population remains unknown. Using 10x Genomics linked-read sequencing technologies, many known large inversions in humans have been validated in Centre d'Etude du Polymorphisme Humain trio genomes.[16]

Despite the large representation of individuals of South Asian ancestry in IGSR, a chromosome-level reference assembly from South Asia is still missing, which limits efforts to fully understand human genetic diversity worldwide. More recently, a review on haplotype reference panels highlighted the importance of regional structural variants for successful imputation.[17] Nearly 10 years after the results from the UK10K project were reported, which involved 10,000 individuals from the United Kingdom representing 0.014% of the UK population, findings from the GenomeIndia project with 10,000 genomes, representing variations from a fraction of the Indian population (0.00076%), have been reported.[18] However, a reference genome representing one-sixth of the world's population in South Asia remains unrepresented.

Here, we report a chromosome-level assembled genome of an individual of Indian descent from the state of Karnataka (KGP), not part of the IGSR[19] or Genome in a Bottle Consortium[20] sample resources, using a hybrid approach with minimal coverage of PacBio HiFi reads, along with the publicly available Hi-C data from an individual presumed to be genetically closest to the subject. We have also assembled the genomes of other individuals from the Indian diaspora using publicly assembled contigs, which have not yet been assembled into chromosomes using Hi-C data from the same individuals.

Materials and methods

Samples

Blood samples from several individuals were collected with informed consent, along with self-reported ancestry linking them to their respective ethnicity/population. The samples were labeled anonymously to maintain confidentiality after obtaining ethics clearance (NHM/SPM/4-PART FILE/2020-21). DNA from one of these samples was isolated and sent for sequencing.

Optimization of coverage requirement

In Escherichia coli *without Hi-C*

The individual genomes reported so far have used at least 30–40× coverage of PacBio HiFi data, along with Illumina and ONT. Our goal here was to determine the minimal required coverage for PacBio HiFi reads to obtain chromosome-level assemblies of individuals while keeping the cost low. Coverage is a normalized factor proportional to the size of the genome. For example, a 12× coverage of *E. coli* represents only 48 million bases of sequence data, whereas a 12× coverage of the human genome will represent 36,000 million bases. Irrespective of the genome size, 12× coverage means that each region of the genome is covered 12 times in the raw reads, providing significantly high overlaps necessary for the generation of long contigs. Therefore, validation of coverage in *E. coli* can apply to genomes of all sizes.

We used a publicly available dataset with 1,800× coverage of PacBio HiFi data (SRA: SRR11434954) generated from an *E. coli* strain, providing an opportunity to systematically test the coverage requirement independent of the genome size. We randomly extracted reads representing 1×, 2×, 3×, 4×, 8×, 12×, 16×, and 20× coverage using an option within the seqtk toolkit. The experiment was repeated five times for each coverage, and the average N50, L50, and assembled genome sizes of all five experiments across increasing coverage are shown in Figure 1. According to these, at 12× coverage (4,000 reads on the *x* axis), the entire genome is represented in contigs, as indicated by the additive genome size.

Minimum coverage of PacBio HiFi with Hi-C data in humans

PacBio HiFi reads for the PJL1 individual (HG03492, from Punjab, Lahore) were obtained from the Human Pangenome Reference Consortium (HPRC) Year 1 dataset. The reads were distributed across three ccs.bam files and were merged using Samtools merge

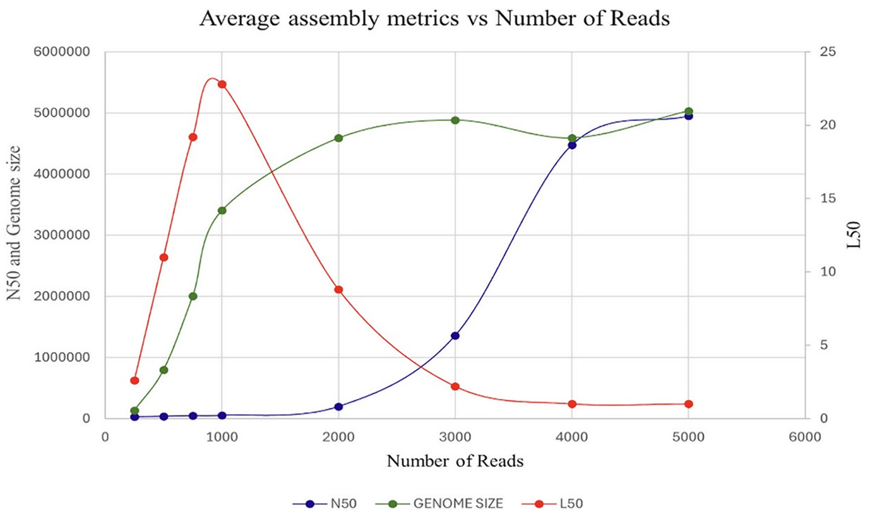

Figure 1. Assembly metrics using different coverages of PacBio HiFi reads
L50 (red), N50 (blue), and genome sizes (green) with the increasing number of reads on the *x* axis (coverage).

to generate a unified dataset. To support chromosome-scale scaffolding, raw Hi-C sequencing data for the same individual were retrieved from the HPRC's public repository (https://s3-us-west-2.amazonaws.com/human-pangenomics/index.html?prefix=working/HPRC_PLUS/HG03492/raw_data/). The merged ccs.bam file was converted to FASTQ format using the bam2fastq tool. Down-sampling to approximately 12× coverage was performed using the seqtk tool with the command given in the supplemental information.

De novo assembly was carried out using Hifiasm, an assembler optimized for PacBio HiFi reads.[21,22] Following assembly, the Burrows-Wheeler Aligner-MEM was used to align paired-end Illumina reads back to the Hifiasm-generated contigs. This alignment enabled the creation of a sorted bam file, which served as input for YaHS, a tool that leverages Hi-C contact maps to scaffold the contigs into chromosome-scale assemblies. This integration of read-pair information significantly enhanced the continuity and structural resolution of the assembly.

Assembly quality was evaluated using standard metrics. The resulting 12× scaffold-level assembly (PJL1.12×) achieved an N50 of 165 Mb and an L50 of 8, indicating a high degree of contiguity and completeness, consistent with near-chromosome-level assemblies. To evaluate PJL1.12× with that from 30× coverage (PJL1-v1), we compared the Benchmarking Universal Single-Copy Orthologs (BUSCO) score and found them both to be 98%.[23] Additionally, the number predicted genes in PJL1.12× using LiftOff is 63,277 compared to that from T2T, which is 61,312. Furthermore, we generated dot plots for all chromosomes using the virtual marker sets T2T1Mb and hg38100kb (see Figure S4) and plotted them against PJL1-v1. These plots illustrate the DNA-level linearity maintained between PJL1.12× and PJL1-v1, suggesting no spurious mis-assembles introduced at lower coverage.

Assembly strategy

PacBio tools were used to convert CCS subreads to HiFi reads by filtering for reads with quality >20 and subreads ≥3. HiFiAdapterFilt was used to remove PacBio bell adapter contamination. The resulting reads were assembled into contigs (KIn1hifi [KIn1, individual from the Indian subcontinent]) using Hifiasm with default parameters, and KIn1Flye was generated using Flye[24] with the option –pacbio-hifi. Contigs from both assemblies were merged using Quickmerge[25] to create KIn1merge with default parameters. KIn1merge and KIn1hifi contigs were separately scaffolded using YaHS[26] with 30× Hi-C data from ITU1 (HG04217, individual from Andhra Pradesh) from IGSR, using default parameters, resulting in assemblies KIn1.v1.5 and KIn1.v1, respectively. KIn1merge was additionally scaffolded using Hi-C from T2T-CHM13v2.0, producing KIn1.v1.8. YaHS separates contigs by adding only 100Ns between two adjacent contigs during scaffolding. Raw subreads were used to polish the assemblies by mapping onto the scaffolds. Scaffolding using Hi-C data resulted in chromosome-level assemblies.

The three scaffold-level assemblies were assessed for chromosome completeness and linearity by plotting each assembly with markers generated from the T2T-CHM13v2.0 assembly (see validation of assembly). For each chromosome, we selected the assembly that showed more DNA-level linearity and completeness to the respective chromosomes of T2T-CHM13v2.0 assembly. The selected chromosomes were checked for linearity and completion to produce KIn1.v2 without any manual correction, unlike KIn1.v1, which required manual adjustments.[27]

Assembly of other genomes

Contig-level assemblies for the individual PJL1 (HG03492) were obtained from the HPRC Year 1 dataset (https://s3-us-west-2.amazonaws.com/human-pangenomics/index.html?prefix=working/HPRC_PLUS/HG03492/assemblies/year1_f1_assembly/), which includes high-quality contig-level assemblies from 47 individuals representing diverse global populations. In parallel, raw Hi-C sequencing data corresponding to the PJL1 individual were obtained from the HPRC's publicly available repository (https://s3-us-west-2.amazonaws.com/human-pangenomics/index.html?prefix=working/HPRC_PLUS/HG03492/raw_data/hic/) to enable chromosome-scale scaffolding. The contig-level assemblies were provided in haplotype-resolved format, separated into maternal and paternal assemblies. Each haplotype-resolved assembly was indexed, and the raw Hi-C reads were aligned separately to the maternal and paternal assemblies in an end-to-end mode with default parameters. Duplicate reads from resulting bam files were removed using the MarkDuplicates module from Picard Tools (version 2.27.5). Scaffolding of the contig-level assemblies was performed using YaHS (version 1.2) with default parameters. The paternal scaffold-level assembly achieved an N50 of 146.7 Mb and an L50 of 8, while the maternal assembly achieved an N50 of 155.0 Mb and an L50 of 8.

A similar approach was repeated to assemble the genomes of BIB1 (Bengali from Bangaladesh [HG03009]), ITU1 (Indian Telugu from UK [HG04217]), GIH1 (Gujarati Indian from Houston [NA20847]), and PJL1 (Panjabi from Lahore [HG03492]) by downloading their respective contig-level assemblies and Hi-C data.

Validation of assembly

The strategy was to validate the linearity of the assembled genomes by mapping against uniformly spaced 2-kb virtual markers (1 Mb in Figure S1 and 100 kb in Figure S2) along each chromosome of a reference genome to establish DNA-level linearity. Usually, the linearity is validated by synteny of predicted genes in the assemblies as done by manuscripts reporting the assemblies of PR1,[5] Han1,[6] and Ash1.[7] Since only 1.5% of the genome codes for proteins, a DNA-level linearity provides better validation of genomes of individuals within the same species.

For the low-resolution 1-Mb markers (T2T1Mb), virtual markers of 2 kb length were generated every million bases on the T2T reference assembly using an in-house tool (see data and code availability). These markers were mapped using blastn on all assembled individual human genomes such as hg38, PR1, Ash1, Han1, BIB1, GIH1, PJL1, ITU1, GWD1 (individual from The Gambia [HG02587]), and KIn1.v2 to both validate KIn1.v2 assembly based on linearity of the markers and to identify major inversions and translocations in KIn1.v2 compared to other individuals. From the blast output, we filtered the hits that yielded the percentage identity close to 100%, as evidence of DNA-level identity of the genomes with the T2T reference. Also, we kept all the hits from the longest scaffolds, irrespective of the percentage identity, because these may represent regions of variation across individual genomes. The start coordinates of the markers against the scaffolds from the blast hits were then extracted (y axis) and plotted against the start of the marker coordinate on the reference (x axis) to create dot plots as shown in Figures S1–S3.

Testing the scalability of virtual marker-based comparative genomics

The virtual marker-based approach was tested by comparing the 69 different genomic assemblies of various strains of *Arabidopsis thaliana* from a reported study.[28] These assemblies were retrieved using the NCBI BioProject accession number PRJNA1033522. The accession GCF_000001735.4_TAIR10.1 from the NCBI database was used as reference for both alignment and marker generation. Virtual markers were generated from the reference genome at 100-kb intervals, each spanning 2 kb in length, across all 5 chromosomes of *A. thaliana*. Each of the 69 genome assemblies was aligned against the virtual markers using the blastn to detect reported inversions. Post-alignment results were filtered using awk to extract the marker start coordinate and the corresponding subject coordinates for each of the 69 genomes. For visualization, dot plots for each chromosome were generated using in-house Python code (see data and code availability), which utilizes the matplotlib library. In these dot plots, the x axis represents the marker start coordinates, while the y axis corresponds to the aligned subject start coordinates for blast hits from the respective genomes. This graphical representation enables the clear identification of reported chromosomal inversions across different strains of *A. thaliana*, demonstrating scalability (see Figure S3).

Annotation of KIn1.v2

Annotation is another way to validate assemblies. This step is critical for KIn1.v2 as lower coverage can potentially introduce more sequencing errors in the assembly. While the DNA-level linearity using virtual markers confirms the quality of assembly, it is not sensitive to detecting sequencing errors. The integrity of predicted genes is suggestive of the absence of sequencing errors propagated by lower coverage. The annotation of KIn1.v2 using the T2T reference was done using Liftoff,[29] which was developed to annotate the Han1 genome. LiftoffTools version 0.4.3, developed by the same group,[30] was used to obtain transcript-level comparisons between the KIn1.v2 and Han1. This tool determines the effects of any DNA differences on the protein sequence for protein-coding transcripts and categorizes them into 9 groups: synonymous, non-synonymous, in-frame deletion, in-frame insertion, start codon loss, 5′ truncation, 3′ truncation, frameshift, and stop codon gain. This tool also provides a synteny module, which compares the gene order in the reference annotation to the order in the target annotation. The genes present in both annotations are sorted first by the chromosome and then by the start coordinate in each annotation, thus providing a synteny plot. The various command lines can be found in the supplemental information.

Results

Assembly of KIn1.v2

DNA was extracted from the blood of an individual from Karnataka (KIn1) after obtaining ethics clearance (NHM/SPM/4-PART FILE/2020-21) and confirming that their self-reported ethnicity/population was consistent with their ancestry, similar to the approach used in the 1000 Genomes Project. Based on the minimum required coverage of PacBio as described in materials and methods, 12× coverage of PacBio HiFi reads was produced and assembled using both Hifiasm[31] and Flye[24] after filtering for bell adapters. The contigs from the Hifiasm assembly were scaffolded using publicly available Hi-C data from an individual (ITU1, HG04217), who is presumed to be genetically most closely related to the subject, using the tool YaHS[26] (Yet another Hi-C scaffolding tool), resulting in the assembly KIn1.v1.[27] The contigs from Hifiasm and Flye assemblies were then merged using Quickmerge[25] to produce contigs with higher N50 values. These merged contigs were also scaffolded using Hi-C reads from the ITU1. The resulting scaffold-level assembly, KIn1.v2, has an N50 of 141 Mb and an L50 of 9, which are close to the expected values of 147 Mb and 8, respectively, for the reference genome. As shown in Figure 2, the longest scaffolds, representing most chromosomes, cover over 95% of the respective chromosomes in the reference genome T2T-CHM13v2.0. Chromosomes 3–6 are longer in KIn1 than in T2T-CHM13v2.0, which falls within the expected range of variations in human chromosomes, especially in centromeric regions.[32]

Virtual DNA markers extracted from the T2T-CHM13v2.0 assembly (see materials and methods) were blasted against the scaffolds of KIn1.v2 to both assign chromosomes and authenticate the linearity of the DNA sequence within the assembled scaffolds. The coverage of the longest scaffolds representing chromosomes 9 and 16, known to have large centromeres, and a few acrocentric chromosomes, is considerably lower. This may be due to the lack of Hi-C reads bridging centromeric regions to euchromatic regions.

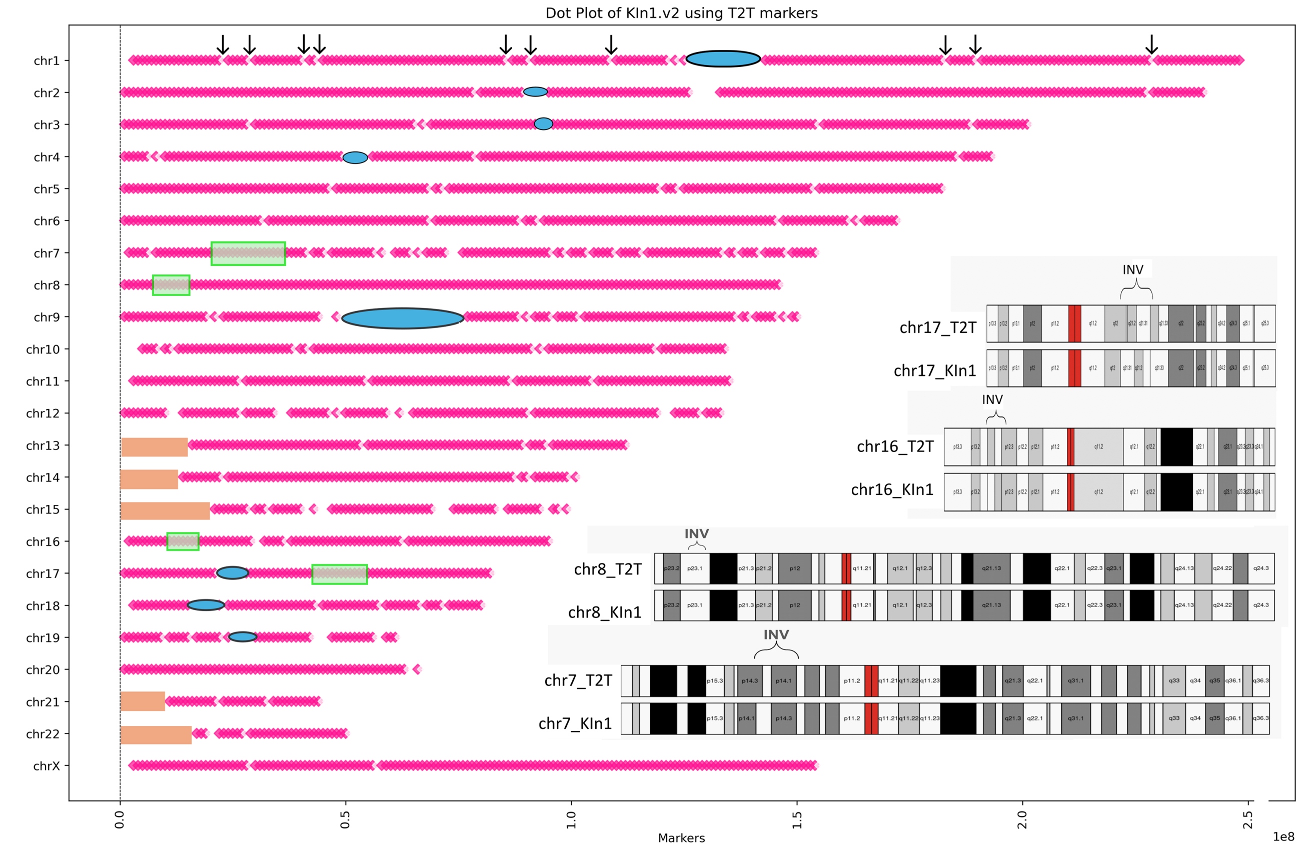

Figure 2. Graphical representation of KIn1 chromosomes
Pink diamonds along the chromosomes show 100% similarity on KIn1.v2 longest scaffolds with respect to virtual markers. Arrows pointing to the gaps represent markers with lower percentage identity; blue ellipses are centromeres; acrocentric regions are shown in orange rectangles; translucent green boxes are positions of inversions in KIn1.v2 (not to size) compared to T2T-CHM13v2.0. Inset shows the karyogram of chromosomes with inversion larger than 1 Mb drawn to size.

Chromosome-level assemblies of other individual genomes from the Indian diaspora

There are five distinct ethnicities, with hundreds of individuals from the Indian diaspora represented in the IGSR repository. Among these, contig level assemblies from 30× to 40× coverage of PacBio HiFi reads for a few individuals have been made publicly available as part of the pangenome project.[33] We have used these contig-level assemblies to build chromosome-level assemblies using the publicly available Hi-C data from the same individuals. These scaffold-level assemblies include the following:

(1) PJL1, a Punjabi from Lahore (HG03492) with an L50 of 6 and an N50 of 203 Mb
(2) BIB1, a Bengali from Bangladesh (HG03009) with an L50 of 7 and an N50 of 154 Mb
(3) GIH1, a Gujarati from Houston, Texas (NA02847) with an L50 of 8 and an N50 of 154 Mb
(4) ITU1, a Telugu-speaking community from the United Kingdom (HG04217) with an L50 of 6 and an N50 of 198 Mb
(5) GWD1, a Gambian from Africa (HG02587) with an L50 of 8 and an N50 of 156 Mb

The African genome GWD1 was included to represent an individual from the African continent for more comprehensive comparative genomics studies. Assemblies with an L50 less than 8 and an N50 larger than 148 Mb are considered chimeric chromosomes from assembly artifacts. These can be corrected manually for use in comparative genomics.

Validation of individual assemblies

The BUSCO scores for all genome assemblies reported here and published elsewhere are compared. The scores of PR1, Ash1, and Han1 are 98.4% similar to those of hg38 and T2T, perhaps because of reference-based gap filling. The BUSCO scores for assemblies of individual females are ITU1 98%, GIH1 97%, GWD1 98%, and KIn1 90.8%. For the two individual males, BIB1 and PJL1, the BUSCO scores of the assemblies using the phased contigs from paternal arm are 97% and 96%, respectively. As expected, the 262 genes missing in BUSCO evaluation of PJL1.v1 are from the X chromosome. Additionally. the lower BUSCO score for KIn1.v2 results from the use of Hi-C reads from other individuals as revealed by the lower mapping percentage of Hi-C reads from ITU1 on KIn1.v2 (80%) compared to the mapping percentage of Hi-C reads from PJL1 onto PJL.12× assemblies (90%).

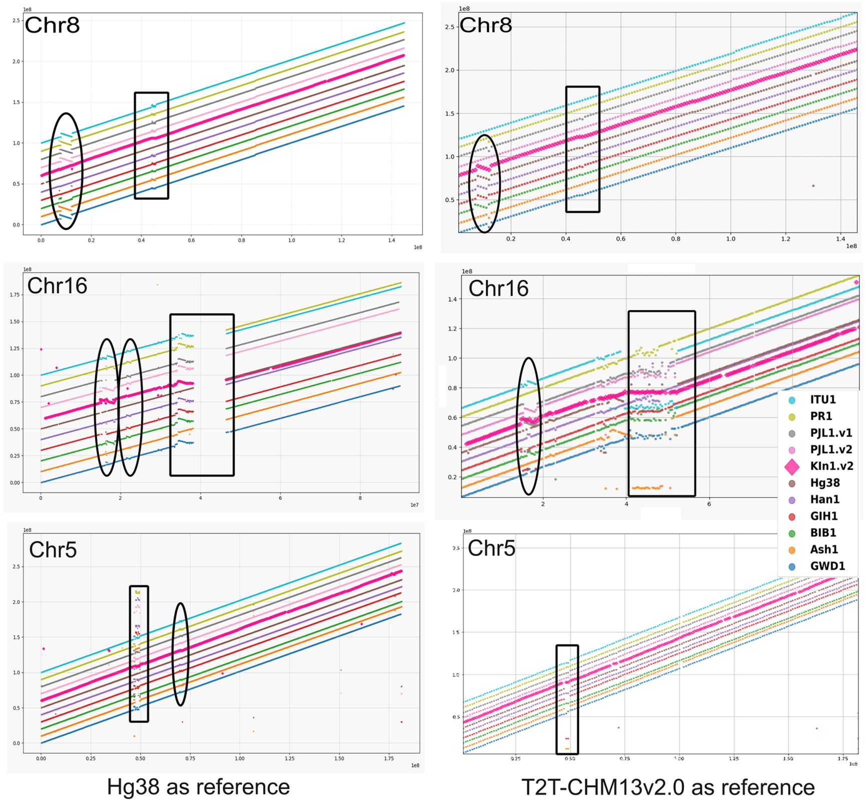

Figure 3. A comparative dot plot for strategically selected chromosomes from Ash1, PR1, Han1, hg38, PJL1.v1, PJL1.v2, BIB1, GIH1, ITU1, GWD1, and KIn1.v2 compared to hg38100kb and T2T1Mb

Centromeric regions are boxed, and inversion regions, common to more than one individual, are circled. To prevent genomes from overlapping on the diagonal, they are shifted on the y axis by 5 Mb or as needed with genomes of individuals arranged from bottom to top: GWD1, Ash1, BIB1, GIH1, Han1, hg38, KIn1.v2 (pink), PJL1.v2, PJL1.v1, PR1, and ITU1. Note that PJL1.v1 and PJL1.v2 are scaffolded using Hi-C from PJL1 and ITU1, respectively.

We have compared the linearity of individual genomes from diverse populations described in terms of language, ethnicity, and geography. These individuals include Puerto Rican (PR1), Ashkenazi Jew (Ash1), Southern Han Chinese (Han1), Northern European (CHM13), Punjabi (PJL1), Bangladeshi (BIB1), Telugu (ITU1), Gujarati (GIH1), Gambian (GWD1), and Karnataka (KIn1.v2). The comparison was performed using the virtual DNA markers extracted from the reference chromosomes of T2T-CHM13v2.0 as described in materials and methods. Figure 2 shows dot plots for selected chromosomes using virtual markers spaced 1 MB apart on T2T (T2T1Mb: right) and 100 kb on hg38 (hg38100kb: left) references. While T2T1Mb is relatively efficient in assigning scaffolds to chromosomes and validating linearity, it is not sensitive enough to detect inversions shorter than 2 Mb. However, the hg38100kb markers can detect inversions/translocations as short as 200–300 kb. The enlarged plots for all the chromosomes from T2T1Mb are shown in Figures S1A–S1W and selected plots from hg38100kb are shown in Figures S2A–S2E.

The pink diamonds in Figure 2, representing the KIn1.v2 assembly using T2T1Mb, indicate near linearity achieved within the euchromatic regions of scaffolds. It should be noted that, unlike the previously published genome assemblies of Ash1, Han1, and PR1, the KIn1.v2 is not a reference-based assembly and is scaffolded by Hi-C data, avoiding the need to utilize gap filling or manual corrections.

The linearity of the euchromatic regions of other assemblies compared in this report is evident from dots lining up along the diagonal in Figure 3. Since gaps in Ash1 and PR1 are filled with sequences from T2T-CHM13v2.0 and assemblies are manually corrected, it is not surprising that they are arranged along the diagonal. As shown by the black boxes in Figure 3, there are clusters of markers within centromeres that do not align linearly in most assembled genomes, suggesting considerable variations within this region across populations.[32] In the case of KIn1.v2, there is a shift in the dot plot near the centromere because the contigs representing centromeric regions in these chromosomes were not absorbed into the longest scaffolds, perhaps because Hi-C data from another individual was used. This effect is most dramatic in chromosomes 9 (Figure S1I) and 16 (Figure 3, center panel), which are known to have the longest centromeres. The gaps in the dot plots along the diagonal within euchromatic regions may represent population-specific variations.

Interrogation of large inversions

As listed in Table 1, we identified 4 inversions larger than 2 Mb in KIn1.v2 within the loci 8p23.1 and 16p12.3, which overlap 100% with known inversions. Additionally, we found 2 novel inversions, 17qKIn1 and 7pKIn1, with partial overlaps with known small inversions/microdeletions, 17q21.31 and 7p14.1, respectively. The second column in Table 2 provides the breakpoint coordinates using hg38100kb markers. Column 4 lists the number of genes within each inversion, while column 5 provides the names of genes within the respective known inversions that overlap with the inversions in KIn1.v2. For example, the PLEKHM1 gene is common to 17qKIn1 and one of the known inversions, 17q21.31. Similarly, the GLI3 gene is common to 7pKIn1 and one of the

Table 2. Large inversions in individuals compared to hg38 and T2T-CHM13v2.0 references

Large inversions	Breakpoint coordinates in hg38 (100 kb blocks)	Individuals with inversions compared to T2T	No. of genes within inversion loci	Reported genes within known inversions overlapping with KIn1 inversions	Genes associated with polymorphism within inversions
8p23.1	chr8:8200001-12100001	hg38, Han1, GIH1, BIB1, KIn1	49	BLK, NEIL2, MSRA, CTSB, FDFT1, MFHAS1, MTMR9, FAM167.A, PPP1R3B	BLK, NEIL2, MSRA, CTSB, FDFT1, MFHAS1, MTMR9, FAM167.A, PPP1R3B[29]
16p12.3	HsInv363	PJL1, KIn1, ITU1	21	NDE1, ABCC1, ABCC6, MYH11	(NDE1, MYH11, ABCC1, ABCC6)[30]
	HsInv368	PJL1, KIn1, ITU1, BIB1			
16p (proximal)	Chr16_KV880768v1_fix (chr16:21500001–224000001)	ITU1, PJL1, PR1, Han1, GIH1, BIB1, GWD1	12	METTL9 and OTOA (deafness related)	
5p13.2	chr5-GL339449v2_alt (chr5:70700001-70800001)	In all except hg38	none	none	none
17qKIn1 overlapping with 17q21.31	chr17:38300001-45400001	KIn1	205	PLEKHM1	MAPT, SPPL2C, CRHR1, PLEKHM1[31]
7pKIn1 overlapping with 7p14.1	chr7:35900001-43900001	KIn1	50	GLI3	GLI3[32]

known inversions, 7p14.1. Additionally, column 6 lists the genes within the respective inversions associated with polymorphism.[34–37]

8p23.1

The 8p23.1 polymorphic inversion is the largest reported in humans so far (4.5 Mb), which varies based on the copy-number variations near the breakpoints. The coordinates of this inversion in hg38100kb are chr8:8200001-12100001 (column 2 of Table 2). For clarity, we refer to the orientation of 8p23.1 in hg38 as 8p23.1std and that in T2T as 8p23.1inv. The circled regions in Figure 3, relative to hg38 (Figure 3, left) and T2T-CHM13 (Figure 3, right), show complementary status of this inversion across genomes of multiple individuals compared here. For example, the orientation highlighted in chromosome 8 in Figure 3 shows the presence of 8p23.1std in individual assemblies from Han1, BIB1, GIH1, ITU1, KIn1.v2, and hg38. In the genomes of T2T, Ash1, PR1, PJL1, and GWD1 assemblies, 8p23.1inv is present. According to one report, the frequency of 8p23.1inv is 59% among the Yoruba population of Nigeria (YRI), 20%–50% among Europeans, and 12%–27% among Asians.[34] The inversion status is reported to be heterozygous in Asian populations, including CHB and JPT (Japanese in Tokyo), and homozygous in CEU (Northern and Western European ancestry in Utah) and YRI populations.[36]

It is also reported that the 8p23.1 inversion is recurrent and may be under selection pressure[35]; it has arisen at least 15 times in human history. It is one of the most common inversions, with an allele frequency of 0.5, and therefore predicted to be heterozygous in 50% of all meiosis. To characterize the inversion genotype, we looked at the 16 SNPs reported in 8p23.1 inversion status using individuals from the Spanish population displaying homozygosity for inverted alleles (Table S1). In the study by Bosch et al., the authors report that a gene cluster (NEIL2, MSRA, CTSB, and BLK) within this locus is differentially expressed in an allele-specific fashion.[38]

Evidence for the three reported large inversions in chromosome 16p

Three large inversions are reported and validated on the p-arm of chromosome 16.[16] Of these, the two known distal inversions (HsInv0363 and HsInv0368) on the p-arm of chromosome 16 are also observed in Figure 3 (center), with breakpoint coordinates at chr16:14900001-18700 001 in hg38. Both these inversions are fused in KIn1.v2 and PJL1 and are split in ITU1. HsInv0368 is present in KIn1, ITU1, BIB1, and PJL1, suggesting the prevalence of HsInv0368 among South Asians, despite the allele frequency of HsInv0368 being as low as 0.08%, with a recurrence rate of 0.13%.[35] We also find evidence of the third reported and validated inversion on chromosome 16, with breakpoint coordinates between 21 and 22 Mb, present in individuals ITU1, BIB1, GWD1, PR1, PJL1, Han1, and GIH1. This inversion is absent in KIn1 and Ash1 compared to hg38.

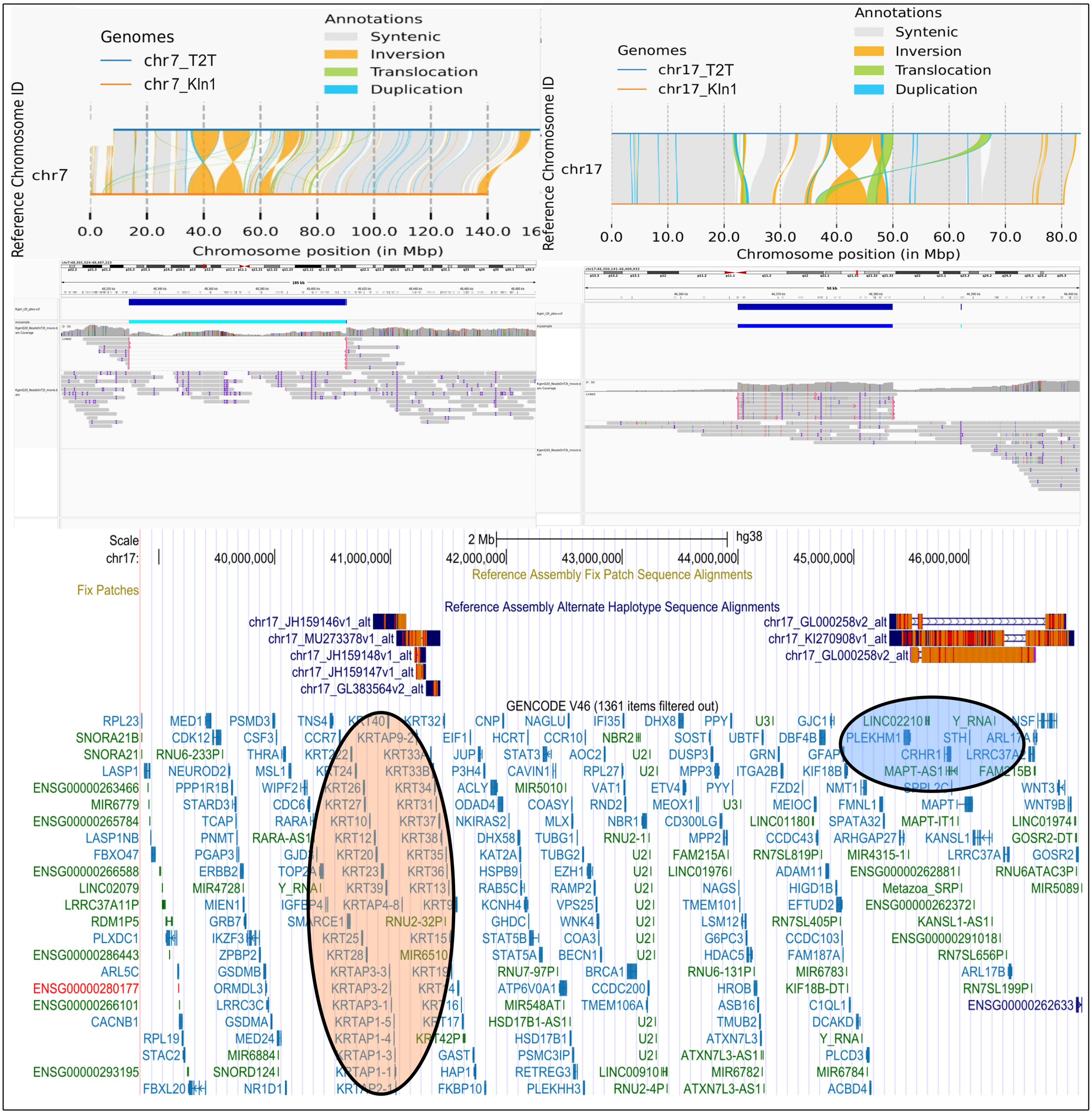

Figure 4. Large inversions in KIn1

Large inversions in chromosome 7 (top left) and chromosome 17 (top right) in KIn1.v2 against T2T-CHM13v2.0. (Center) Respective views from IGV of raw KIn1 PacBio reads mapped onto the respective regions of T2T-CHM13v2.0 reference. (Bottom) 17qKIn1 inversion locus in UCSC browser highlighting the genes within the distal (blue) and proximal (orange) breakpoints.

A large inversion in 17q unique to KIn1 (17qKIn1)

We have identified a large inversion in the q-arm of chromosome 17, with breakpoint coordinates of chr17:38300001-45400001 in hg38, which is unique to KIn1 and is referred to as 17qKIn1 (Figure 4, top right). Figure 4 (center right) shows the mapping of raw PacBio HiFi reads from KIn1 onto the T2T-CHM13v2.0, which validates the presence of 17qKIn1 inversion in KIn1 compared to the T2T reference. This region is gene rich, containing 205 genes, including PLEKHM1, which is known to be implicated in a microdeletion; this suggests that the distal breakpoint of 17qKIn1 overlaps with this microdeletion.[39] Furthermore, a 900-kb inversion polymorphism, 17q21.31, overlapping with the distal breakpoint of 17qKIn1, is found to occur at a frequency of 20% in the European Union (EU) and is practically absent in Africans and East Asians.[40] The screenshot of the 17qKIn1 locus is seen sandwiched between two haplotypes in the UCSC (University of California, Santa Cruz) browser, shows the MAPT gene, whose H2 orientation is known to occur only in the EU, just outside the distal breakpoint in Figure 4 (bottom). It is also shown that

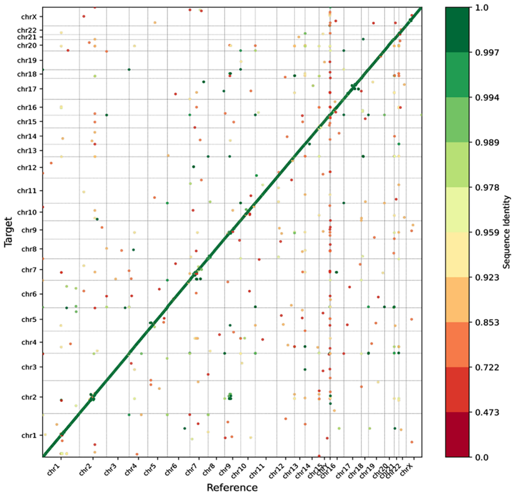

Assemblies	Han1	KIn1.v2
Number of annotated transcripts	181708	167024
Identical	14130	17261
Start-Lost	21	83
Start-Gained	31	39
Frameshift	143	404
Synonymous	2422	3501
Non-Synonymous	3200	4422
5' truncated	-	0
3' truncated	-	14
In-frame Deletion	92	223
In-frame Insertion	110	109

Figure 5. Protein-level synteny between KIn1.v2 and T2T-CHM13v2.0 assemblies

(Left) The synteny of genes from all chromosomes between T2T reference and KIn1.v2. (Right) Comparison of various gene characteristics between Han1 and KIn.v2 assemblies.

the inversion status of 17q21.31 affects the expression of specific genes within this locus.[41] The 17qKIn1 locus includes the BRCA1 gene, which is implicated in breast cancer. Interestingly, the 17q21.31 inversion, which overlaps with the distal breakpoint of 17qKIn1, has potentially been linked to cancer prognosis via methylation polymorphism.[42] The proximal breakpoint of 17qKIn1 contains the KRT gene cluster, offering a high level of redundancy to allow for functionally non-disruptive breakpoints.[43]

A large inversion unique to KIn1, 7pKIn1

A large inversion of 9 Mb in the chromosome 7 p-arm, with breakpoint coordinates of chr7:35900001-43900001 in hg38, is found to be unique to KIn1 (Figure 4, top left). Interrogation of the breakpoints for this inversion, by mapping raw PacBio reads from KIn1 against the T2T-CHM13v2.0 assembly, show very few reads spanning this region, thus validating the presence of this inversion in KIn1 and its absence in T2T (Figure 4, center left). Since these inversions are not present in the ITU1 assembly, whose Hi-C reads were used to scaffold KIn1.v2, we conclude that this inversion is indeed unique to KIn1. Interestingly, a microdeletion at the 7p14.1 locus, which is spanned by the 7pKIn1 inversion, is associated with Greig cephalopolysyndactyly.[44] There are 51 genes within the 7pKIn1 locus, including the GLI3 genes, which are implicated in Greig cephalopolysyndactyly.

Annotation of KIn1.v2 assembly

Figure 5 (left) compares synteny between predicted genes from KIn1.v2 with T2T reference, showing the linearity of genes in all chromosomes except in regions with inversions. We annotated and compared variants of KIn1.v2 genes against T2T using Liftoff[29] and LiftoffTools,[30] respectively. The table in Figure 4 provides the statistics in KIn1.v2 compared to those reported for the Han1 assembly mapped against T2T-CHM13v2.0. The number of instances of variations in KIn1.v2 relative to T2T-CHM13v2.0 for most categories listed in Figure 5 (right) is very close to that reported for the Han1 gapless assembly.[5]

Discussion

Assembling chromosome-level individual human genomes remains a challenge, with only four such assemblies reported so far from IGSR sample repository and/or primary cell lines. The goal of a *de novo* assembly of individual genomes is to identify large structural variants, such as inversions and translocations, which are difficult to pinpoint even when complete genomes from several individuals are available. However, this goal is difficult, if not impossible, to achieve when relying on reference-based assemblies. For example, during the reference-based assembly of PR1,[4] the orientations of some contigs/scaffolds were manually adjusted to conform to the T2T-CHM13v2.0 assembly. While the gap-filling step in hg38 was achieved meticulously over a decade, gap filling in individual genome assemblies using a reference genome is often merely cosmetic and essentially mixes the genomes of two individuals, defeating the purpose.

Here, we report a minimalist approach to obtain a chromosome-level genome assembly from the blood sample of an individual from southern India, offering one of the earliest individual genomes representing South Asia. The cost-cutting efforts attempted here, which are essential to scale individual genome efforts, include demonstrating the following:

(1) Use of a blood sample to generate an individual genome as opposed to primary cell lines as in the case of genomes of PR1, Ash1, PJL1, Han1, BIB1, ITU1, and GIH1
(2) Using PacBio HiFi data 10×–12× coverage without the need for other long-read technologies, achieving cost savings
(3) A *de novo* assembly pipeline that uses Hifiasm along with scaffolding by Hi-C data as a powerful approach in individual genome efforts without the use of reference-based assembly and manual correction
(4) A hybrid approach for scaffolding, using available Hi-C data derived from an individual presumed to be genetically most closely related to the subject; to this end, the authors' lab has experience in using

Hi-C data from different strains of *Anopheles stephensi* and the impact of this on large inversions unique to a strain[9–11]

(5) A scalable comparative genomics approach mapping virtual markers derived from reference genomes onto the assemblies to authenticate the DNA-level linearity of scaffolds

With a few exceptions, the longest scaffolds in KIn1.v2 covered nearly 95% of the chromosomes, providing an L50 of 9 and an N50 of 141 Mb, statistics that are very close to the lowest and highest attainable values of 8 and 147 Mb for the human genome with 23 chromosomes and slightly lower than those assembled using greater than 30× coverage. Considering that the final assembly contains only ~0.7 million Ns covering less than 0.03% of the genome, the contigs offer a good breadth of coverage across the entire genome, similar to the case of *E. coli* assembly at 12× coverage (see materials and methods). Most of the unplaced contigs in the longest scaffolds are from centromeric regions, likely due to a lack of Hi-C read pairs connecting the centromere to euchromatic regions. The BUSCO score on all scaffolds after assembly was 94.7%, suggesting completeness of the assembly, whereas a BUSCO score of 91% for only the longest scaffolds representing chromosomes in KIn1.v2 suggests 3.7% of genes in unplaced scaffolds, perhaps from using Hi-C data from another individual (ITU1). Even a 91% BUSCO score in *de novo* assemblies can be considered complete based on the benchmarking reported for BUSCO, where for *Caenorhabditis elegans* it is as low as 90%.[23]

There is a need to find a balance between the minimum coverage of PacBio HiFi long reads with maximum coverage of Hi-C reads, to manage the cost consideration in individual genome assembly efforts. The idea is to generate high-quality assemblies from data attainable from one SMRT cell for humans. We believe that the cost of generating high coverage of Hi-C reads for individuals can be mitigated by collating all publicly available Hi-C read pairs from diverse individuals from the IGSR sample repository to create a pan-Hi-C reads dataset for humans. As the cost of long-read single-molecule sequencing drops, the advances made here are likely to accelerate individual genome efforts worldwide.

For comparative genomics, we mapped virtual markers (hg38100kb and T2T1Mb) from two reference genomes, hg38 and T2T, onto the other assembled scaffolds/genomes to create dot plots, as shown in Figure 3. This approach also scales to compare a large number of genomes from any species. We validated scalability by reproducing the reported inversions among the 69 strains of *A. thaliana* shown in Figure S3. Our comparative genomics approach is further validated by confirming the well-known 8p23.1std inversion locus, reported in Han1[6] and hg38,[15] which is shared by the genomes of individuals from South Asians (India), including ITU1, GIH1, BIB1, and KIn1. The inverted form of 8p23.1 is present in CHM13, Ash1, PR1, and PJL1, suggesting that 8p23.1std is a founder effect from migration into South Asia. Interestingly, 8p23.1 inversion is known to be recurrent, with an allele frequency of 50%.[35]

We find evidence of three reported inversions near chromosome 16p12.3, including HsInv0363, HsInv0368, and an ~900-kb proximal inversion from the other two. As shown in Figure 3, HsInv0368 is shared by BIB1, ITU1, PJL1, and KIn1, but it is absent in hg38, T2T, PR1, Ash1, Han1, and GIH1. Interestingly, HsInv0363 is also present in KIn1.v2, ITU1, and PJL1. The reported low frequency of 16p12.3 across individuals may result from the underrepresentation of South Asians in the studies reporting the frequency.[36]

The KIn1.v2 assembly has two large unique inversions, 7pKIn1 and 17qKIn1. The lack of these inversions in the assembled genome of ITU1 (HG04217), whose Hi-C data were used in scaffolding KIn1.v2, indicates that these inversions are either real or artifacts from using Hi-C reads from ITU1. We validated these inversions by observing significant gaps when mapping raw PacBio HiFi reads from KIn1 onto the T2T-CHM13v2.0 assembly (Figure 4, center). Based on our experience in scaffolding the genome of a malaria vector strain using Hi-C data from divergent strains with a given inversion genotype,[9] we conclude that the chromosome 7 inversion in KIn1 may be heterozygous. The presence of KIn1.v2-specific inversions raises the question of whether large inversions can be individual-specific in healthy humans. In support of this, individual-specific large inversions are found in various strains of *A. thaliana*, as shown in Figure S3.[28]

We believe that KIn1.v2 represents one of the earliest chromosome-level assemblies of an individual of Indian origin, providing a valuable reference genome for individuals from South Asia, a region that is not represented in the 1000 Genomes Consortium.[19] This effort should be viewed in the context of language diversity in India, which includes 24 major languages. Despite good faith efforts by the 1000 Genomes Consortium to include individuals from five language groups in India while creating the human variants database, the extent of India's genetic diversity remains underexplored. While the GenomeIndia project has begun to reveal the breadth of genetic variation, a reference genome representing South Asia is still lacking. The KIn1.v2 assembly, along with other individual genomes (BIB1, GIH1, PJL1, and ITU1) reported here, will serve as references representing diverse ethnicities from the east, west, north, and south, thereby bringing India onto a level playing field in advancing genomic research and beyond.

Data and code availability

The KIn1.v1 and KIn1.v2 assemblies can be found under the accession PRJNA1138089 from NCBI and/or by clicking here (https://doi.org/10.6084/m9.figshare.30092122.v1). Hi-C data

used for scaffolding both versions of KIn1 can be downloaded from HG04217 under IGSR. Raw PacBio HiFi reads will be made available under the same project ID upon request. We have provided fasta files of markers and code to produce dot plots at https://github.com/apoo96-g/Dot-Plot/. Also, the supplemental information is available on GitHub along with the code so that updates to the dot plots from future versions of the code can be accessed dynamically.

Acknowledgments

PacBio HiFi reads were generated using services from Nucleome Informatics. The authors thank Government of Karnataka for funding for sequencing and data analysis personnel via a BioIT grant and computing infrastructure via the Department of Information Technology, Biotechnology and Science and Technology. The authors also acknowledge using computing resources obtained as part of DBT Builder Sanction no. BT/INF/22/SP45402/2022 dated March 8, 2022, CCB Sanction no. BT/PR40212/BTIS/137/40/2022 dated December 19, 2022, and DST-FIST Sanction no. SR/FST/LSI-536/2012.

Author contributions

A.G., assembly of KIn1.v2, variant analysis, and dot plots; A.M., assembly of KIn1.v1; Y.C., annotation, T2T markers, and karyogram; M.G., scaffolding KIn1.v2 using T2T Hi-C data; P.M., optimizing required read depth and assembly of BIB1, GWD1, and GIH1; A.S., assembly of PJL1 and ITU1; M.K., scalability of genome comparison; F.R., sample preparation; S.S., overseeing the bioinformatics work and writing of the manuscript; B.C., conceptualization of the project, obtaining ethics clearance, and writing of the manuscript.

Declaration of interests

The authors declare no competing interests.

Supplemental information

Supplemental information can be found online at https://doi.org/10.1016/j.xhgg.2025.100516.

Web resources

BIB1: HG03009, contig file, https://ftp.ncbi.nlm.nih.gov/genomes/all/GCA/040/450/255/GCA_040450255.1_ASM4045025v1/GCA_040450255.1_ASM4045025v1_genomic.fna.gz

BIB1: HG03009, Hi-C data, ftp://ftp.sra.ebi.ac.uk/vol1/fastq/ERR450/005/ERR4501115/ERR4501115.fastq.gz; ftp://ftp.sra.ebi.ac.uk/vol1/fastq/ERR450/006/ERR4506846/ERR4506846.fastq.gz

GIH1: NA20847, contig file, https://ftp.ncbi.nlm.nih.gov/genomes/all/GCA/040/448/775/GCA_040448775.1_ASM4044877v1/GCA_040448775.1_ASM4044877v1_genomic.fna.gz

GIH1: NA20847, Hi-C data: ftp://ftp.sra.ebi.ac.uk/vol1/fastq/ERR450/002/ERR4501112/ERR4501112.fastq.gz; ftp://ftp.sra.ebi.ac.uk/vol1/fastq/ERR450/001/ERR4501161/ERR4501161.fastq.gz

GWD1: HG02587, contig file, https://ftp.ncbi.nlm.nih.gov/genomes/all/GCA/040/450/255/GCA_040450255.1_ASM4045025v1/GCA_040450255.1_ASM4045025v1_genomic.fna.gz

GWD1: HG02587, Hi-C reads files, ftp://ftp.sra.ebi.ac.uk/vol1/vol1/fastq/ERR450/002/ERR4501102/ERR4501102.fastq.gz; ftp://ftp.sra.ebi.ac.uk/vol1/vol1/fastq/ERR447/001/ERR4472511/ERR4472511.fastq.gz

ITU1: HG04217, contig file, https://ftp.ncbi.nlm.nih.gov/genomes/all/GCA/040/449/615/GCA_040449615.1_ASM4044961v1/GCA_040449615.1_ASM4044961v1_genomic.fna.gz

ITU1: HG04217, Hi-C data: https://ftp.1000genomes.ebi.ac.uk/vol1/ftp/data_collections/HGSVC3/working/2021_Hi-C_JAX/HG04217_GT21-17614_AAGCATCG-CTGGAGTA_S87_L003_R1_001.fastq.gz; https://ftp.1000genomes.ebi.ac.uk/vol1/ftp/data_collections/HGSVC3/working/2021_Hi-C_JAX/HG04217_GT21-17614_AAGCATCG-CTGGAGTA_S87_L003_R2_001.fastq.gz

Received: July 2, 2025
Accepted: September 11, 2025

References

1. Lander, E.S., Linton, L.M., Birren, B., Nusbaum, C., Zody, M.C., Baldwin, J., Devon, K., Dewar, K., Doyle, M., FitzHugh, W., et al. (2001). Initial sequencing and analysis of the human genome. Nature *409*, 860–921.
2. Venter, J.C., Adams, M.D., Myers, E.W., Li, P.W., Mural, R.J., Sutton, G.G., Smith, H.O., Yandell, M., Evans, C.A., Holt, R.A., et al. (2001). The sequence of the human genome. Science *291*, 1304–1351.
3. Schneider, V.A., Graves-Lindsay, T., Howe, K., Bouk, N., Chen, H.C., Kitts, P.A., Murphy, T.D., Pruitt, K.D., Thibaud-Nissen, F., Albracht, D., et al. (2017). Evaluation of GRCh38 and *de novo* haploid genome assemblies demonstrates the enduring quality of the reference assembly. Genome Res. *27*, 849–864.
4. Nurk, S., Koren, S., Rhie, A., Rautiainen, M., Bzikadze, A.V., Mikheenko, A., Vollger, M.R., Altemose, N., Uralsky, L., Gershman, A., et al. (2022). The complete sequence of a human genome. Science *376*, 44–53.
5. Zimin, A.V., Shumate, A., Shinder, I., Heinz, J., Puiu, D., Pertea, M., and Salzberg, S.L. (2022). A reference-quality, fully annotated genome from a Puerto Rican individual. Genetics *220*, iyab227.
6. Chao, K.-H., Zimin, A.V., Pertea, M., and Salzberg, S.L. (2023). The first gapless, reference-quality, fully annotated genome from a Southern Han Chinese individual. G3 (Bethesda) *13*, jkac321.
7. Shumate, A., Zimin, A.V., Sherman, R.M., Puiu, D., Wagner, J.M., Olson, N.D., Pertea, M., Salit, M.L., Zook, J.M., and Salzberg, S.L. (2020). Assembly and annotation of an Ashkenazi human reference genome. Genome Biol. *21*, 129.
8. Garg, S., Fungtammasan, A., Carroll, A., Chou, M., Schmitt, A., Zhou, X., Mac, S., Peluso, P., Hatas, E., Ghurye, J., et al. (2021). Chromosome-scale, haplotype-resolved assembly of human genomes. Nat. Biotechnol. *39*, 309–312.
9. Chida, A.R., Ravi, S., Jayaprasad, S., Paul, K., Saha, J., Suresh, C., Whadgar, S., Kumar, N., Rao K, R., Ghosh, C., et al. (2020). A Near-Chromosome Level Genome Assembly of Anopheles stephensi. Front. Genet. *11*, 565626.
10. Thakare, A., Ghosh, C., Alalamath, T., Kumar, N., Narang, H., Whadgar, S., Paul, K., Shrotri, S., Kumar, S., Soumya, M., et al. (2022). The genome trilogy of Anopheles stephensi, an urban malaria vector, reveals structure of a locus associated with

adaptation to environmental heterogeneity. Sci. Rep. *12*, 3610.
11. Srinivasan, S., Ghosh, C., Das, S., Thakare, A., Singh, S., Ganesh, A., Mahawar, H., Jaisimha, A., Krishna, M., Chattopadhyay, A., et al. (2022). Identification of a TNF-TNFR-like system in malaria vectors (Anopheles stephensi) likely to influence Plasmodium resistance. Sci. Rep. *12*, 19079.
12. Chakraborty, M., Ramaiah, A., Adolfi, A., Halas, P., Kaduskar, B., Ngo, L.T., Jayaprasad, S., Paul, K., Whadgar, S., Srinivasan, S., et al. (2021). Hidden genomic features of an invasive malaria vector, Anopheles stephensi, revealed by a chromosome-level genome assembly. BMC Biol. *19*, 28.
13. Liao, W.W., Asri, M., Ebler, J., Doerr, D., Haukness, M., Hickey, G., Lu, S., Lucas, J.K., Monlong, J., Abel, H.J., and Buonaiuto, S. (2023). A draft human pangenome reference. Nature *617*, 312–324. https://pubmed.ncbi.nlm.nih.gov/37165242/.
14. Mohajeri, K., Cantsilieris, S., Huddleston, J., Nelson, B.J., Coe, B.P., Campbell, C.D., Baker, C., Harshman, L., Munson, K.M., Kronenberg, Z.N., et al. (2016). Interchromosomal core duplicons drive both evolutionary instability and disease susceptibility of the Chromosome 8p23.1 region. Genome Res. *26*, 1453–1467.
15. Yang, X., Wang, X., Zou, Y., Zhang, S., Xia, M., Fu, L., Vollger, M.R., Chen, N.C., Taylor, D.J., Harvey, W.T., et al. (2023). Characterization of large-scale genomic differences in the first complete human genome. Genome Biol. *24*, 157.
16. Eslami Rasekh, M., Chiatante, G., Miroballo, M., Tang, J., Ventura, M., Amemiya, C.T., Eichler, E.E., Antonacci, F., and Alkan, C. (2017). Discovery of large genomic inversions using long range information. BMC Genom. *18*, 65.
17. Yang, Q., Sun, Y., Duan, S., Nie, S., Liu, C., Deng, H., Wang, M., and He, G. (2025). High-quality Population-specific Haplotype-resolved Reference Panel in the Genomic and Pangenomic Eras. Genom. Proteom. Bioinform. *10*, qzaf022. https://doi.org/10.1093/gpbjnl/qzaf022.
18. Bhattacharyya, C., Subramanian, K., Uppili, B., Biswas, N.K., Ramdas, S., Tallapaka, K.B., Arvind, P., Rupanagudi, K.V., Maitra, A., Nagabandi, T., et al. (2025). Mapping genetic diversity with the GenomeIndia project. Nat. Genet. *57*, 767–773.
19. Church, G.M. (2005). The personal genome project. Mol. Syst. Biol. *1*, 2005.0030.
20. Zook, J.M., Catoe, D., McDaniel, J., Vang, L., Spies, N., Sidow, A., Weng, Z., Liu, Y., Mason, C.E., Alexander, N., et al. (2016). Extensive sequencing of seven human genomes to characterize benchmark reference materials. Sci. Data *3*, 160025.
21. Yu, W., Luo, H., Yang, J., Zhang, S., Jiang, H., Zhao, X., Hui, X., Sun, D., Li, L., Wei, X.Q., et al. (2024). Comprehensive assessment of 11 *de novo* HiFi assemblers on complex eukaryotic genomes and metagenomes. Genome Res. *34*, 326–340.
22. Cosma, B.-M., Shirali Hossein Zade, R., Jordan, E.N., van Lent, P., Peng, C., Pillay, S., and Abeel, T. (2023). Evaluating long-read *de novo* assembly tools for eukaryotic genomes: insights and considerations. GigaScience *12*, giad100.
23. Simão, F.A., Waterhouse, R.M., Ioannidis, P., Kriventseva, E. V., and Zdobnov, E.M. (2015). BUSCO: assessing genome assembly and annotation completeness with single-copy orthologs. Bioinformatics *31*, 3210–3212.
24. Kolmogorov, M., Yuan, J., Lin, Y., and Pevzner, P.A. (2019). Assembly of long, error-prone reads using repeat graphs. Nat. Biotechnol. *37*, 540–546.
25. Solares, E.A., Chakraborty, M., Miller, D.E., Kalsow, S., Hall, K., Perera, A.G., Emerson, J.J., and Hawley, R.S. (2018). Rapid Low-Cost Assembly of the Drosophila melanogaster Reference Genome Using Low-Coverage, Long-Read Sequencing. G3 (Bethesda) *8*, 3143–3154.
26. Zhou, C., McCarthy, S.A., and Durbin, R. (2023). YaHS: yet another Hi-C scaffolding tool. Bioinformatics *39*, btac808.
27. Mhatre, A., Chindarkar, Y., Ganesh, A., Thakare, A., Mishra, P., Ravindran, F., Sharma, A., Srinivasan, S., and Choudhary, B. (2024). The first assembled chromosome-level genome of an individual from Karnataka, India. Preprint at bioRxiv. https://doi.org/10.1101/2024.08.18.608448.
28. Lian, Q., Huettel, B., Walkemeier, B., Mayjonade, B., Lopez-Roques, C., Gil, L., Roux, F., Schneeberger, K., and Mercier, R. (2024). A pan-genome of 69 Arabidopsis thaliana accessions reveals a conserved genome structure throughout the global species range. Nat. Genet. *56*, 982–991.
29. Shumate, A., and Salzberg, S.L. (2021). Liftoff: accurate mapping of gene annotations. Bioinformatics *37*, 1639–1643.
30. Shumate, A., and Salzberg, S. (2022). LiftoffTools: a toolkit for comparing gene annotations mapped between genome assemblies. F1000Res. *11*, 1230.
31. Cheng, H., Concepcion, G.T., Feng, X., Zhang, H., and Li, H. (2021). Haplotype-resolved *de novo* assembly using phased assembly graphs with hifiasm. Nat. Methods *18*, 170–175.
32. Logsdon, G.A., Rozanski, A.N., Ryabov, F., Potapova, T., Shepelev, V.A., Mao, Y., Rautiainen, M., Koren, S., Nurk, S., Porubsky, D., et al. (2023). The variation and evolution of complete human centromeres. Preprint at bioRxiv. https://doi.org/10.1101/2023.05.30.542849.
33. Wang, T., Antonacci-Fulton, L., Howe, K., Lawson, H.A., Lucas, J.K., Phillippy, A.M., Popejoy, A.B., Asri, M., Carson, C., Chaisson, M.J., et al. (2022). The Human Pangenome Project: a global resource to map genomic diversity. Nature *604*, 437–446.
34. Salm, M.P.A., Horswell, S.D., Hutchison, C.E., Speedy, H.E., Yang, X., Liang, L., Schadt, E.E., Cookson, W.O., Wierzbicki, A.S., Naoumova, R.P., and Shoulders, C.C. (2012). The origin, global distribution, and functional impact of the human 8p23 inversion polymorphism. Genome Res. *22*, 1144–1153.
35. Porubsky, D., Höps, W., Ashraf, H., Hsieh, P., Rodriguez-Martin, B., Yilmaz, F., Ebler, J., Hallast, P., Maria Maggiolini, F.A., Harvey, W.T., et al. (2022). Recurrent inversion polymorphisms in humans associate with genetic instability and genomic disorders. Cell *185*, 1986–2005.e26.
36. Antonacci, F., Kidd, J.M., Marques-Bonet, T., Ventura, M., Siswara, P., Jiang, Z., and Eichler, E.E. (2009). Characterization of six human disease-associated inversion polymorphisms. Hum. Mol. Genet. *18*, 2555–2566.
37. Shih, B., Tassabehji, M., Watson, J.S., and Bayat, A. (2012). DNA copy number variations at chromosome 7p14.1 and chromosome 14q11.2 are associated with dupuytren's disease: potential role for MMP and Wnt signaling pathway. Plast. Reconstr. Surg. *129*, 921–932.
38. Bosch, N., Morell, M., Ponsa, I., Mercader, J.M., Armengol, L., and Estivill, X. (2009). Nucleotide, cytogenetic and expression impact of the human chromosome 8p23.1 inversion polymorphism. PLoS One *4*, e8269.
39. Koolen, D.A., Vissers, L.E.L.M., Pfundt, R., de Leeuw, N., Knight, S.J.L., Regan, R., Kooy, R.F., Reyniers, E., Romano, C., Fichera, M., et al. (2006). A new chromosome 17q21.31

microdeletion syndrome associated with a common inversion polymorphism. Nat. Genet. *38*, 999–1001.

40. Stefansson, H., Helgason, A., Thorleifsson, G., Steinthorsdottir, V., Masson, G., Barnard, J., Baker, A., Jonasdottir, A., Ingason, A., Gudnadottir, V.G., et al. (2005). A common inversion under selection in Europeans. Nat. Genet. *37*, 129–137.
41. de Jong, S., Chepelev, I., Janson, E., Strengman, E., van den Berg, L.H., Veldink, J.H., and Ophoff, R.A. (2012). Common inversion polymorphism at 17q21.31 affects expression of multiple genes in tissue-specific manner. BMC Genom. *13*, 458.
42. Ruiz-Arenas, C., Cáceres, A., Moreno, V., and González, J.R. (2019). Common polymorphic inversions at 17q21.31 and 8p23.1 associate with cancer prognosis. Hum. Genomics *13*, 57.
43. Ho, M., Thompson, B., Fisk, J.N., Nebert, D.W., Bruford, E.A., Vasiliou, V., and Bunick, C.G. (2022). Update of the keratin gene family: evolution, tissue-specific expression patterns, and relevance to clinical disorders. Hum. Genomics *16*, 1.
44. Montoro Cremades, D., Manchón Trives, I., Botella López, V., Alcaraz Más, L., García Martínez, M.R., and Galán Sánchez, F. (2011). 7p14.1 microdeletion and Greig cephalopolysyndactyly syndrome. An. Pediatr. *74*, 266–269.

HGGA, Volume 7

Supplemental information

The Karnataka Individual Genome Project expands the human reference landscape to include South Asia

Apoorva Ganesh, Anisha Mhatre, Yash Chindarkar, Moushmi Goswami, Prakruti Mishra, Aditya Sharma, Manjushri Kalpande, Febina Ravindran, Subhashini Srinivasan, and Bibha Choudhary

Table of Contents

Supplementary Figure S1 (A-W): shows comparative dot plot of individuals' chromosomes from several genomes against virtual markers every 1Mb from T2T assembly. 13

Supplementary Figure S2 (A-E): shows comparative dot plot of individuals' chromosomes from several genomes against virtual markers every 100kb from hg38 assembly. 16

Supplementary Figure S3: Comparison of chromosome 3 and 5 of 69 accession of Arabidopsis (PMID: 38605175) compared to the virtual markers from the reference Arabidopsis genome. 17

Supplementary Figure S4: Comparison of PJL1.12x against PJL1.v1: Bench marking results for 12x coverage by down-sampled assembly from 30x raw reads of PJL1........ 29

Supplementary Table S1: 30

Supplementary Methods: 30

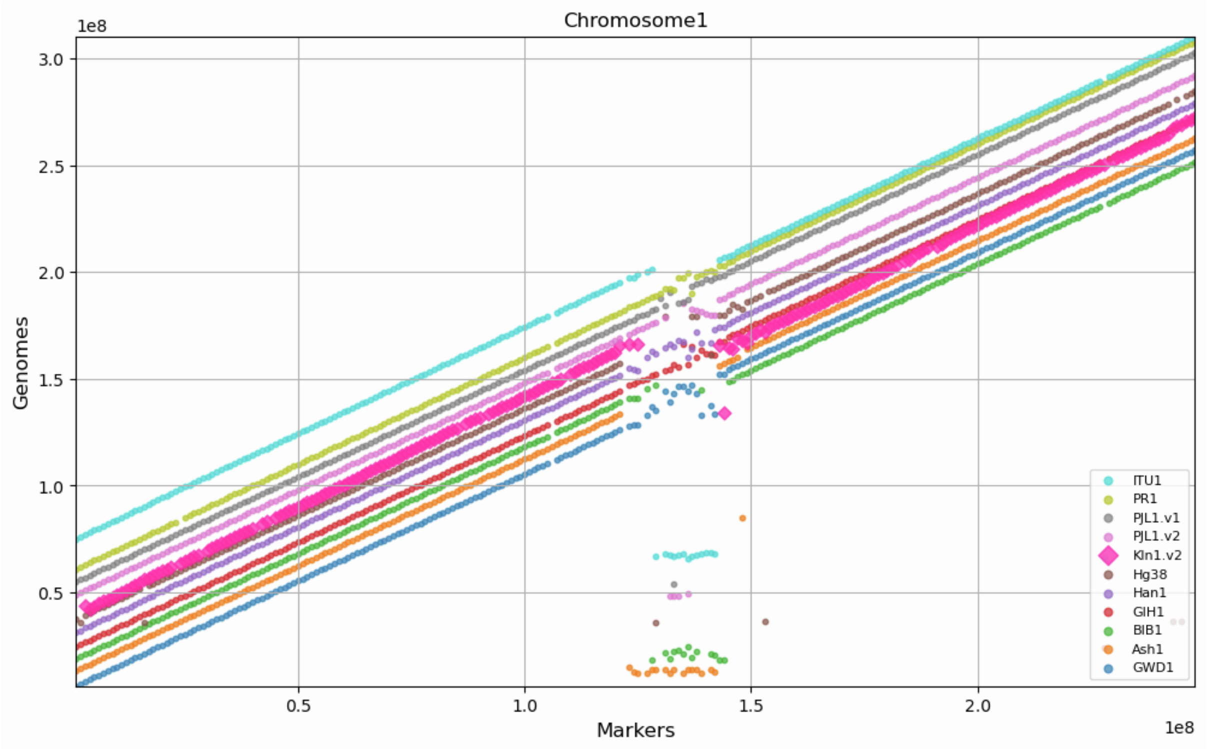
Chromosome1
1e8
Genomes
Markers
ITU1
PR1
PJL1.v1
PJL1.v2
Kln1.v2
Hg38
Han1
GIH1
BIB1
Ash1
GWD1

(A)

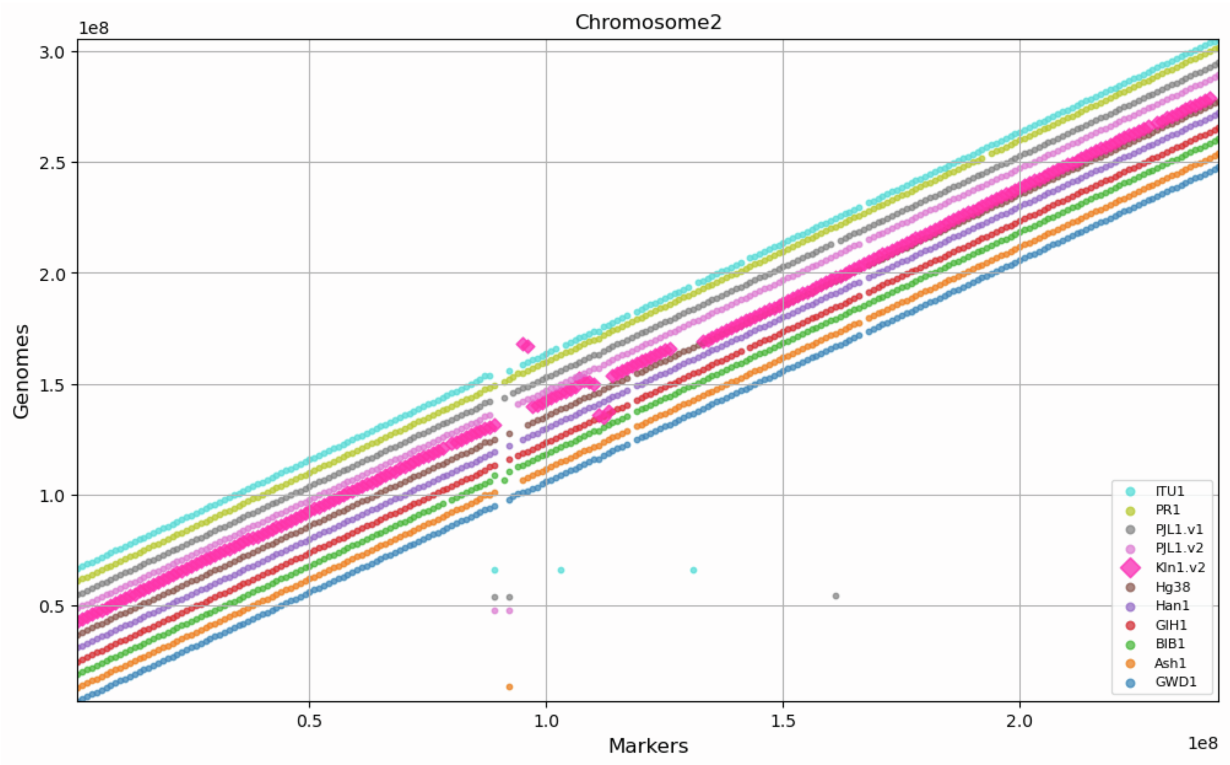
Chromosome2
1e8
Genomes
Markers
ITU1
PR1
PJL1.v1
PJL1.v2
Kln1.v2
Hg38
Han1
GIH1
BIB1
Ash1
GWD1

(B)

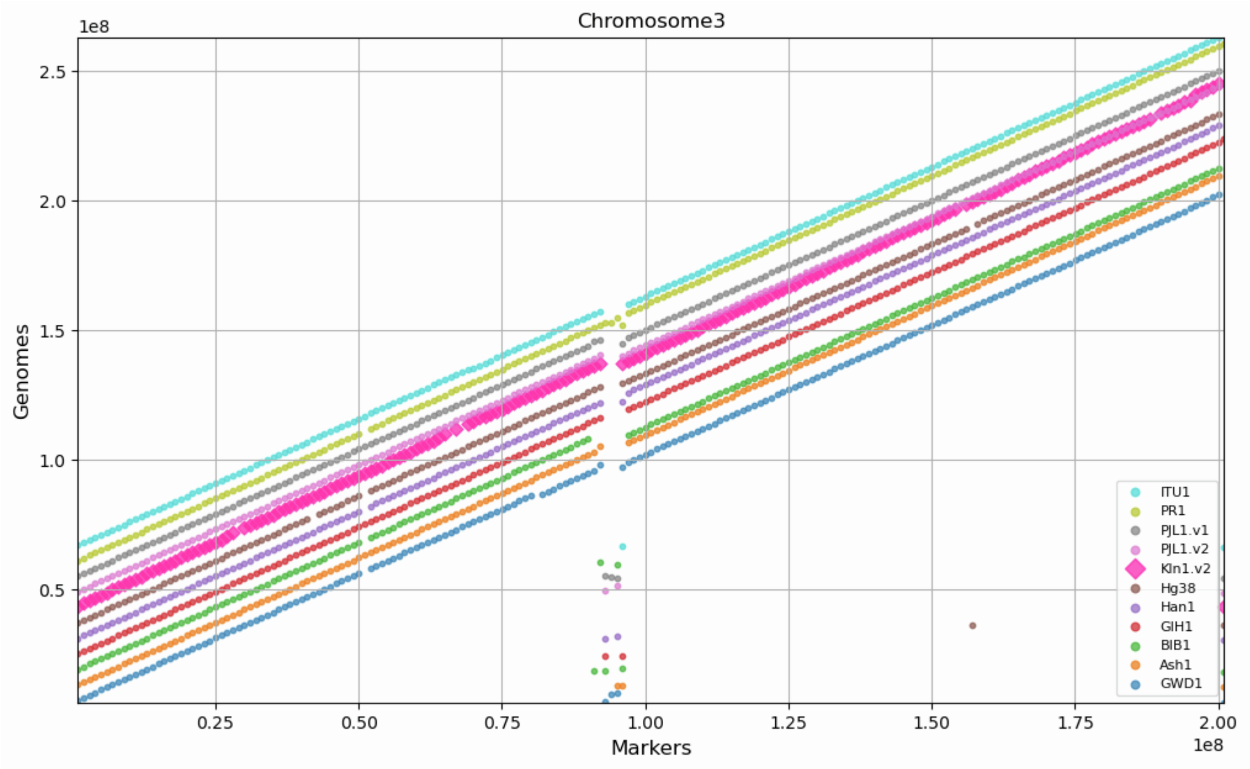
Chromosome3
1e8
2.5
2.0
1.5
1.0
0.5
Genomes
0.25
0.50
0.75
1.00
1.25
1.50
1.75
2.00
Markers
1e8
ITU1
PR1
PJL1.v1
PJL1.v2
Kln1.v2
Hg38
Han1
GIH1
BIB1
Ash1
GWD1

(C)

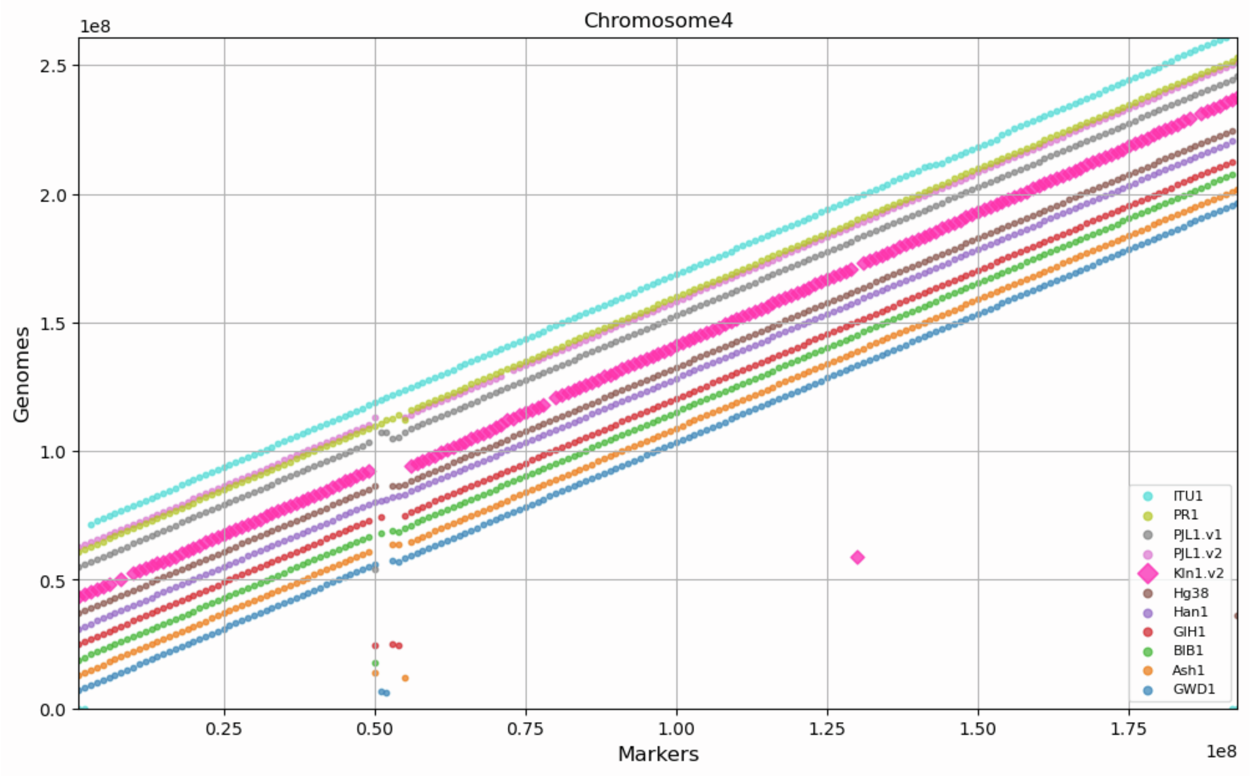
Chromosome4
1e8
2.5
2.0
1.5
1.0
0.5
0.0
Genomes
0.25
0.50
0.75
1.00
1.25
1.50
1.75
Markers
1e8
ITU1
PR1
PJL1.v1
PJL1.v2
Kln1.v2
Hg38
Han1
GIH1
BIB1
Ash1
GWD1

(D)

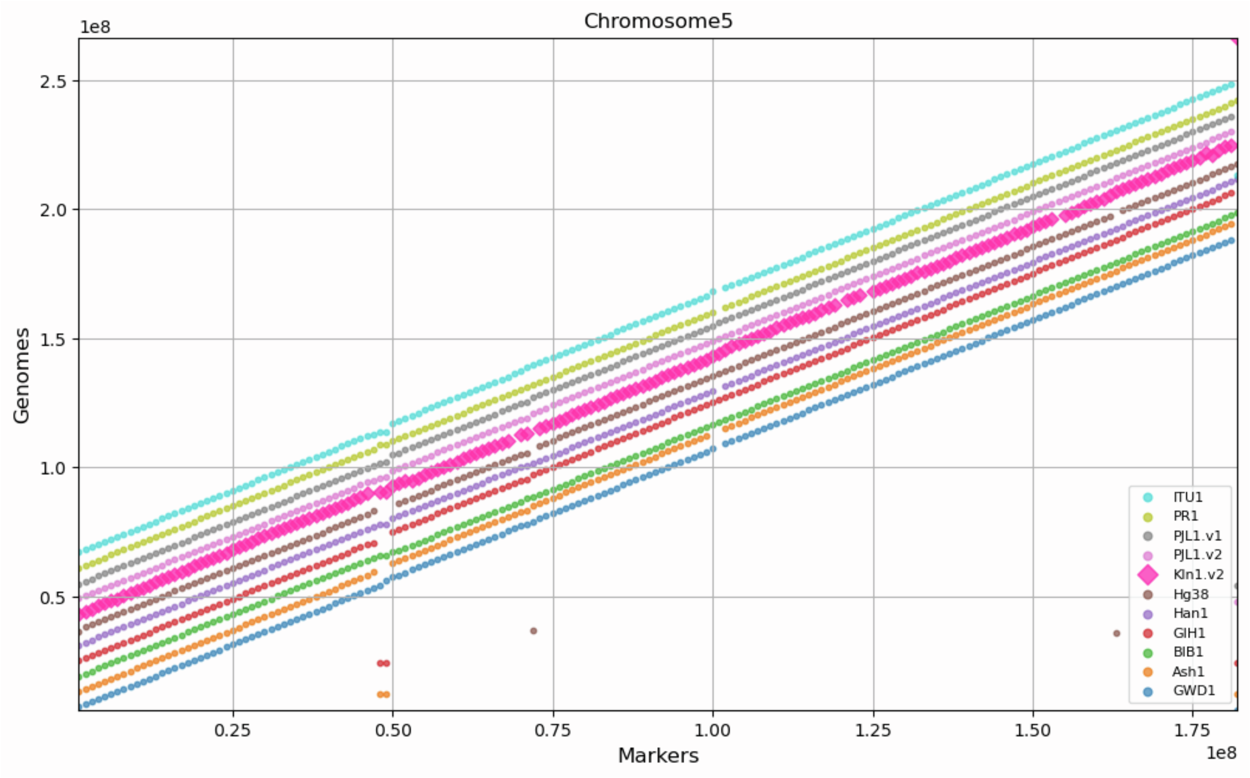
Chromosome5
1e8
2.5
2.0
1.5
1.0
0.5
Genomes
0.25
0.50
0.75
1.00
1.25
1.50
1.75
Markers
1e8
ITU1
PR1
PJL1.v1
PJL1.v2
Kln1.v2
Hg38
Han1
GIH1
BIB1
Ash1
GWD1

(E)

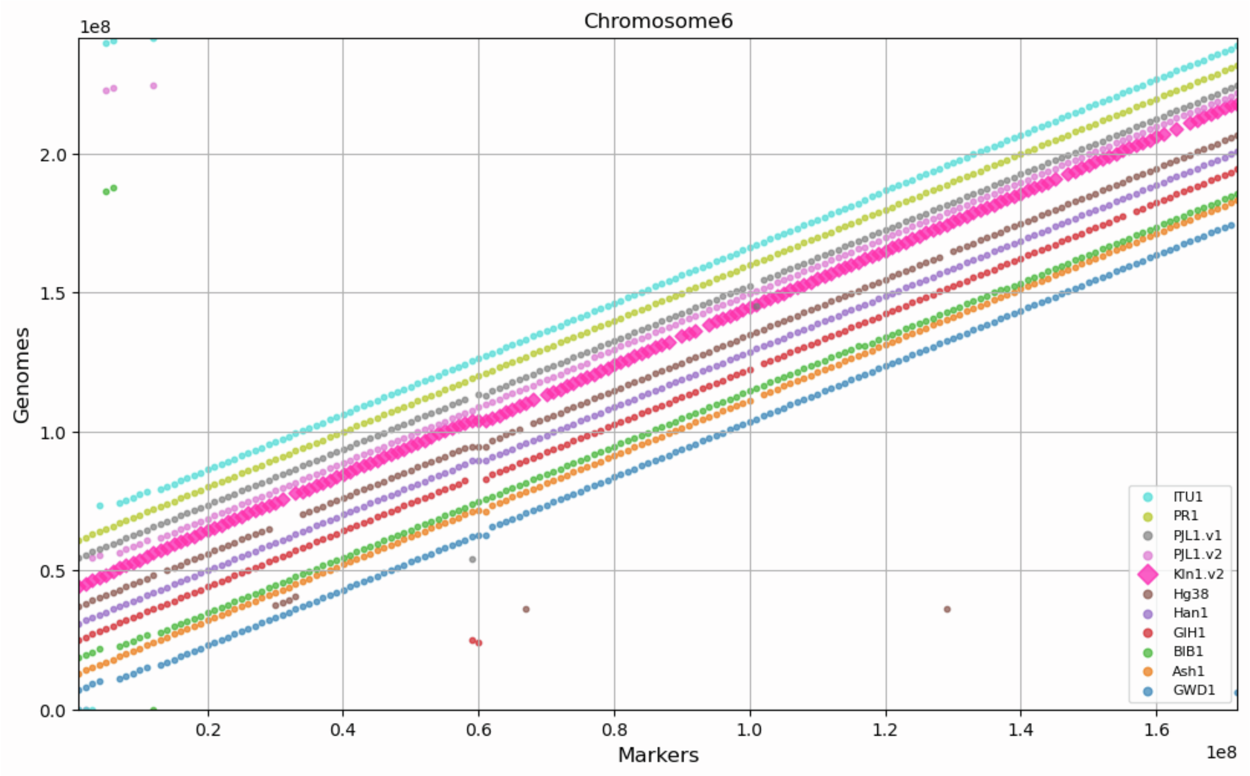
Chromosome6
1e8
2.0
1.5
1.0
0.5
0.0
Genomes
0.2
0.4
0.6
0.8
1.0
1.2
1.4
1.6
Markers
1e8
ITU1
PR1
PJL1.v1
PJL1.v2
Kln1.v2
Hg38
Han1
GIH1
BIB1
Ash1
GWD1

(F)

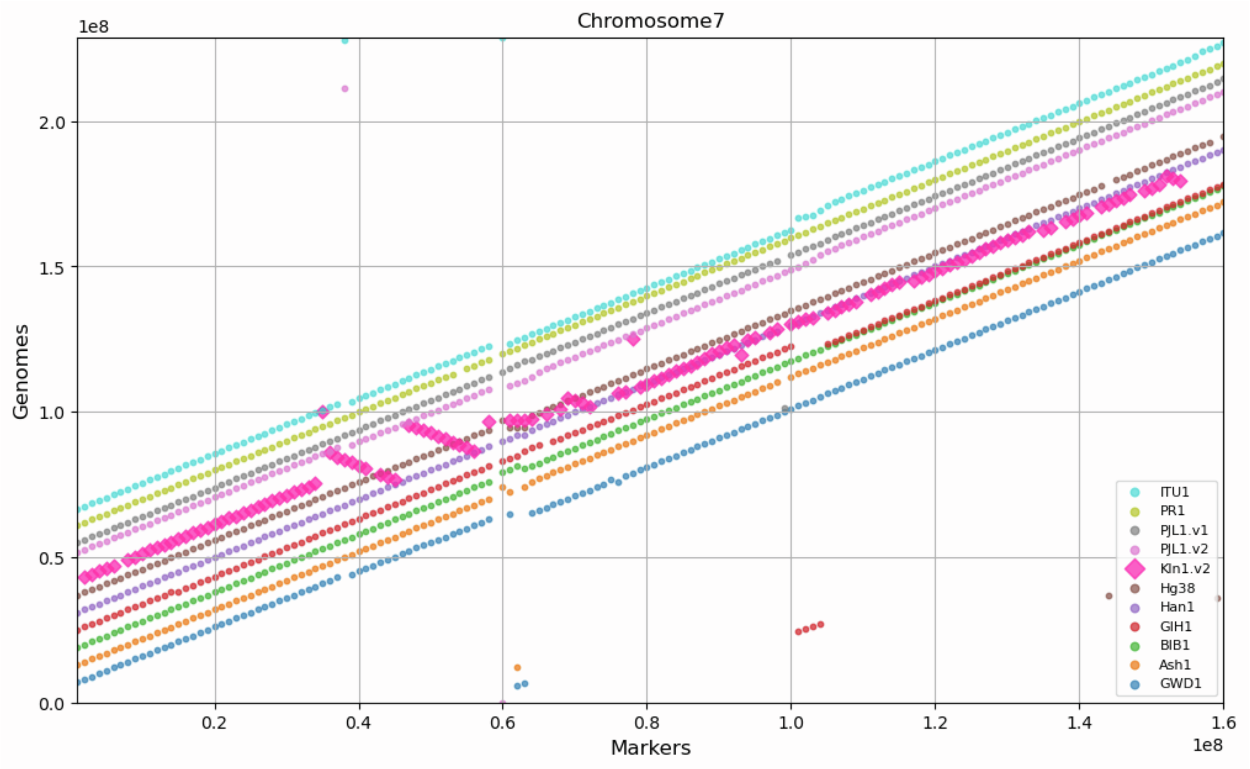
Chromosome7
1e8
2.0
1.5
1.0
0.5
0.0
Genomes
0.2
0.4
0.6
0.8
1.0
1.2
1.4
1.6
Markers
1e8
ITU1
PR1
PJL1.v1
PJL1.v2
Kln1.v2
Hg38
Han1
GIH1
BIB1
Ash1
GWD1

(G)

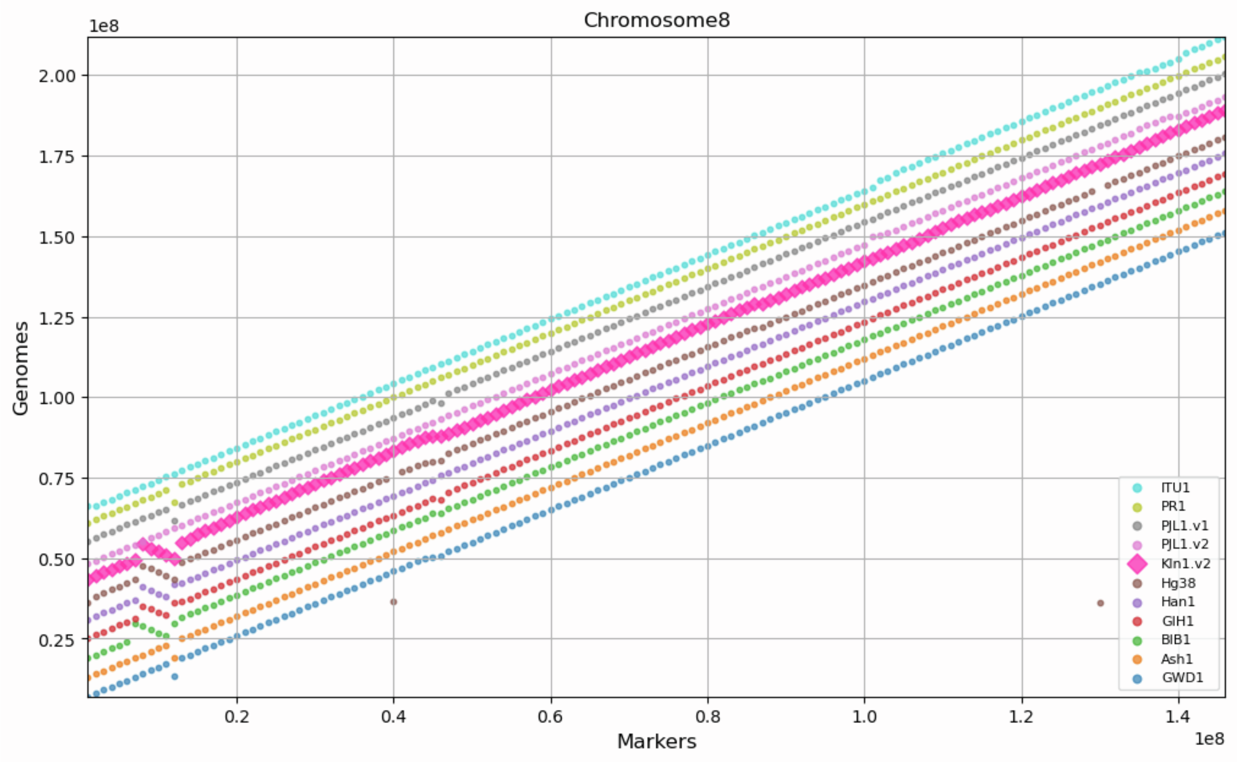
Chromosome8
1e8
2.00
1.75
1.50
1.25
1.00
0.75
0.50
0.25
Genomes
0.2
0.4
0.6
0.8
1.0
1.2
1.4
Markers
1e8
ITU1
PR1
PJL1.v1
PJL1.v2
Kln1.v2
Hg38
Han1
GIH1
BIB1
Ash1
GWD1

(H)

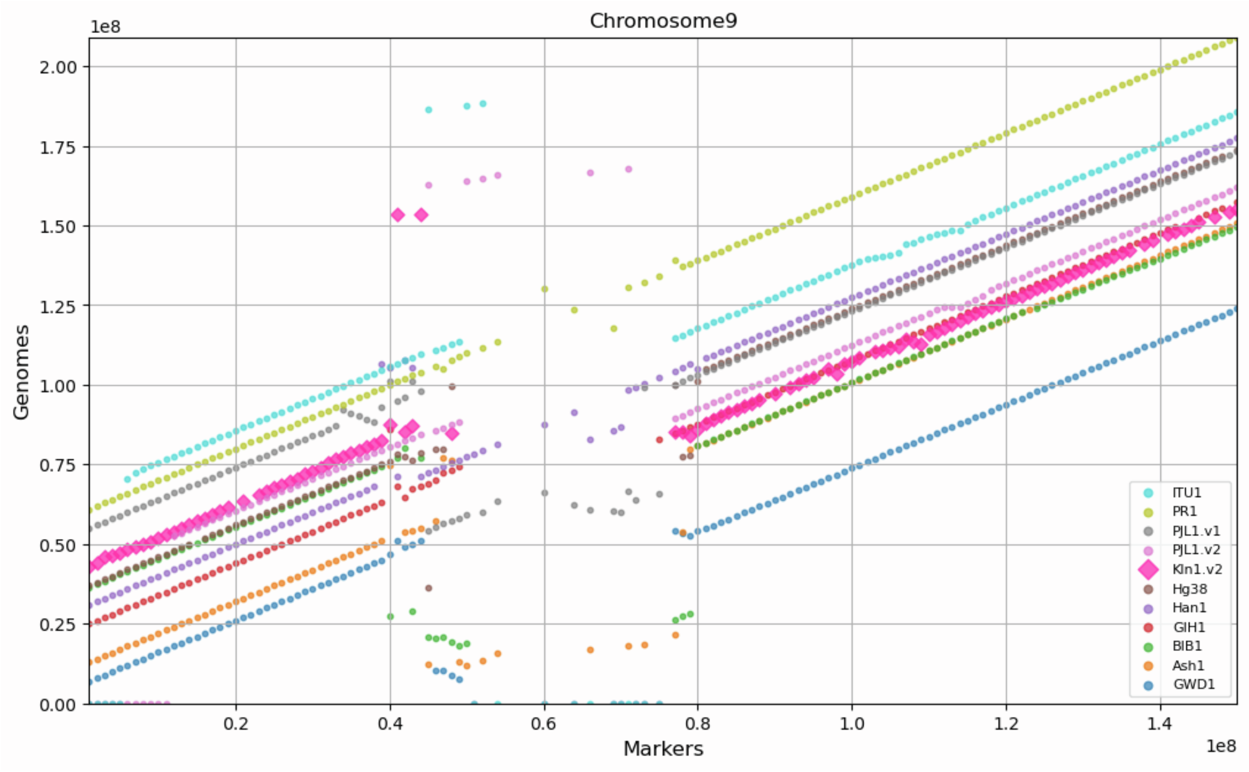
Chromosome9
1e8
2.00
1.75
1.50
1.25
1.00
0.75
0.50
0.25
0.00
Genomes
0.2
0.4
0.6
0.8
1.0
1.2
1.4
Markers
1e8
ITU1
PR1
PJL1.v1
PJL1.v2
Kln1.v2
Hg38
Han1
GIH1
BIB1
Ash1
GWD1

(I)

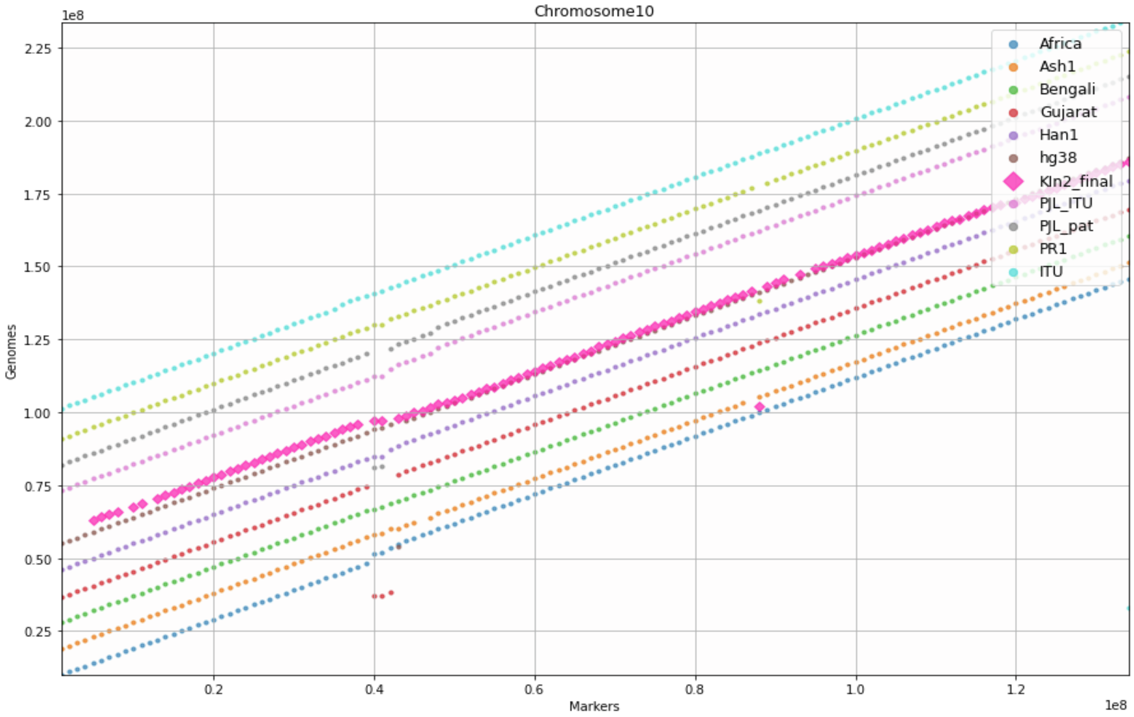
Chromosome10
1e8
2.25
2.00
1.75
1.50
1.25
1.00
0.75
0.50
0.25
Genomes
0.2
0.4
0.6
0.8
1.0
1.2
Markers
1e8
Africa
Ash1
Bengali
Gujarat
Han1
hg38
Kln2_final
PJL_ITU
PJL_pat
PR1
ITU

(J)

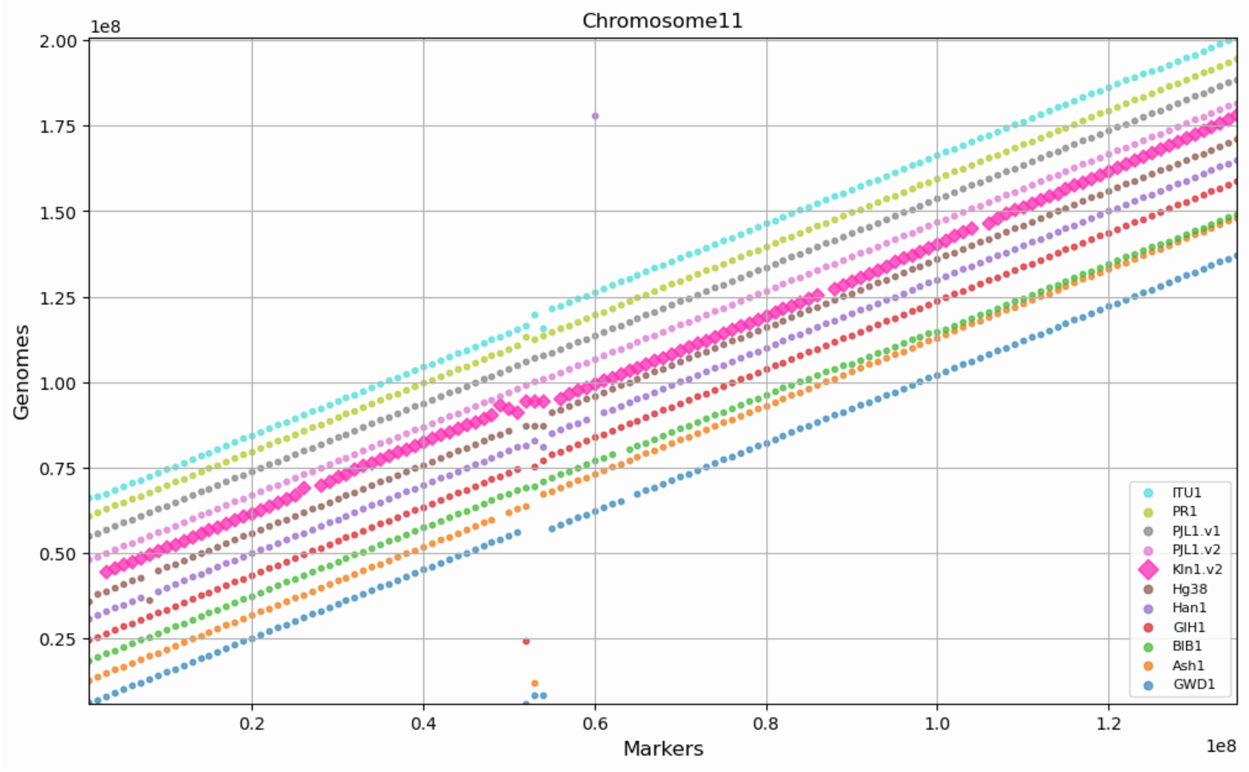
Chromosome11
1e8
2.00
1.75
1.50
1.25
1.00
0.75
0.50
0.25
Genomes
0.2
0.4
0.6
0.8
1.0
1.2
Markers
1e8
ITU1
PR1
PJL1.v1
PJL1.v2
Kln1.v2
Hg38
Han1
GIH1
BIB1
Ash1
GWD1

(K)

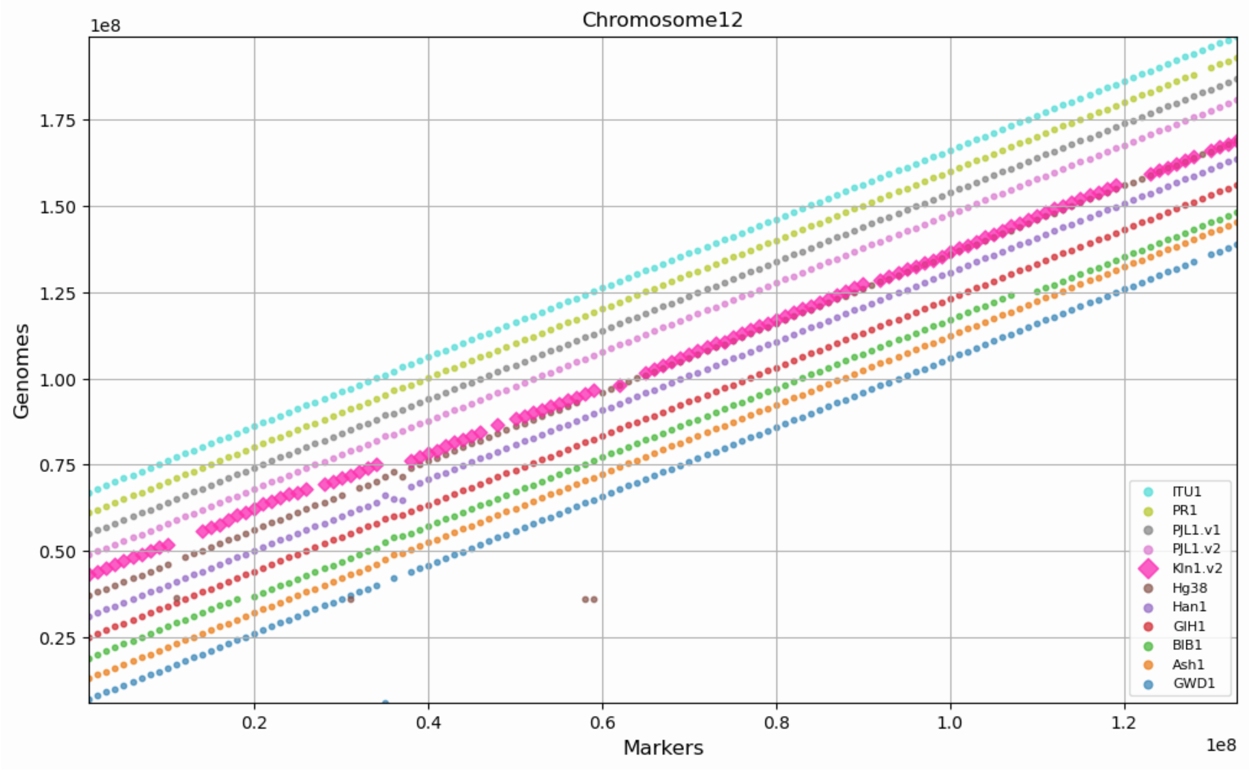
Chromosome12
1e8
1.75
1.50
1.25
1.00
0.75
0.50
0.25
Genomes
0.2
0.4
0.6
0.8
1.0
1.2
Markers
1e8
ITU1
PR1
PJL1.v1
PJL1.v2
Kln1.v2
Hg38
Han1
GIH1
BIB1
Ash1
GWD1

(L)

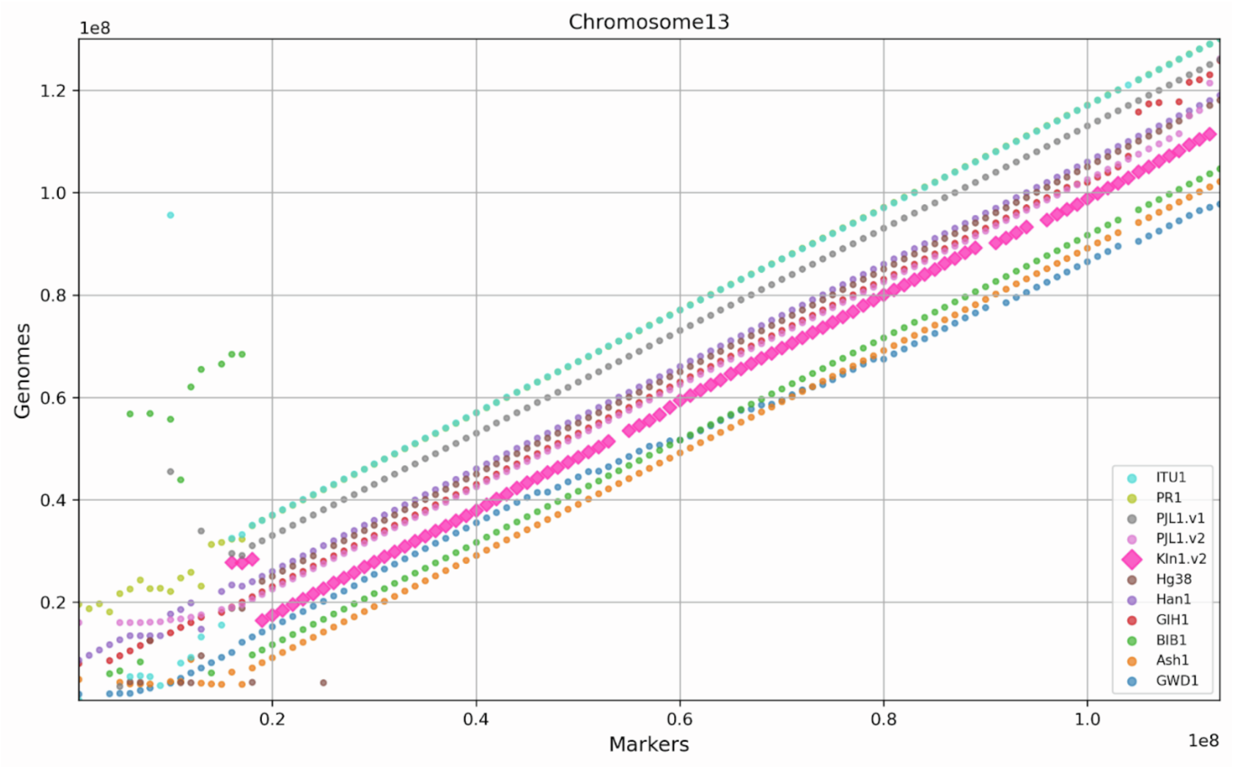
Chromosome13
1e8
1.2
1.0
0.8
0.6
0.4
0.2
Genomes
0.2
0.4
0.6
0.8
1.0
Markers
1e8
ITU1
PR1
PJL1.v1
PJL1.v2
Kln1.v2
Hg38
Han1
GIH1
BIB1
Ash1
GWD1

(M)

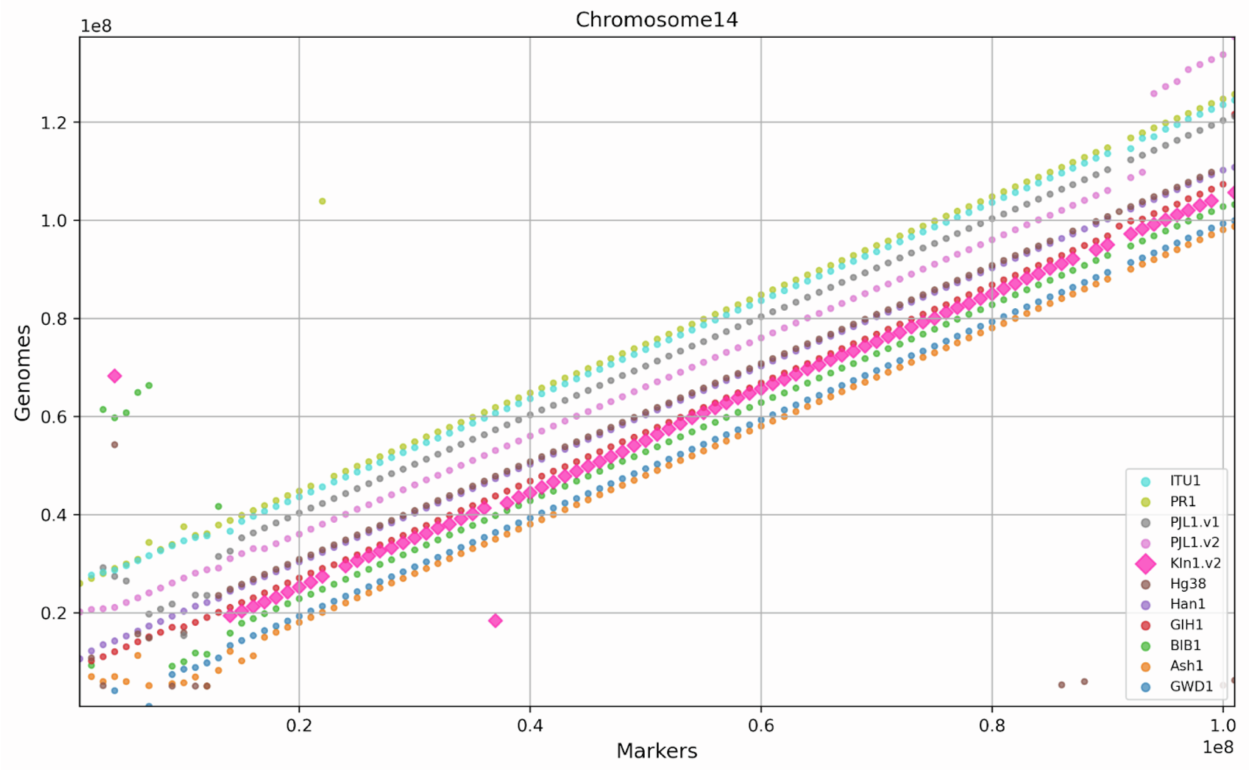
Chromosome14
1e8
1.2
1.0
0.8
0.6
0.4
0.2
Genomes
0.2
0.4
0.6
0.8
1.0
Markers
1e8
ITU1
PR1
PJL1.v1
PJL1.v2
Kln1.v2
Hg38
Han1
GIH1
BIB1
Ash1
GWD1

(N)

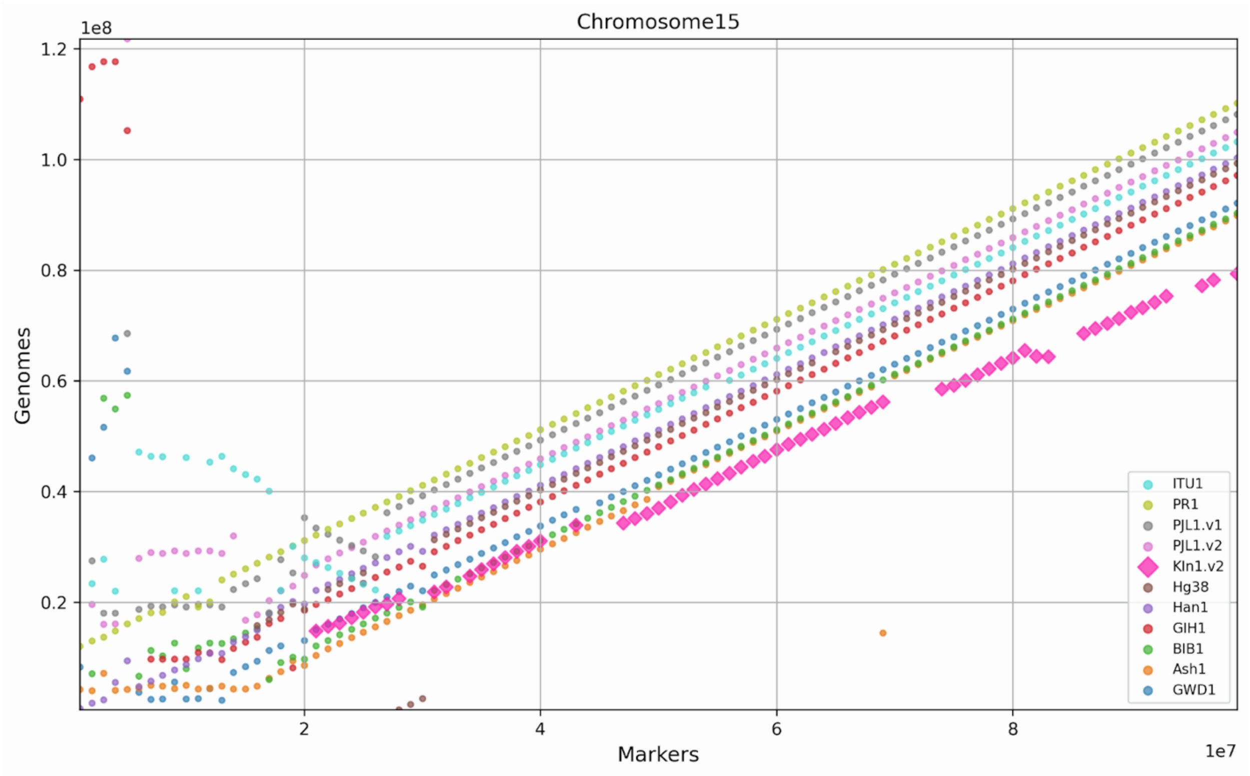
Chromosome15
1e8
1.2
1.0
0.8
0.6
0.4
0.2
Genomes
2
4
6
8
Markers
1e7
ITU1
PR1
PJL1.v1
PJL1.v2
KIn1.v2
Hg38
Han1
GIH1
BIB1
Ash1
GWD1

(O)

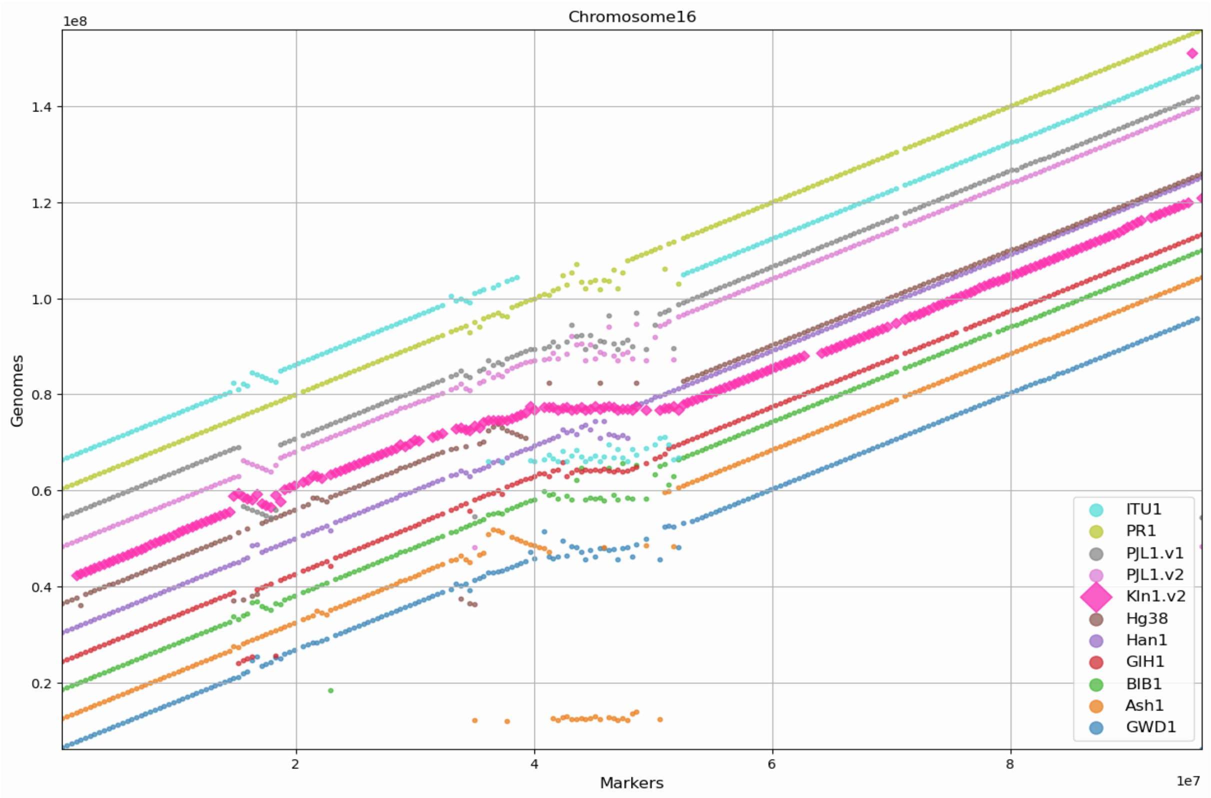
Chromosome16
1e8
1.4
1.2
1.0
0.8
0.6
0.4
0.2
Genomes
2
4
6
8
Markers
1e7
ITU1
PR1
PJL1.v1
PJL1.v2
KIn1.v2
Hg38
Han1
GIH1
BIB1
Ash1
GWD1

(P)

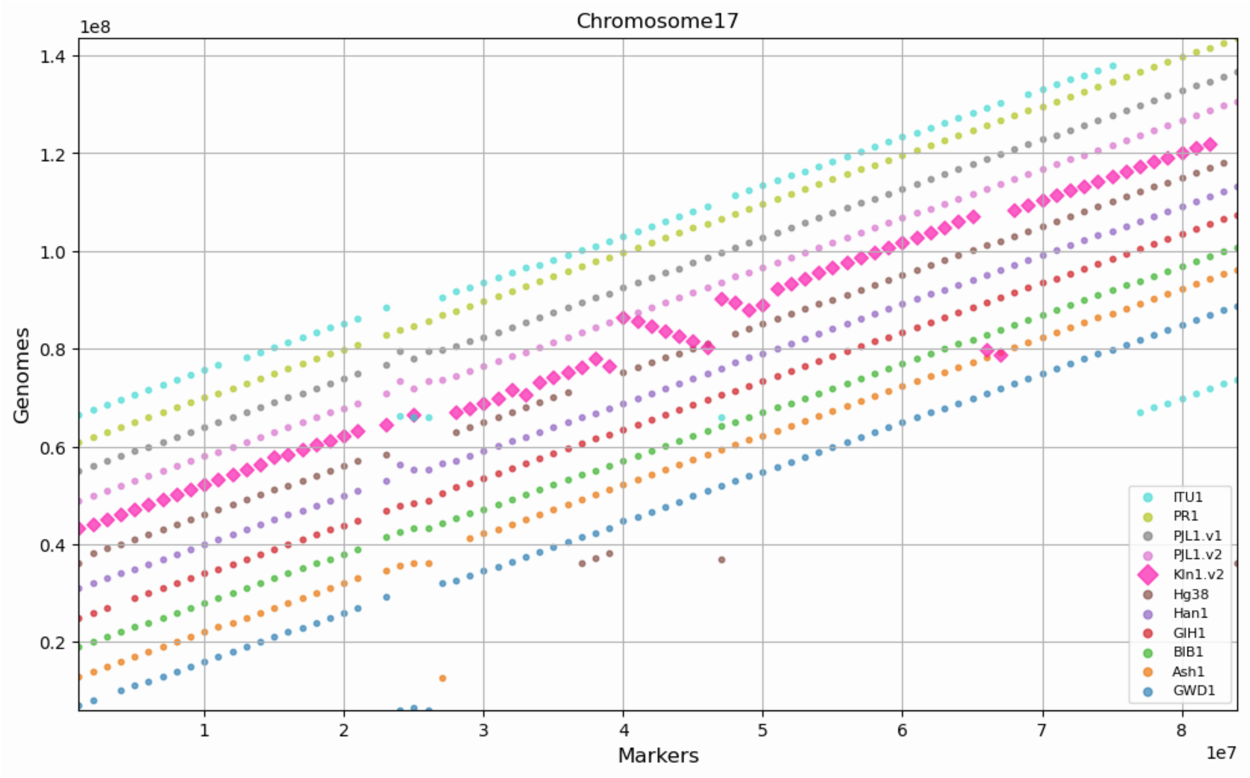
Chromosome17
1e8
1.4
1.2
1.0
0.8
0.6
0.4
0.2
Genomes
1
2
3
4
5
6
7
8
Markers
1e7
ITU1
PR1
PJL1.v1
PJL1.v2
Kln1.v2
Hg38
Han1
GIH1
BIB1
Ash1
GWD1
(Q)

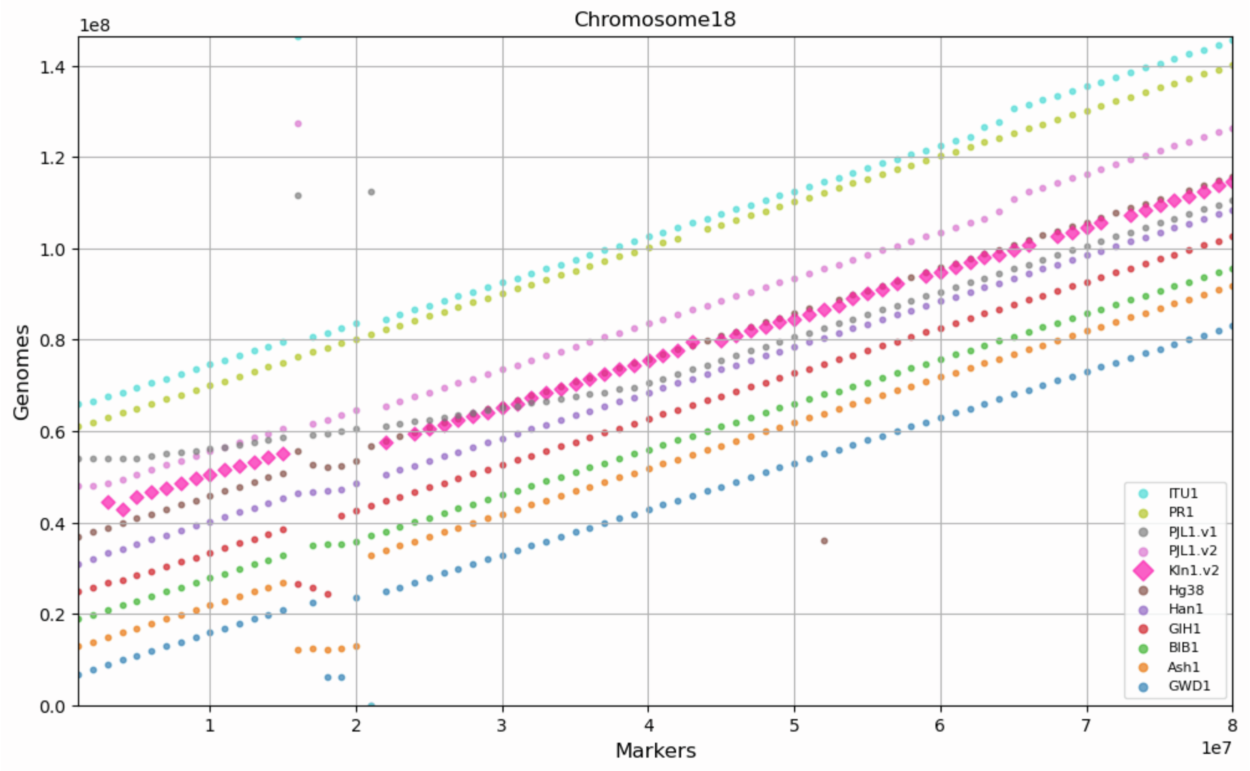
Chromosome18
1e8
1.4
1.2
1.0
0.8
0.6
0.4
0.2
0.0
Genomes
1
2
3
4
5
6
7
8
Markers
1e7
ITU1
PR1
PJL1.v1
PJL1.v2
Kln1.v2
Hg38
Han1
GIH1
BIB1
Ash1
GWD1
(R)

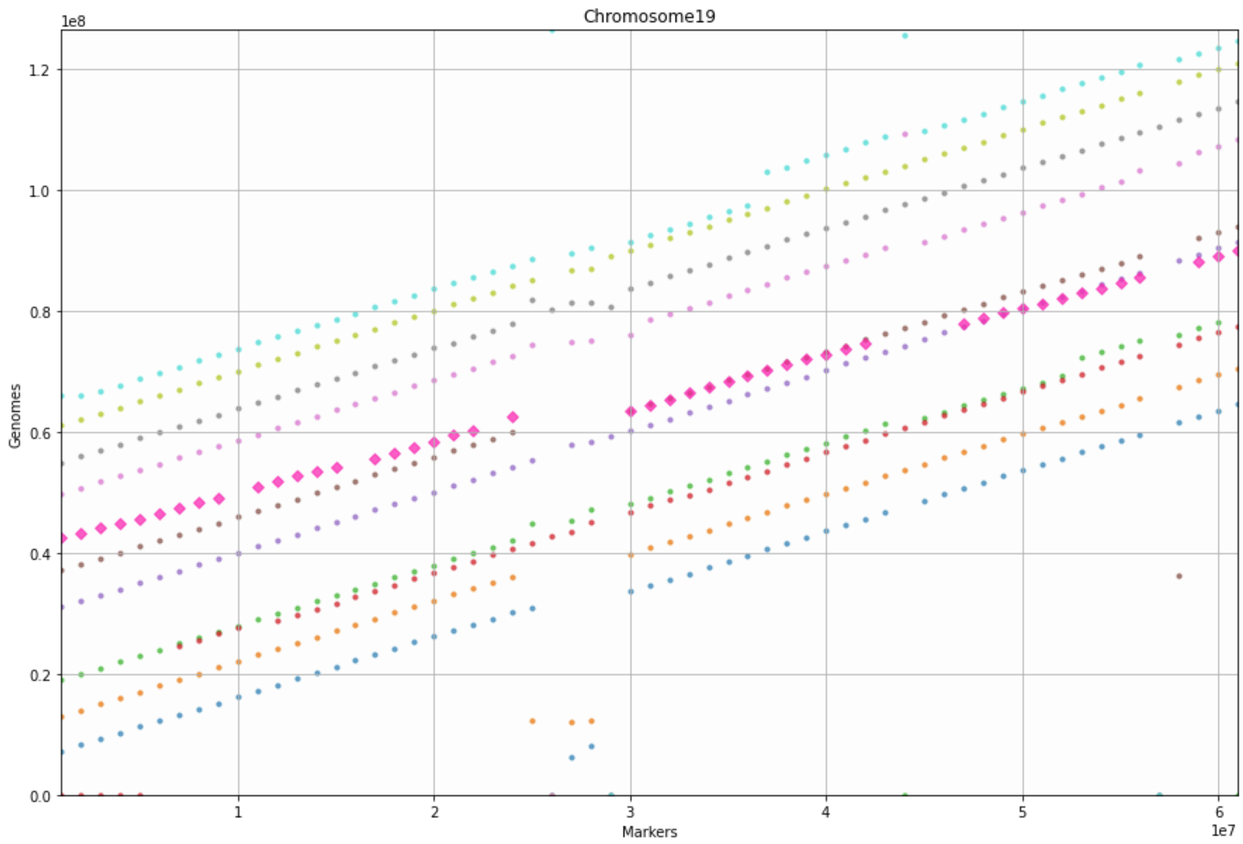
Chromosome19
1e8
1.2
1.0
0.8
0.6
0.4
0.2
0.0
Genomes
1
2
3
4
5
6
1e7
Markers

(S)

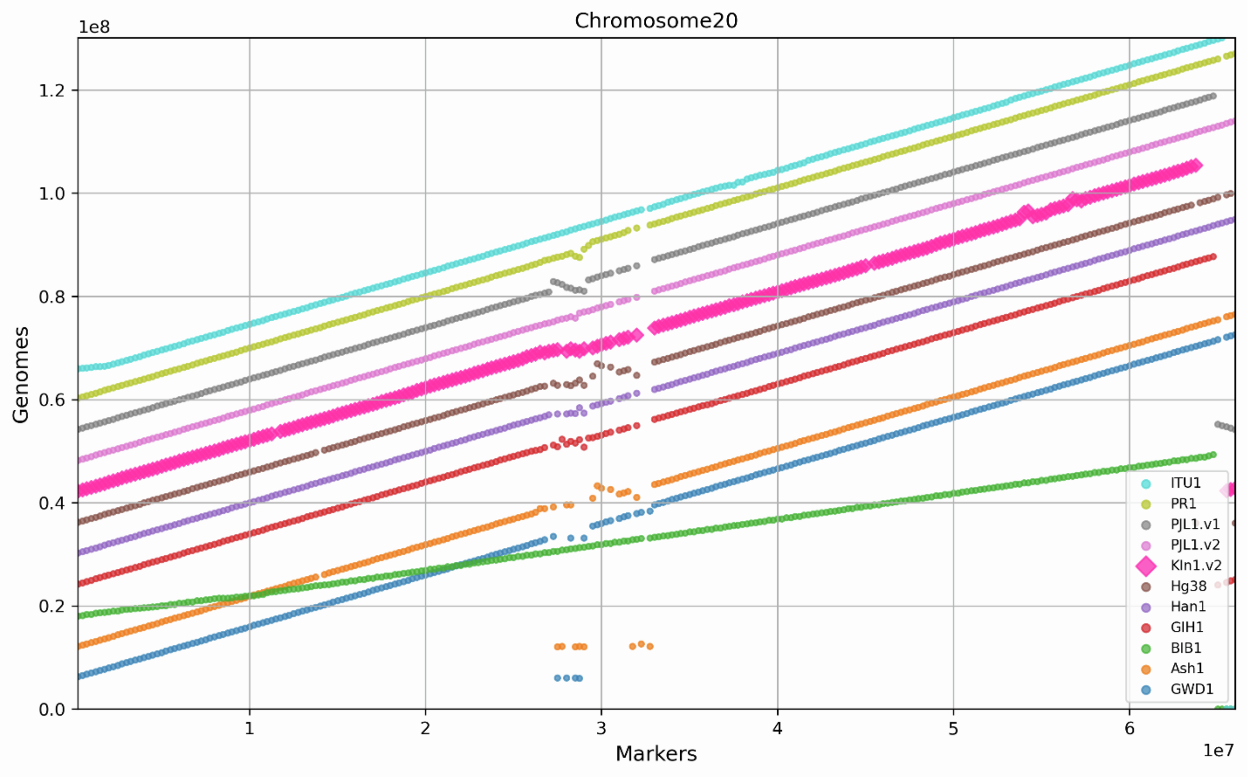
Chromosome20
1e8
1.2
1.0
0.8
0.6
0.4
0.2
0.0
Genomes
1
2
3
4
5
6
1e7
Markers
ITU1
PR1
PJL1.v1
PJL1.v2
Kln1.v2
Hg38
Han1
GIH1
BIB1
Ash1
GWD1

(T)

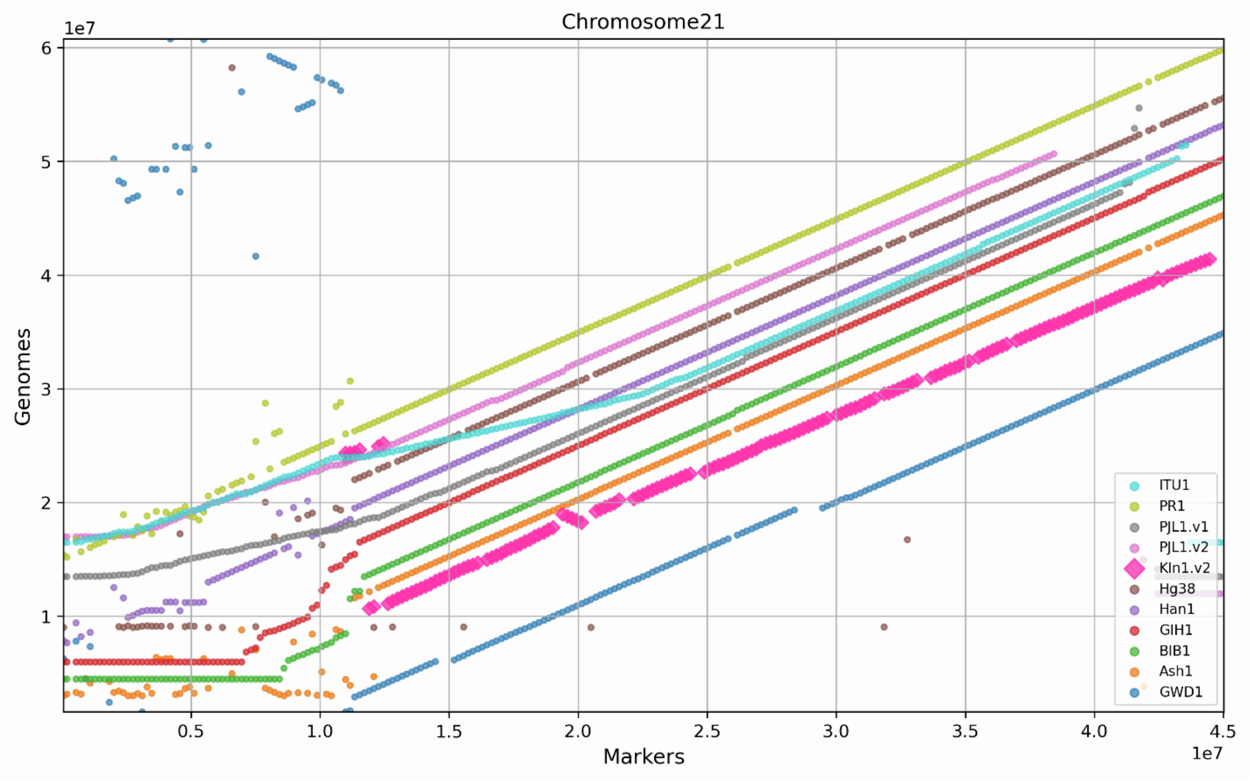
Chromosome21
1e7
Genomes
Markers
1e7
ITU1
PR1
PJL1.v1
PJL1.v2
Kln1.v2
Hg38
Han1
GIH1
BIB1
Ash1
GWD1

(U)

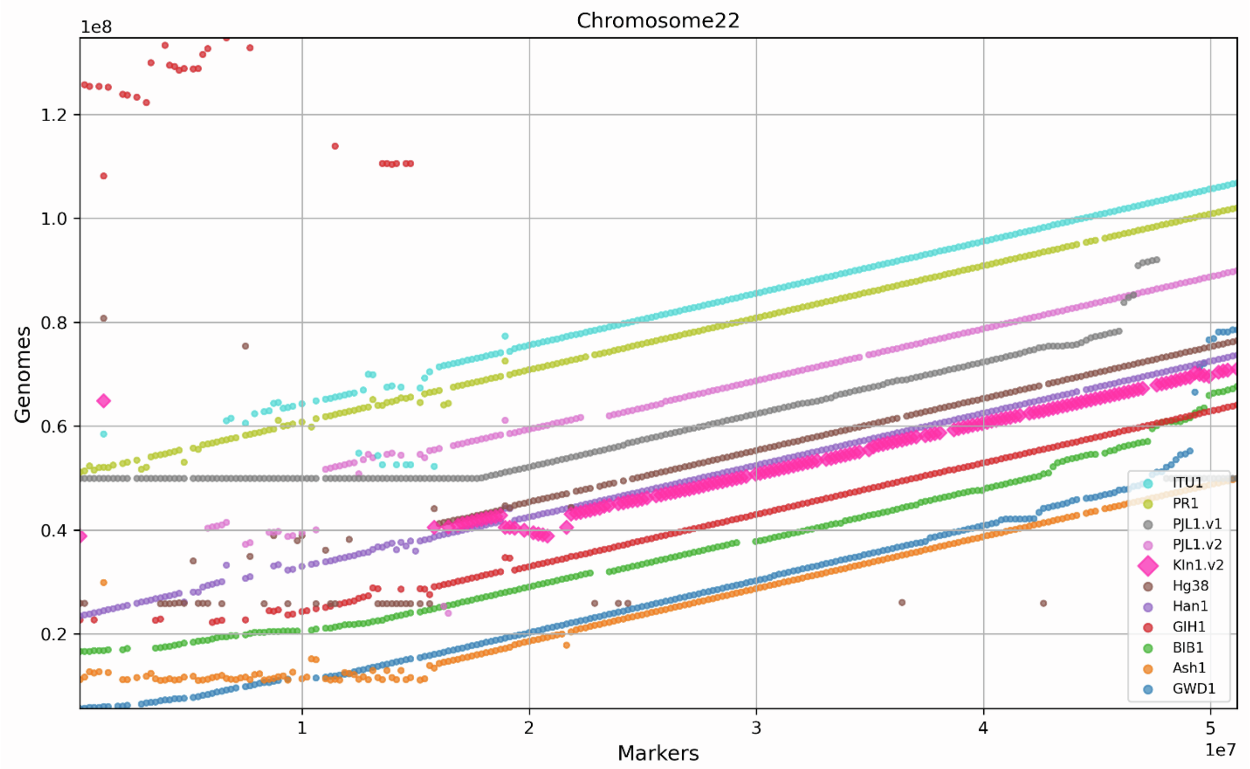
Chromosome22
1e8
Genomes
Markers
1e7
ITU1
PR1
PJL1.v1
PJL1.v2
Kln1.v2
Hg38
Han1
GIH1
BIB1
Ash1
GWD1

(V)

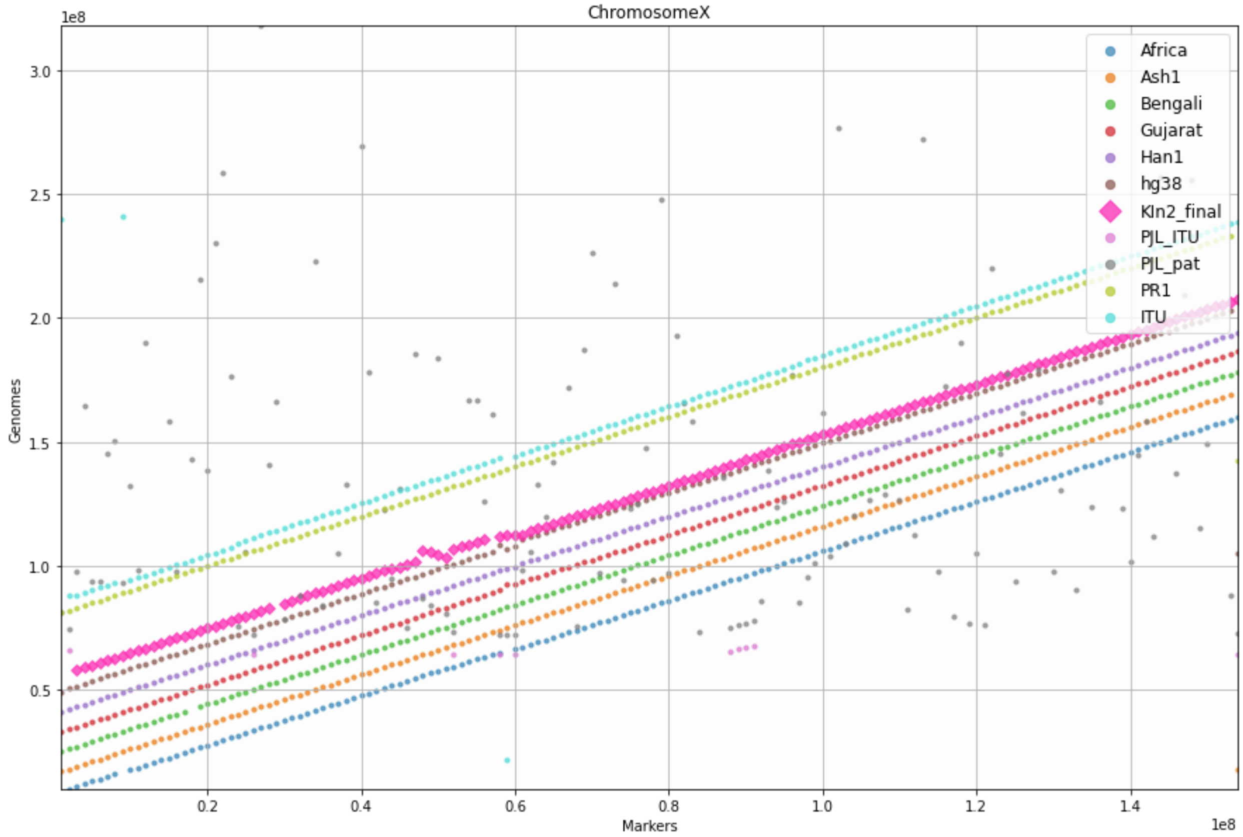

(W)

Supplementary Figure S1 (A-W): shows comparative dot plot of individuals' chromosomes from several genomes against virtual markers every 1Mb from T2T assembly.

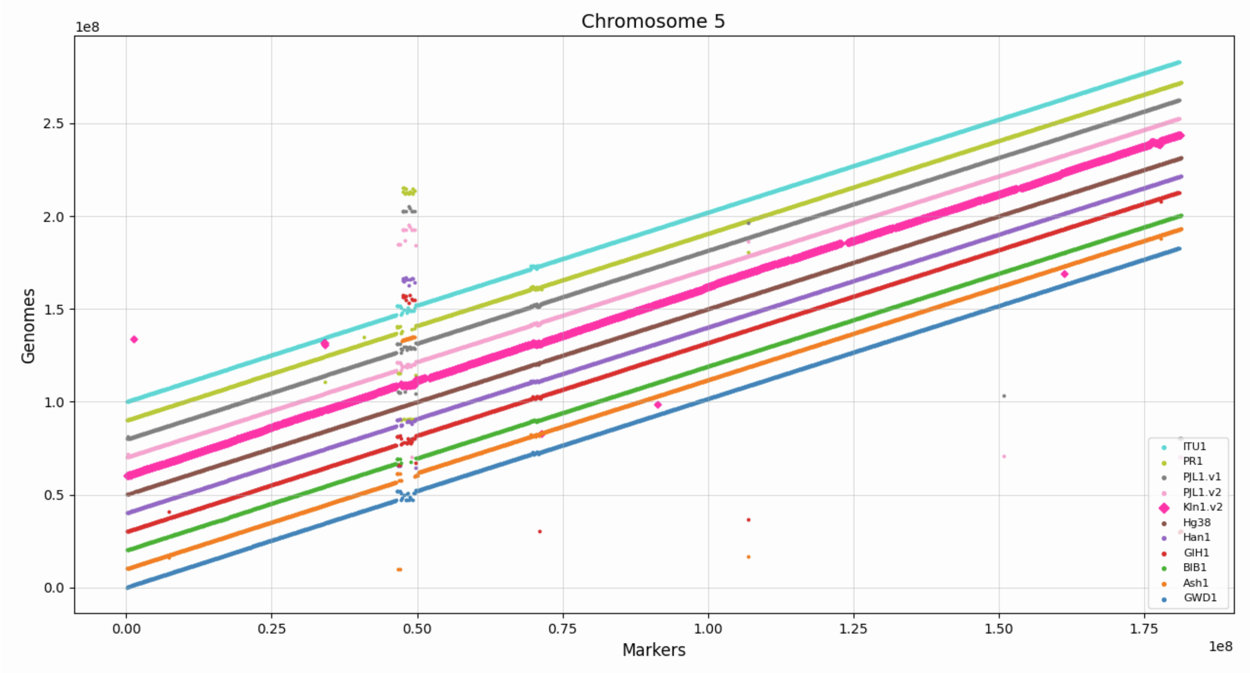
Chromosome 5
1e8
Genomes
Markers
1e8
0.0
0.5
1.0
1.5
2.0
2.5
0.00
0.25
0.50
0.75
1.00
1.25
1.50
1.75
ITU1
PR1
PJL1.v1
PJL1.v2
Kln1.v2
Hg38
Han1
GIH1
BIB1
Ash1
GWD1

(A)

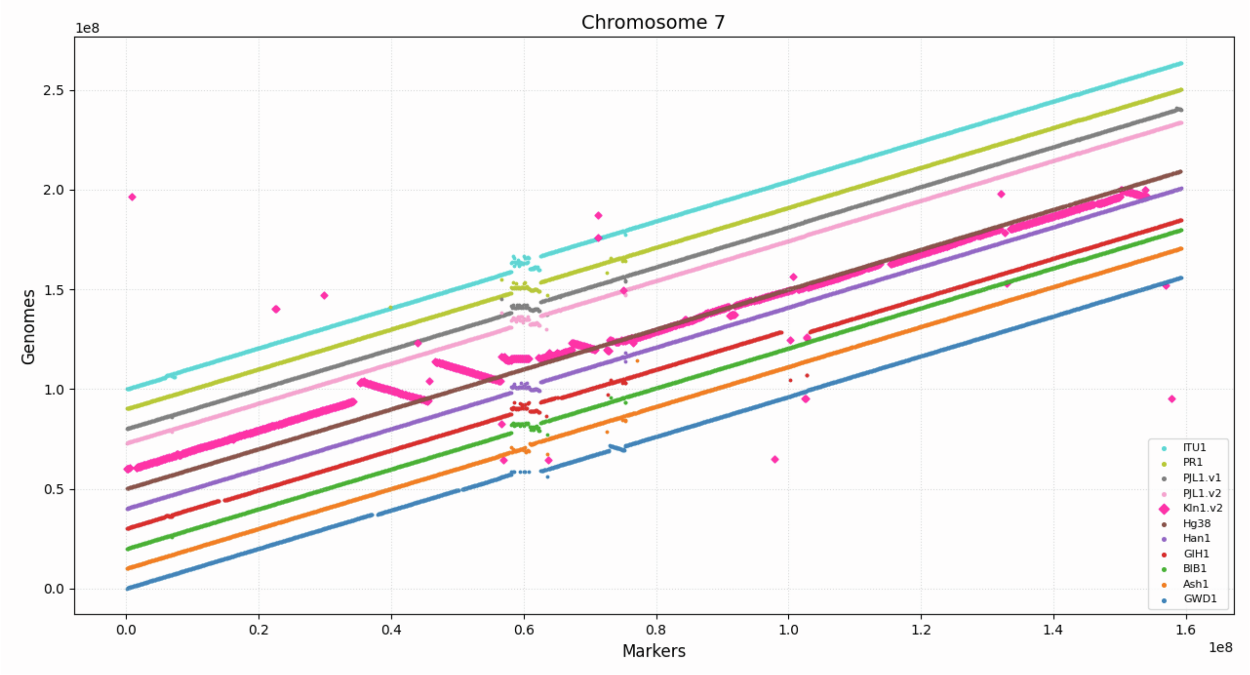
Chromosome 7
1e8
Genomes
Markers
1e8
0.0
0.5
1.0
1.5
2.0
2.5
0.0
0.2
0.4
0.6
0.8
1.0
1.2
1.4
1.6
ITU1
PR1
PJL1.v1
PJL1.v2
Kln1.v2
Hg38
Han1
GIH1
BIB1
Ash1
GWD1

(B)

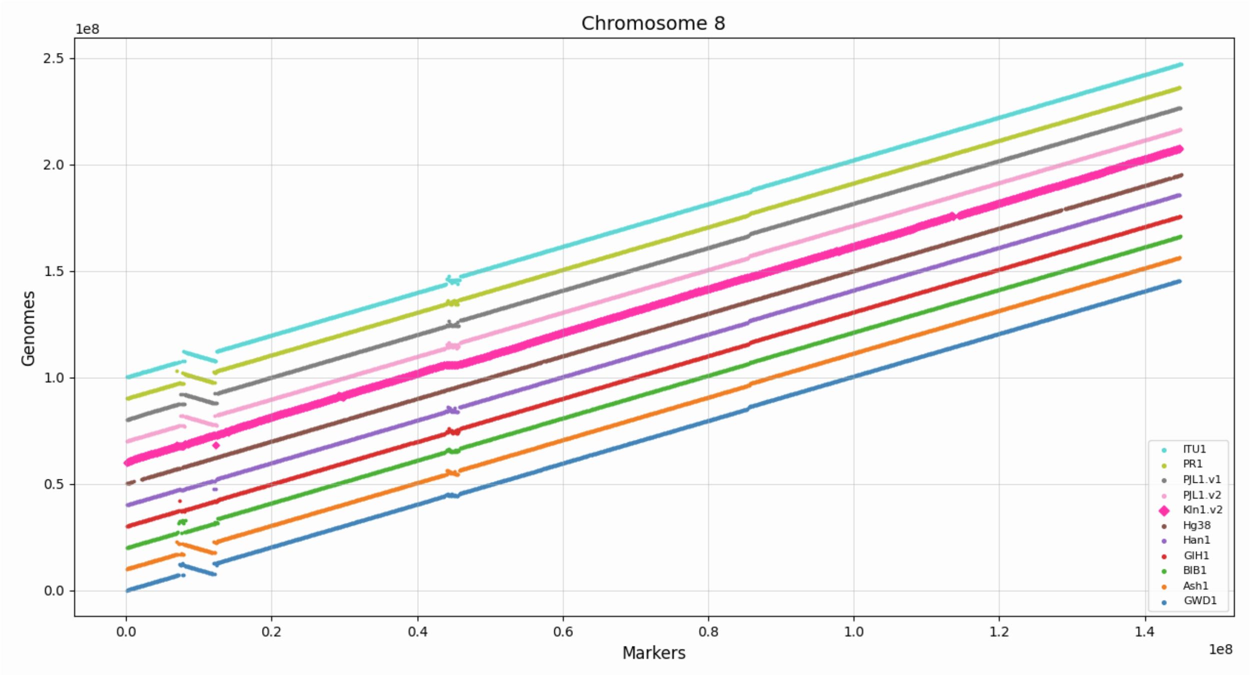
Chromosome 8
1e8
2.5
2.0
1.5
1.0
0.5
0.0
Genomes
0.0
0.2
0.4
0.6
0.8
1.0
1.2
1.4
Markers
1e8
ITU1
PR1
PJL1.v1
PJL1.v2
Kln1.v2
Hg38
Han1
GIH1
BIB1
Ash1
GWD1

(C)

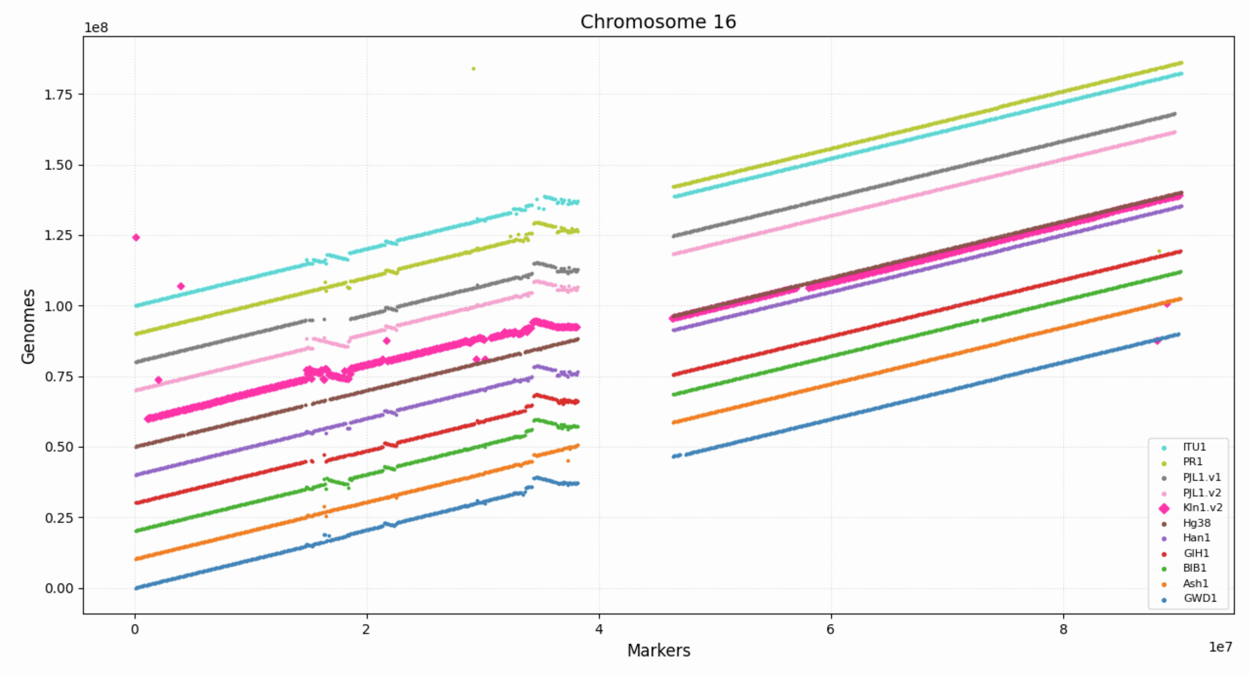
Chromosome 16
1e8
1.75
1.50
1.25
1.00
0.75
0.50
0.25
0.00
Genomes
0
2
4
6
8
Markers
1e7
ITU1
PR1
PJL1.v1
PJL1.v2
Kln1.v2
Hg38
Han1
GIH1
BIB1
Ash1
GWD1

(D)

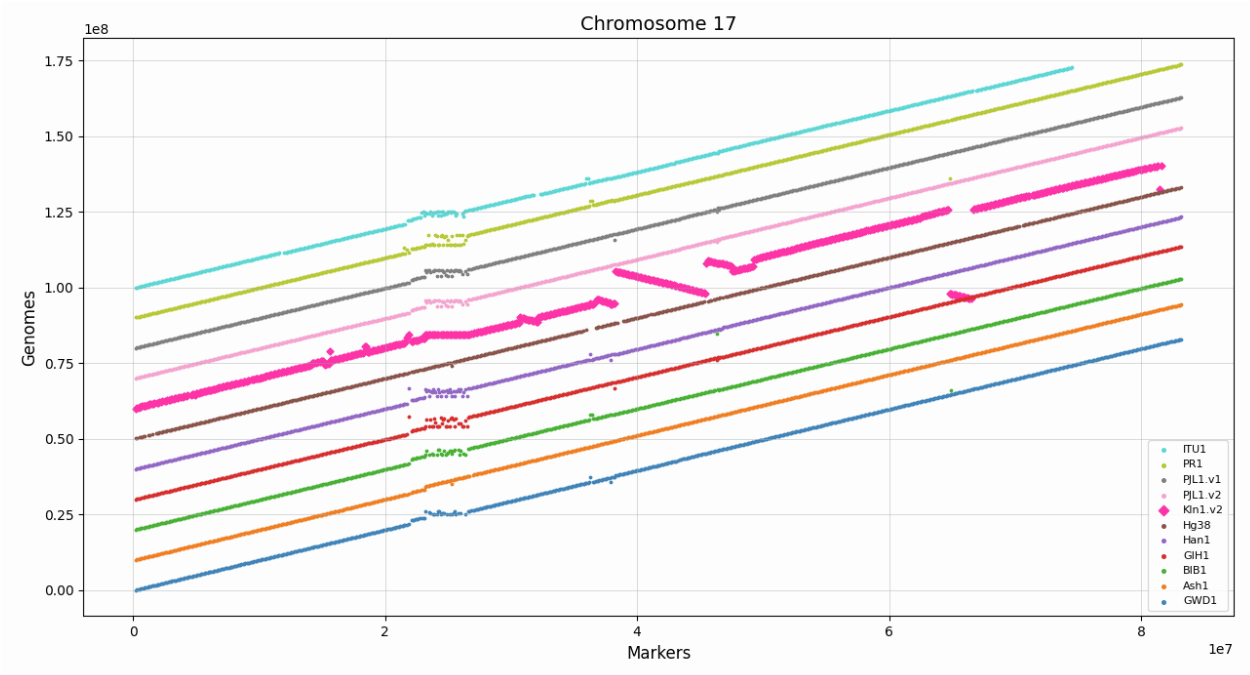

(E)

Supplementary Figure S2 (A-E): shows comparative dot plot of individuals' chromosomes from several genomes against virtual markers every 100kb from hg38 assembly.

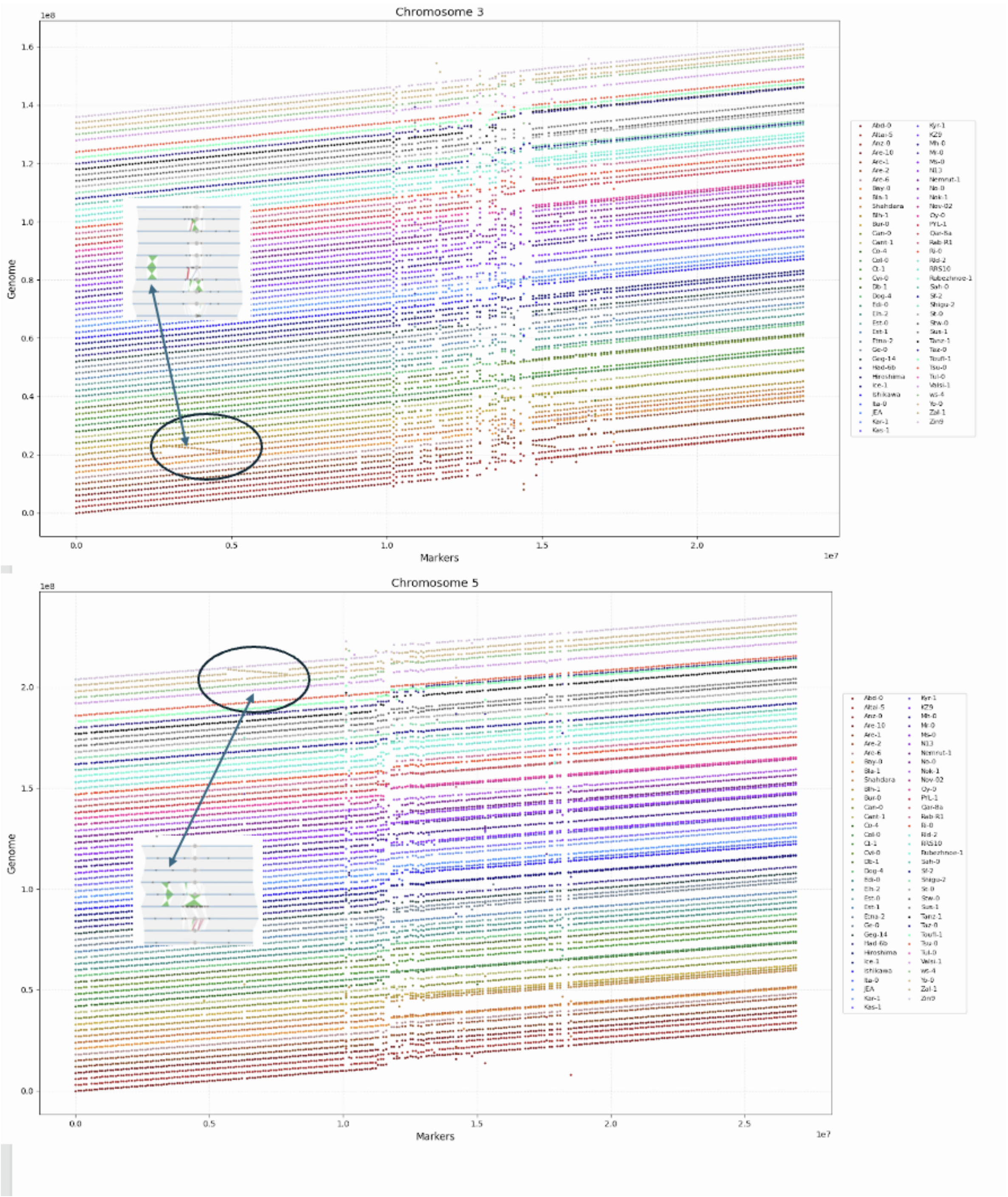

Supplementary Figure S3: Comparison of chromosome 3 and 5 of 69 accession of Arabidopsis (PMID: 38605175) compared to the virtual markers from the reference Arabidopsis genome.

(https://ftp.ncbi.nlm.nih.gov/genomes/all/GCF/000/001/735/GCF_000001735.4_TAIR10.1/GCF_000001735.4_TAIR10.1_genomic.fna.gz). Inset shows the part of Figure 3 from PMID: 38605175

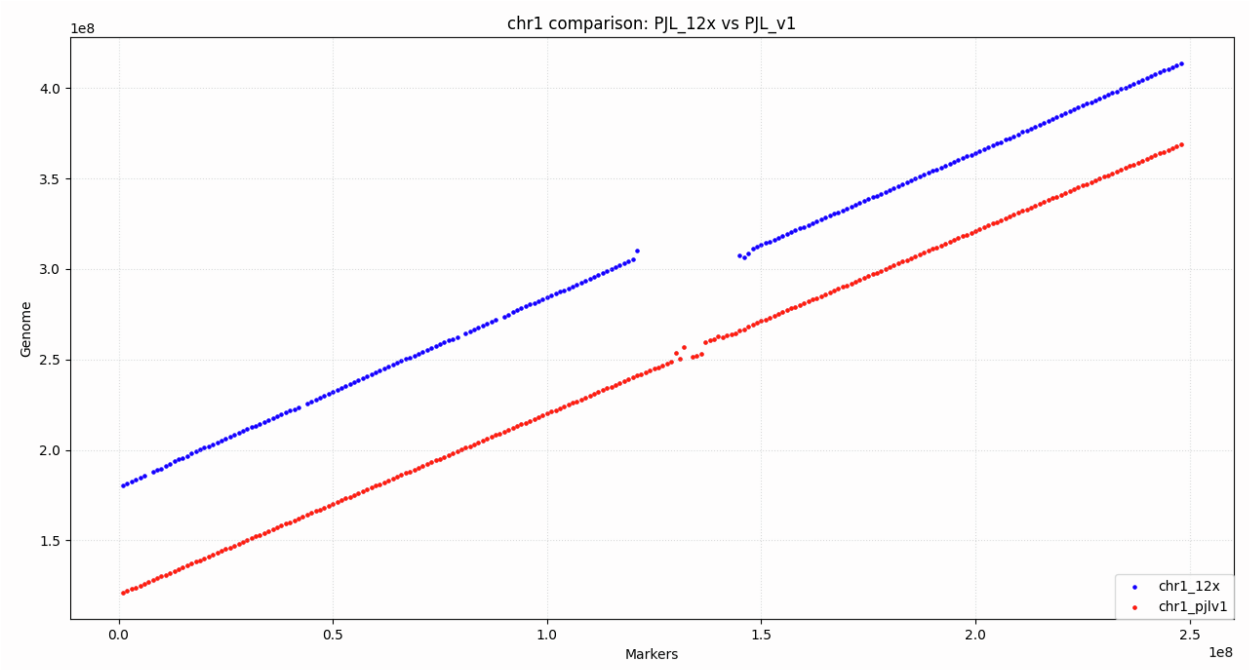
chr1 comparison: PJL_12x vs PJL_v1
1e8
4.0
3.5
3.0
2.5
2.0
1.5
Genome
0.0
0.5
1.0
1.5
2.0
2.5
1e8
Markers
chr1_12x
chr1_pjlv1

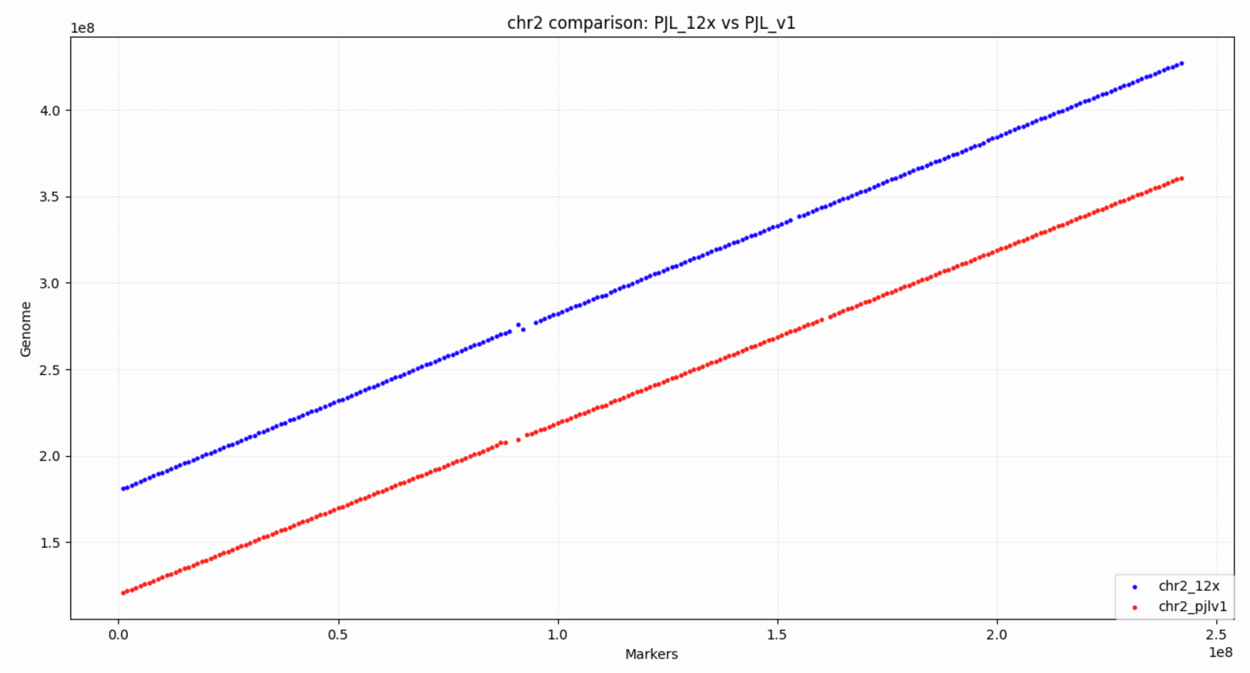
chr2 comparison: PJL_12x vs PJL_v1
1e8
4.0
3.5
3.0
2.5
2.0
1.5
Genome
0.0
0.5
1.0
1.5
2.0
2.5
1e8
Markers
chr2_12x
chr2_pjlv1

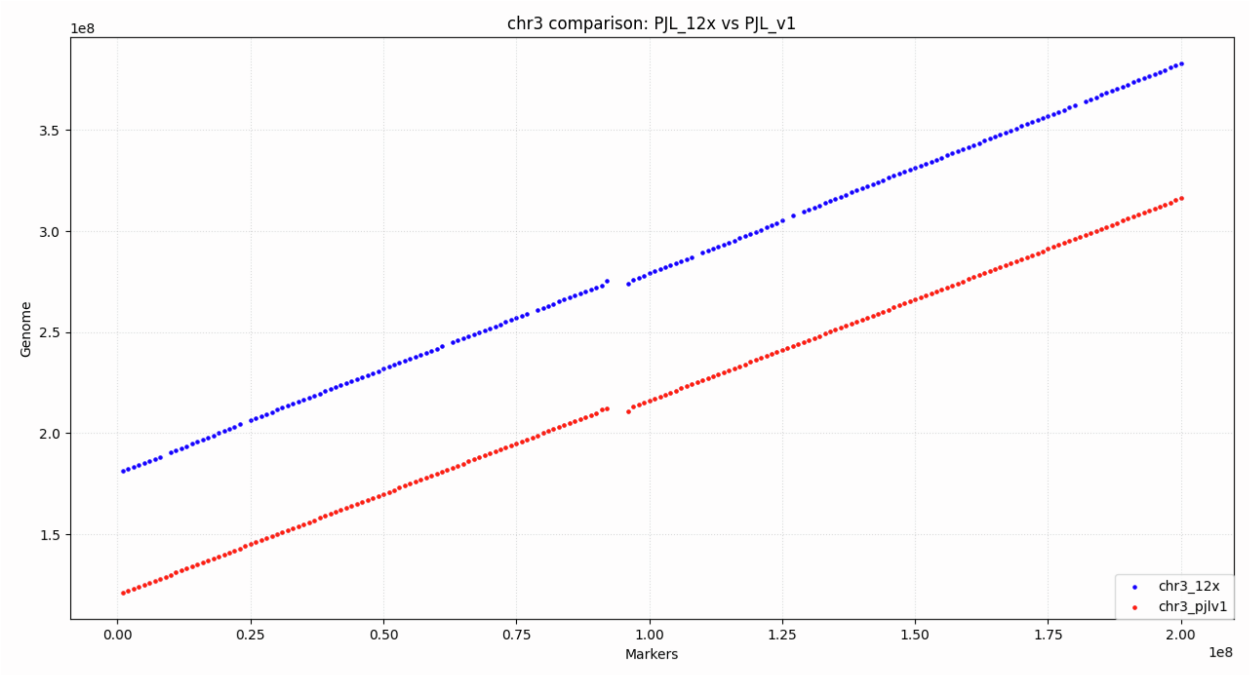
chr3 comparison: PJL_12x vs PJL_v1
1e8
3.5
3.0
2.5
2.0
1.5
Genome
0.00
0.25
0.50
0.75
1.00
1.25
1.50
1.75
2.00
1e8
Markers
chr3_12x
chr3_pjlv1

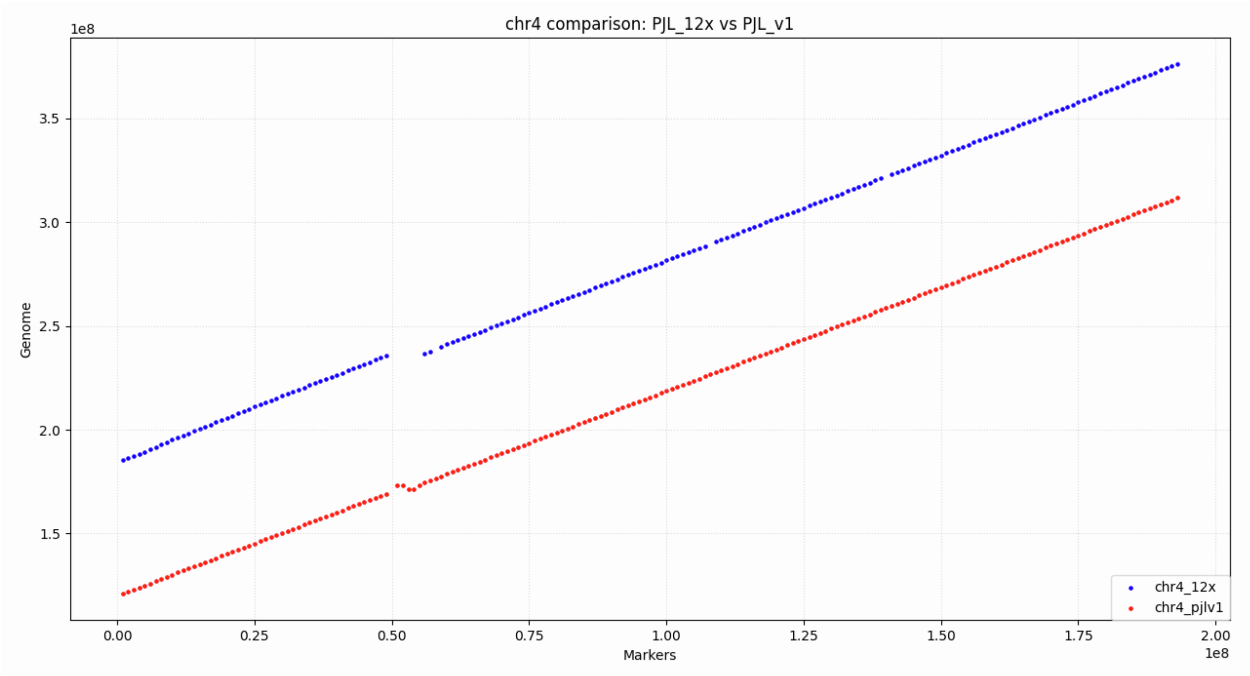
chr4 comparison: PJL_12x vs PJL_v1
1e8
3.5
3.0
2.5
2.0
1.5
Genome
0.00
0.25
0.50
0.75
1.00
1.25
1.50
1.75
2.00
1e8
Markers
chr4_12x
chr4_pjlv1

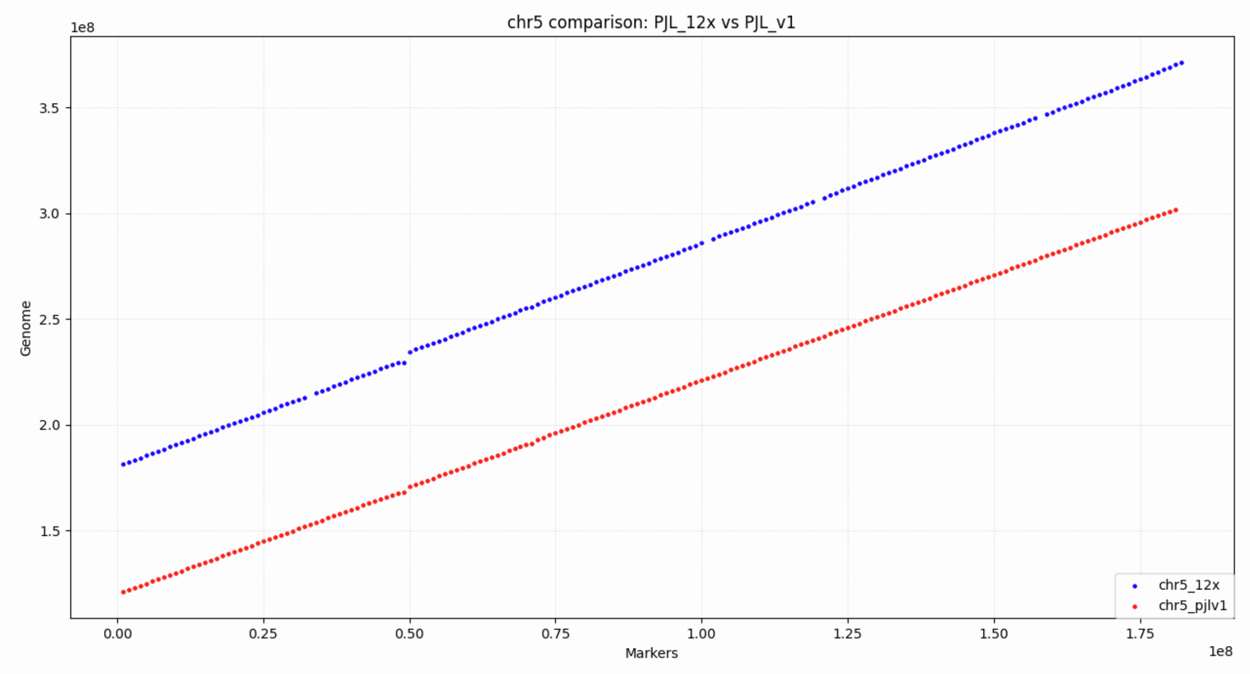
chr5 comparison: PJL_12x vs PJL_v1
1e8
3.5
3.0
2.5
2.0
1.5
Genome
0.00
0.25
0.50
0.75
1.00
1.25
1.50
1.75
1e8
Markers
chr5_12x
chr5_pjlv1

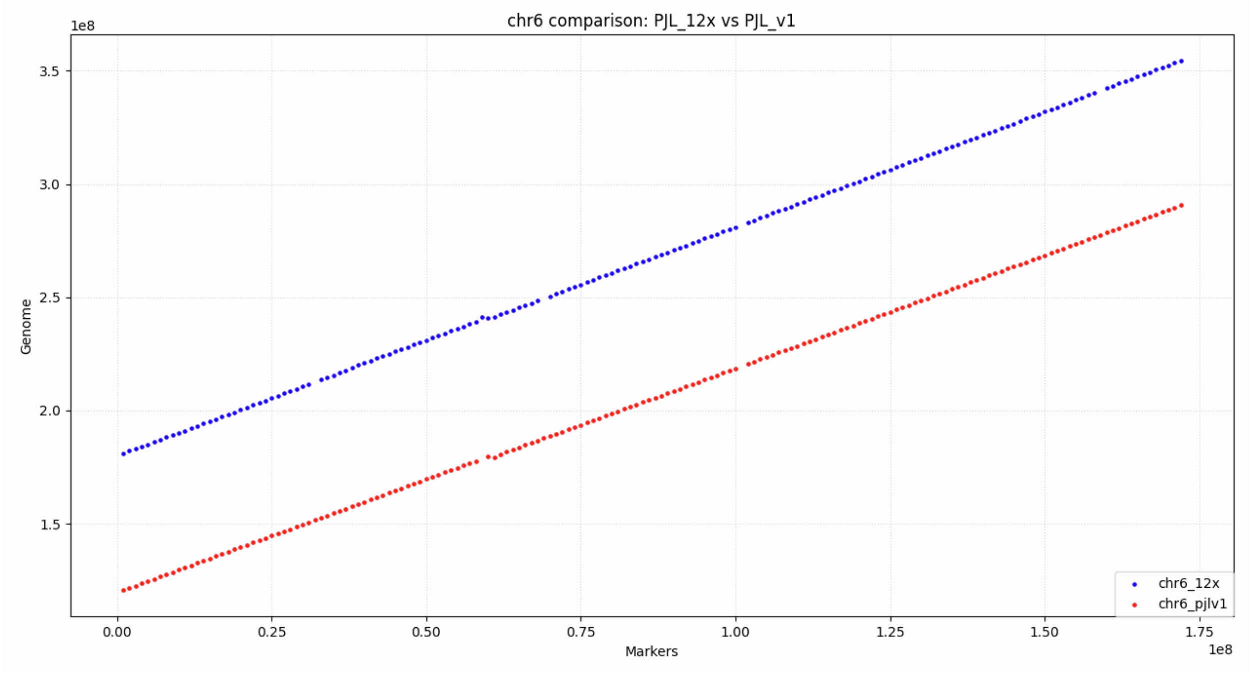
chr6 comparison: PJL_12x vs PJL_v1
1e8
3.5
3.0
2.5
2.0
1.5
Genome
0.00
0.25
0.50
0.75
1.00
1.25
1.50
1.75
1e8
Markers
chr6_12x
chr6_pjlv1

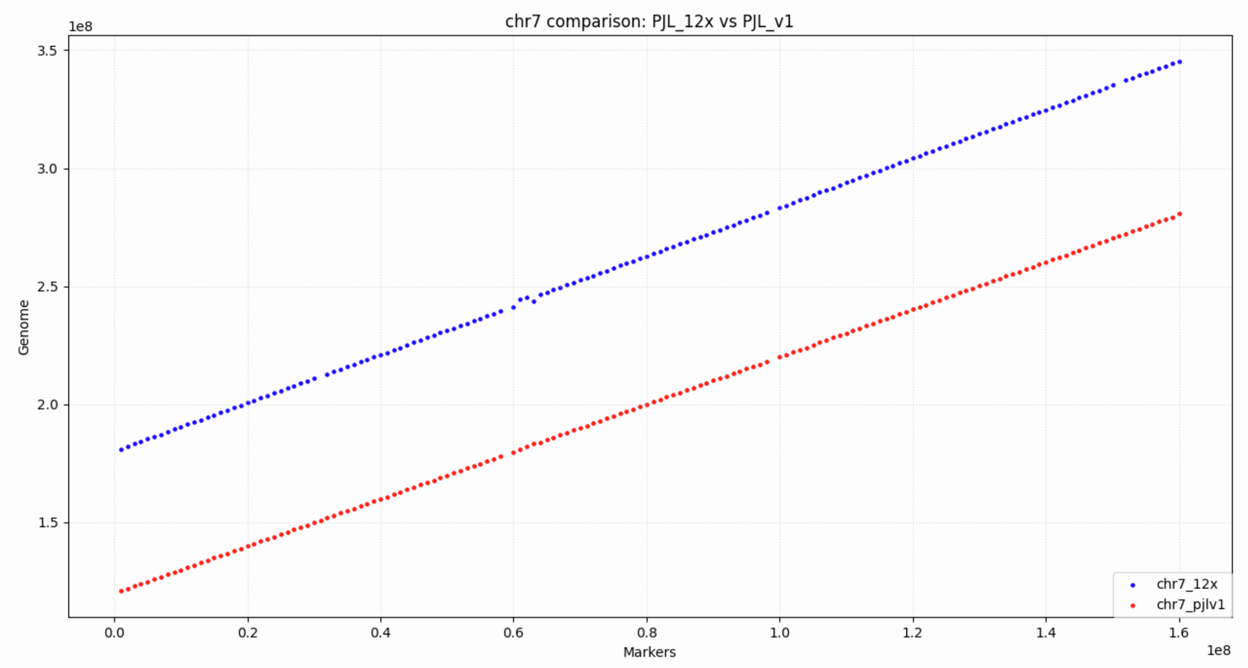
chr7 comparison: PJL_12x vs PJL_v1
1e8
3.5
3.0
2.5
2.0
1.5
Genome
0.0
0.2
0.4
0.6
0.8
1.0
1.2
1.4
1.6
Markers
1e8
chr7_12x
chr7_pjlv1

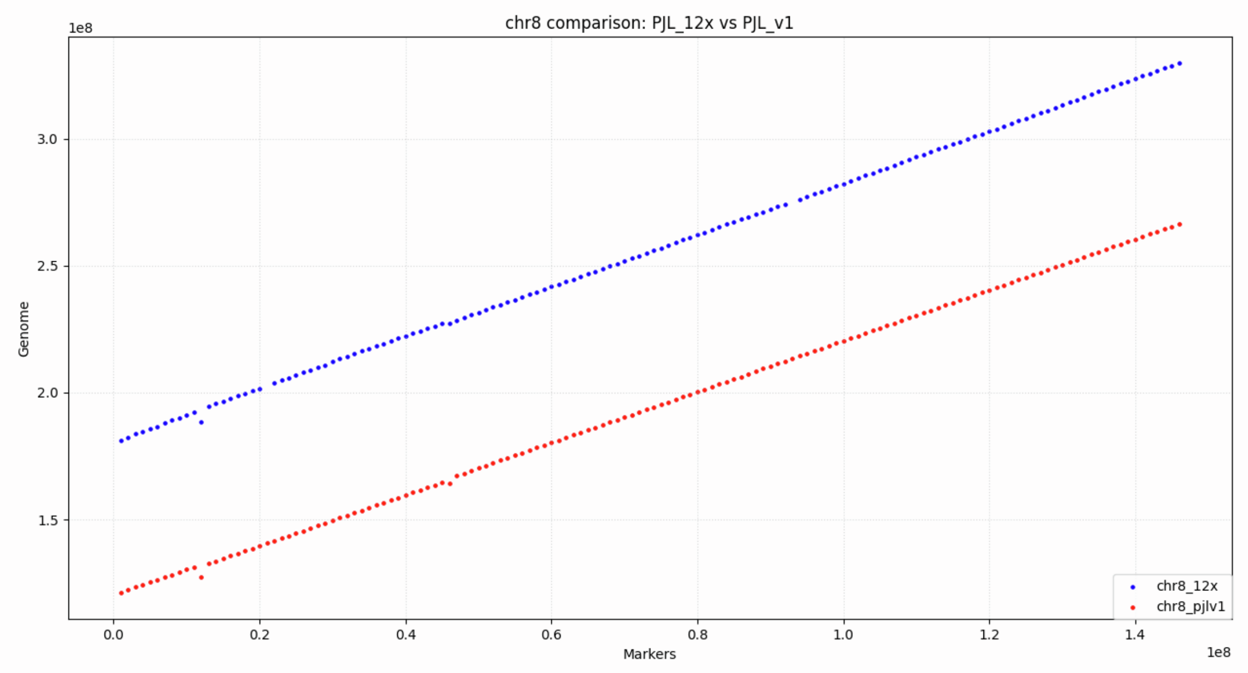
chr8 comparison: PJL_12x vs PJL_v1
1e8
3.0
2.5
2.0
1.5
Genome
0.0
0.2
0.4
0.6
0.8
1.0
1.2
1.4
Markers
1e8
chr8_12x
chr8_pjlv1

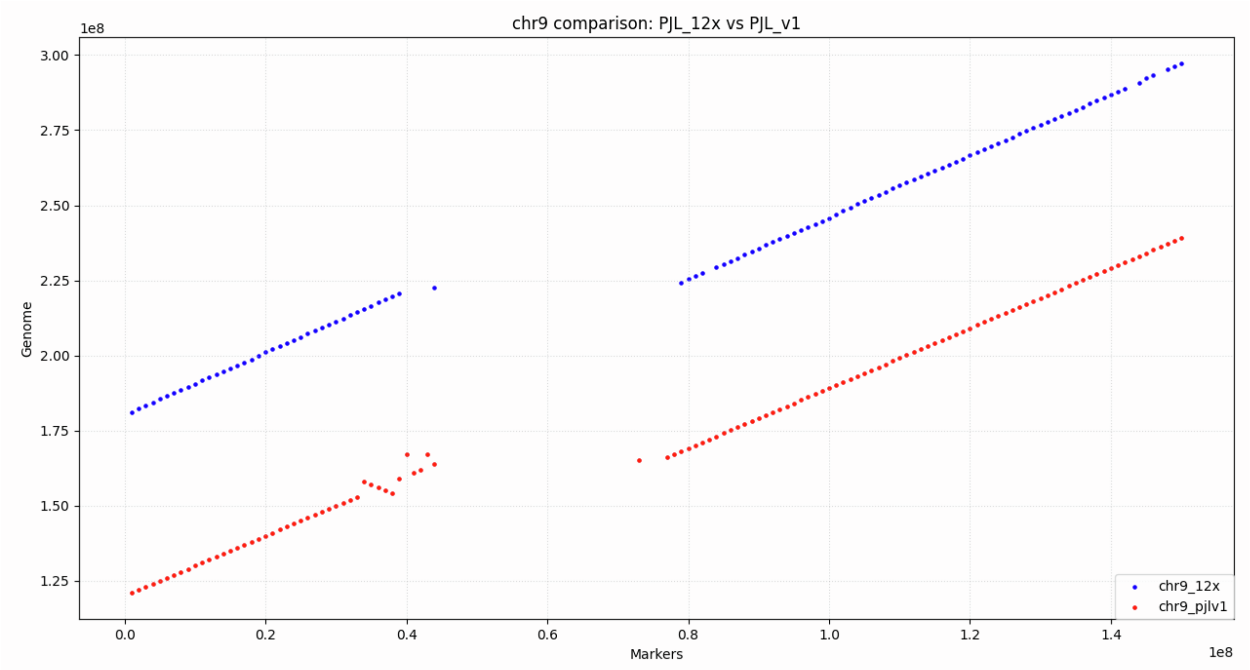
chr9 comparison: PJL_12x vs PJL_v1
1e8
3.00
2.75
2.50
2.25
2.00
1.75
1.50
1.25
Genome
0.0
0.2
0.4
0.6
0.8
1.0
1.2
1.4
Markers
1e8
chr9_12x
chr9_pjlv1

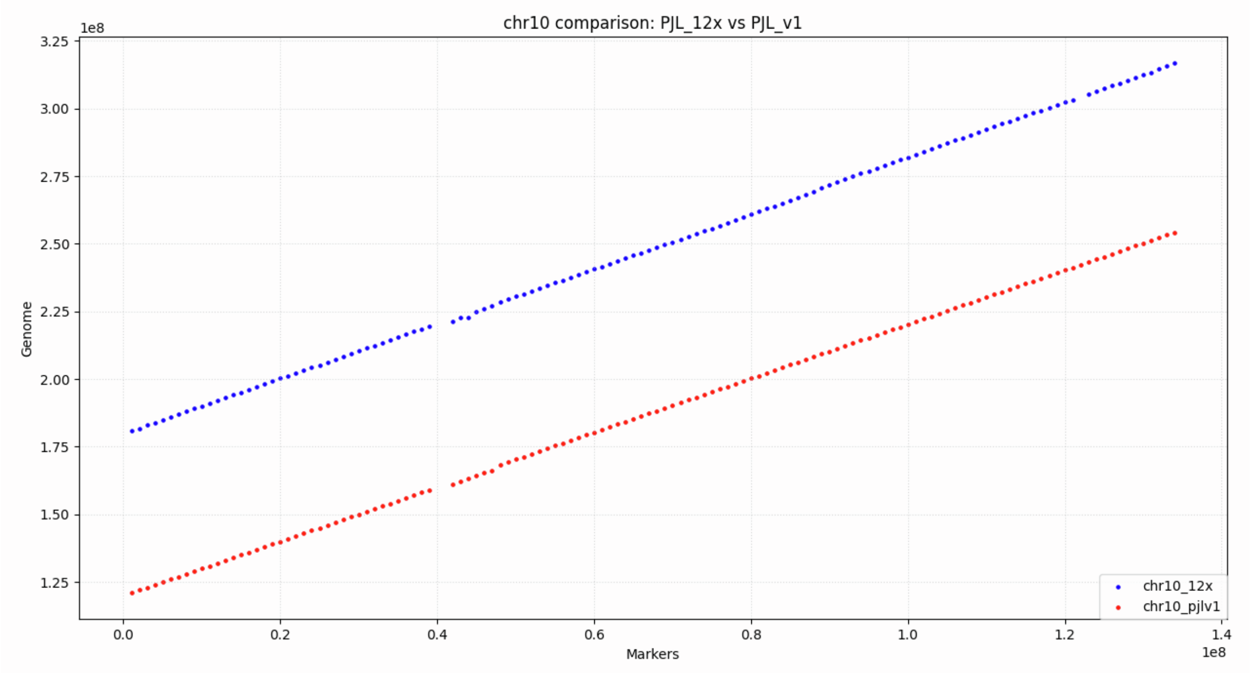
chr10 comparison: PJL_12x vs PJL_v1
1e8
3.25
3.00
2.75
2.50
2.25
2.00
1.75
1.50
1.25
Genome
0.0
0.2
0.4
0.6
0.8
1.0
1.2
1.4
Markers
1e8
chr10_12x
chr10_pjlv1

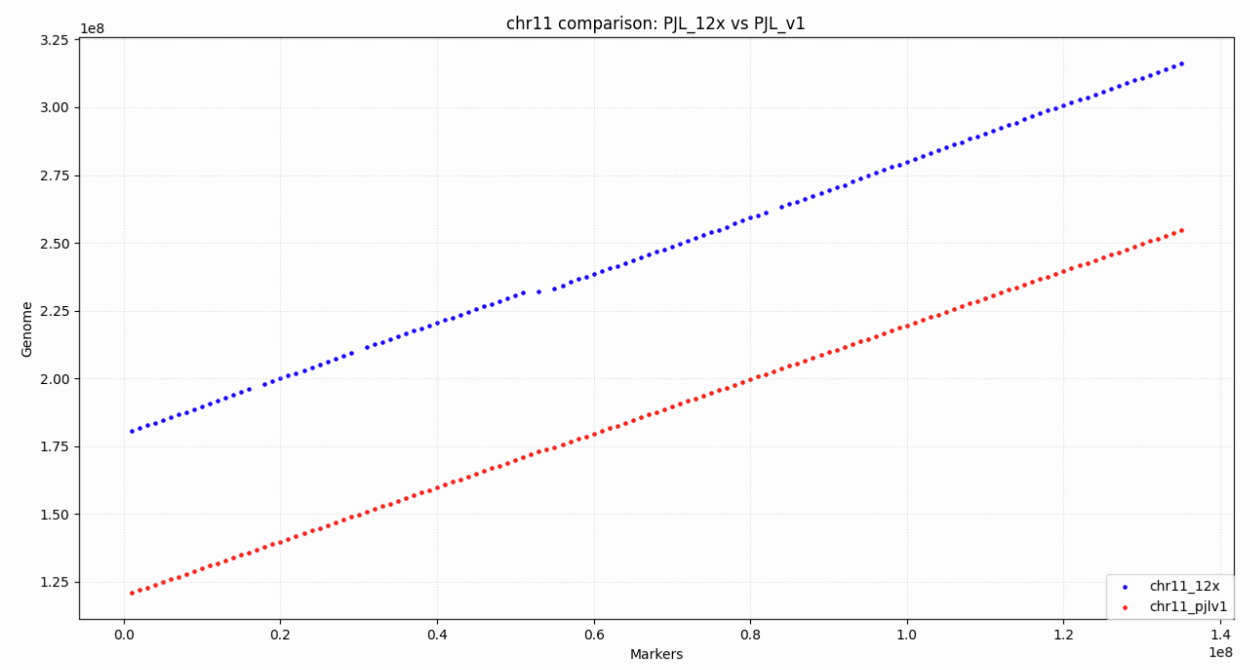
chr11 comparison: PJL_12x vs PJL_v1
1e8
3.25
3.00
2.75
2.50
2.25
2.00
1.75
1.50
1.25
Genome
0.0
0.2
0.4
0.6
0.8
1.0
1.2
1.4
1e8
Markers
chr11_12x
chr11_pjlv1

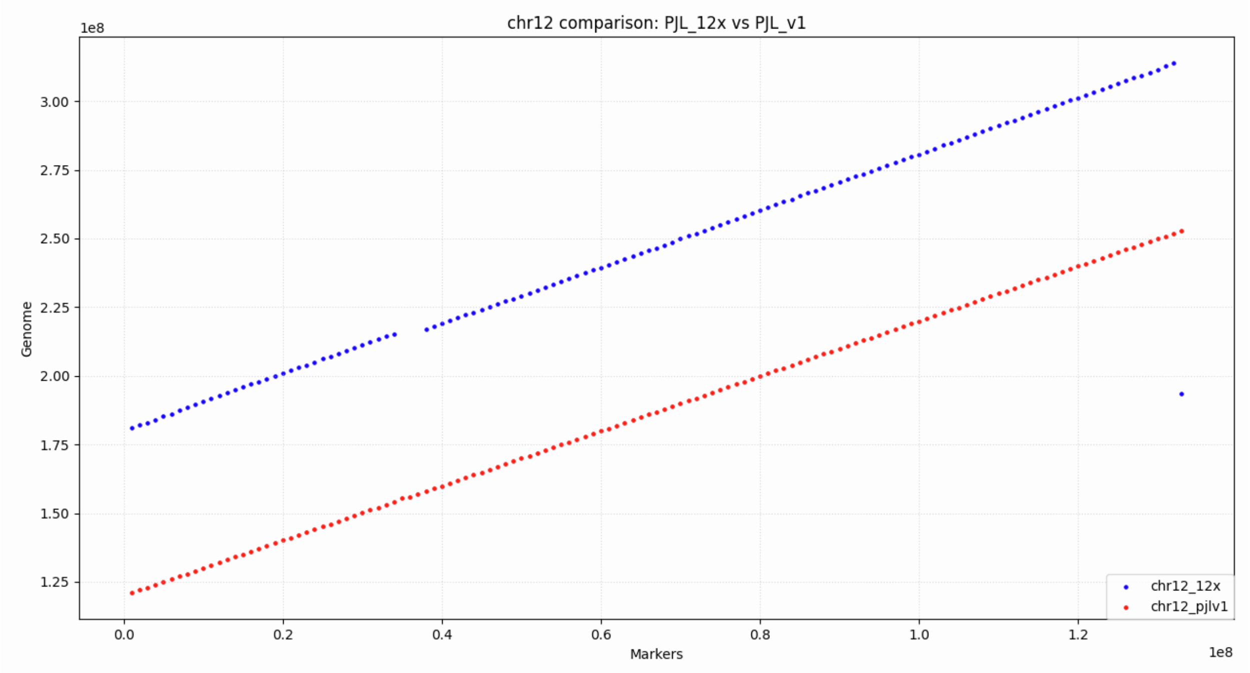
chr12 comparison: PJL_12x vs PJL_v1
1e8
3.00
2.75
2.50
2.25
2.00
1.75
1.50
1.25
Genome
0.0
0.2
0.4
0.6
0.8
1.0
1.2
1e8
Markers
chr12_12x
chr12_pjlv1

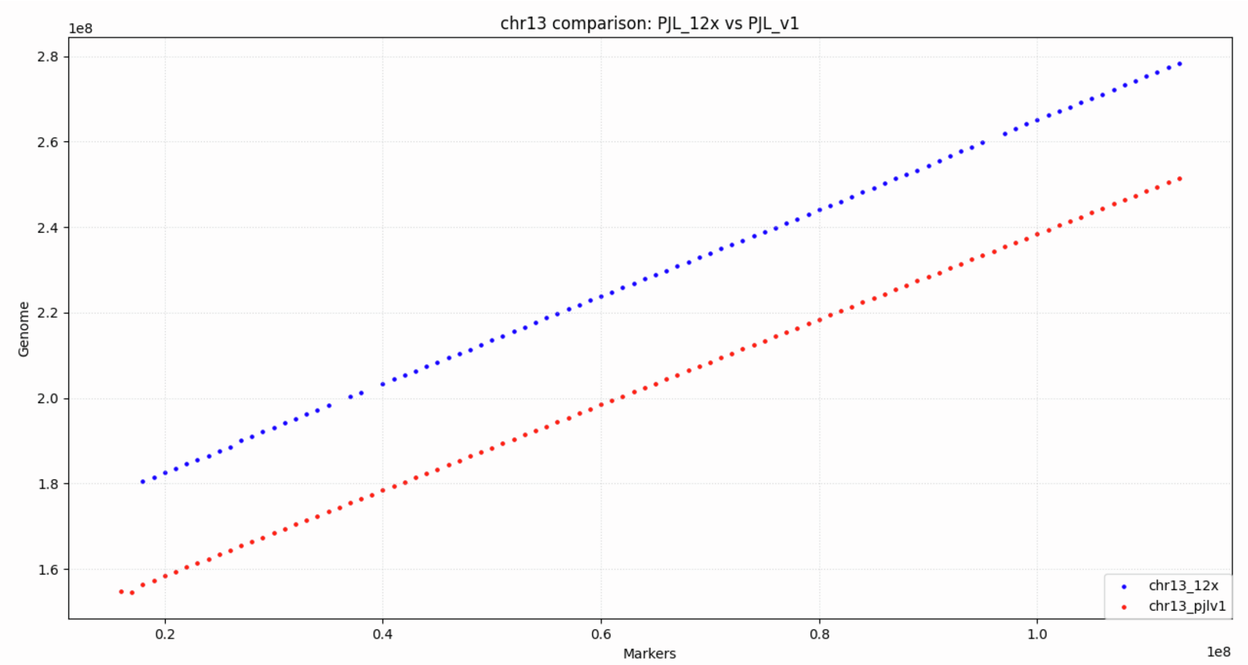
chr13 comparison: PJL_12x vs PJL_v1
1e8
2.8
2.6
2.4
2.2
2.0
1.8
1.6
Genome
0.2
0.4
0.6
0.8
1.0
1e8
Markers
chr13_12x
chr13_pjlv1

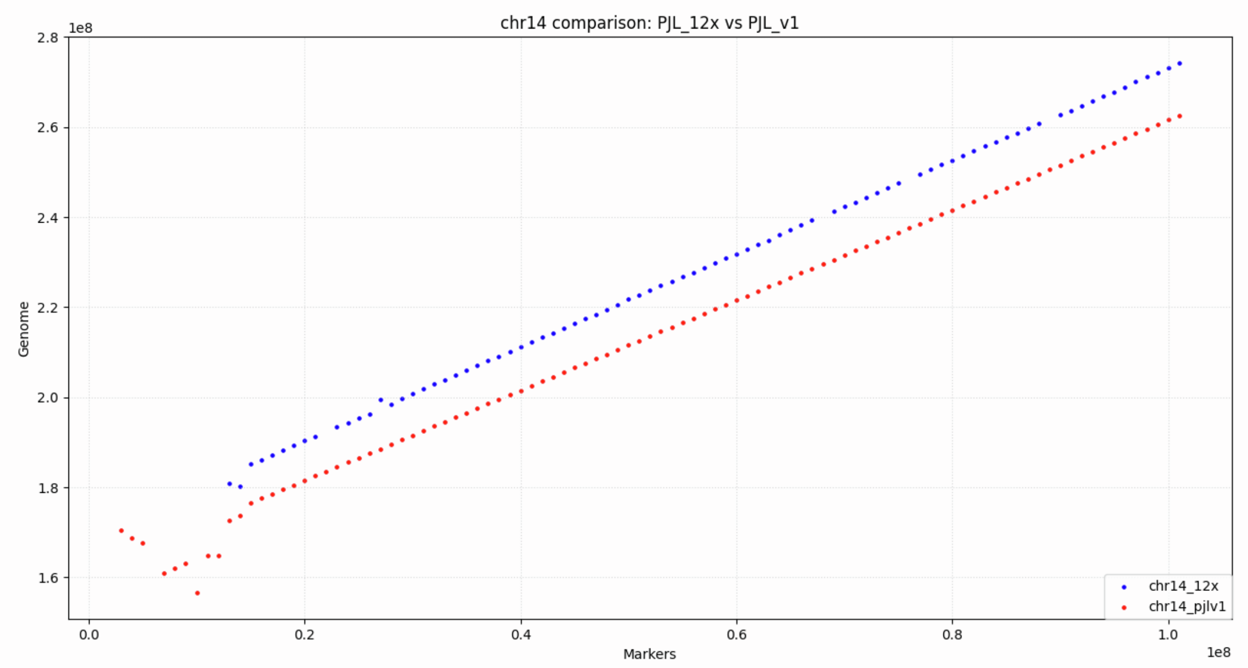
chr14 comparison: PJL_12x vs PJL_v1
1e8
2.8
2.6
2.4
2.2
2.0
1.8
1.6
Genome
0.0
0.2
0.4
0.6
0.8
1.0
1e8
Markers
chr14_12x
chr14_pjlv1

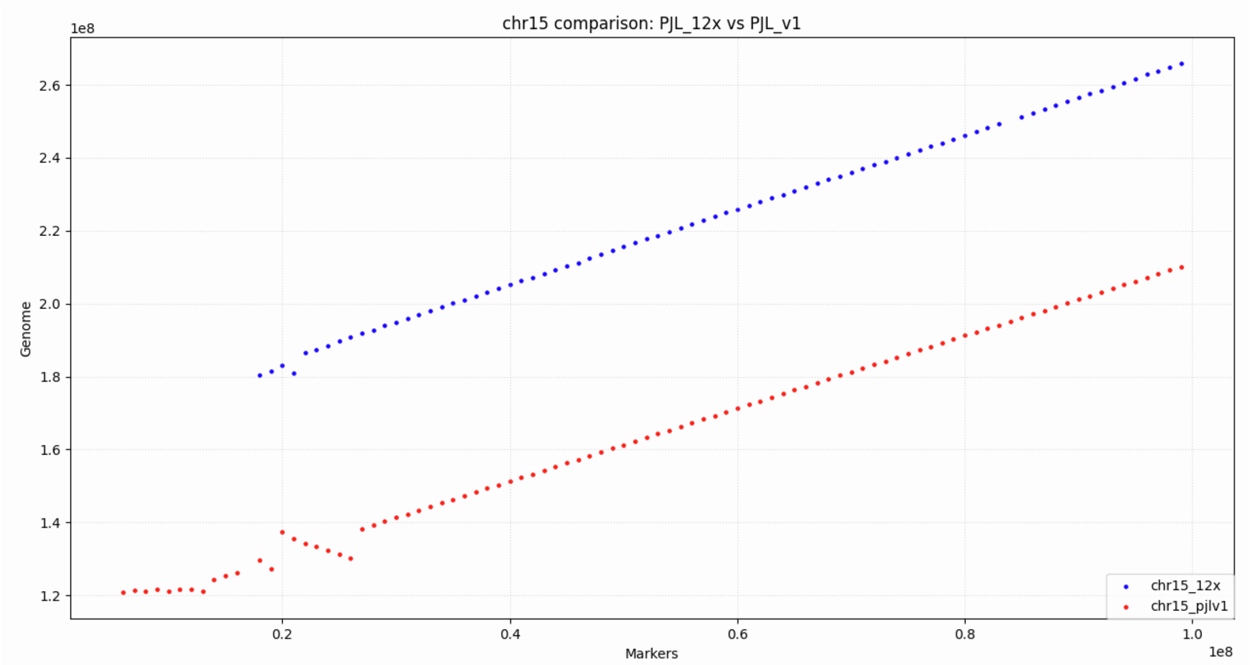
chr15 comparison: PJL_12x vs PJL_v1
1e8
2.6
2.4
2.2
2.0
1.8
1.6
1.4
1.2
Genome
0.2
0.4
0.6
0.8
1.0
1e8
Markers
chr15_12x
chr15_pjlv1

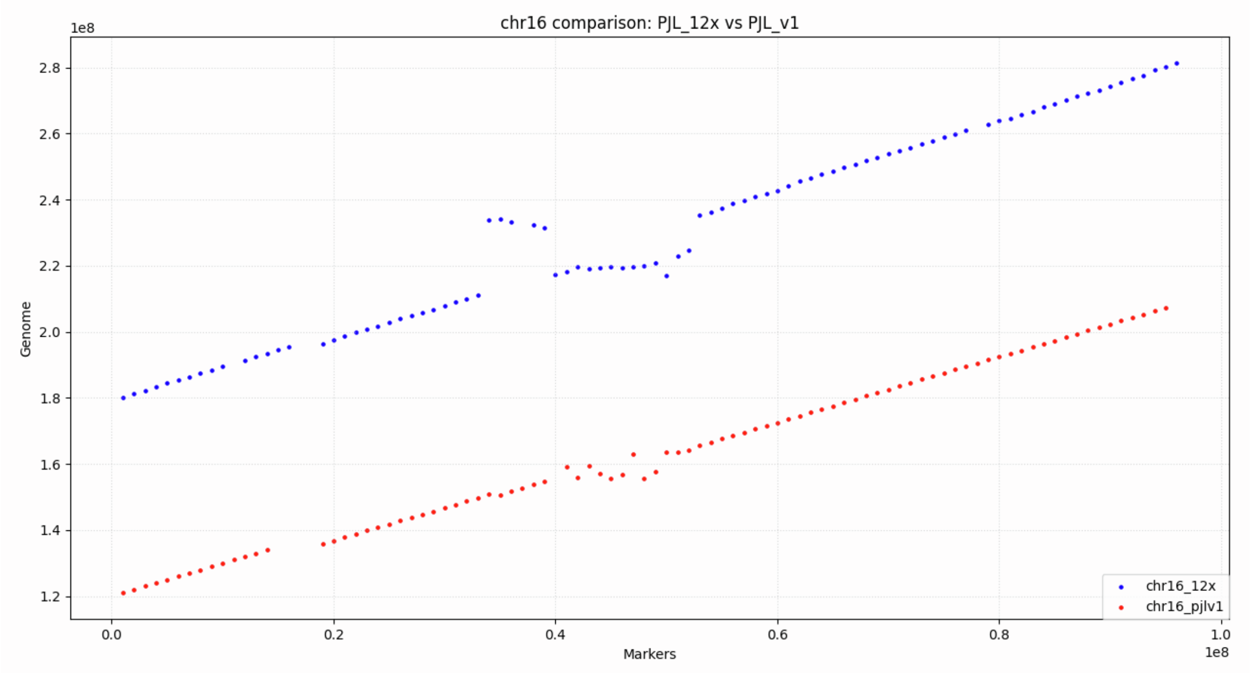
chr16 comparison: PJL_12x vs PJL_v1
1e8
2.8
2.6
2.4
2.2
2.0
1.8
1.6
1.4
1.2
Genome
0.0
0.2
0.4
0.6
0.8
1.0
1e8
Markers
chr16_12x
chr16_pjlv1

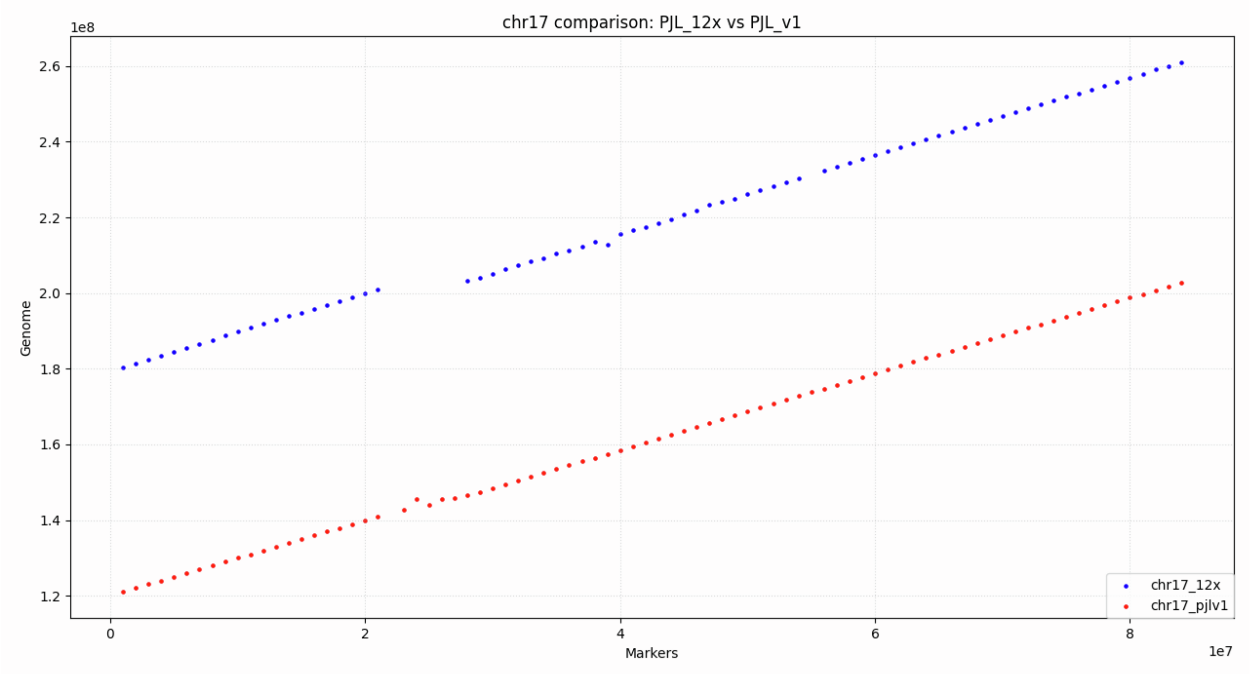
chr17 comparison: PJL_12x vs PJL_v1
1e8
2.6
2.4
2.2
2.0
1.8
1.6
1.4
1.2
Genome
0
2
4
6
8
Markers
1e7
chr17_12x
chr17_pjlv1

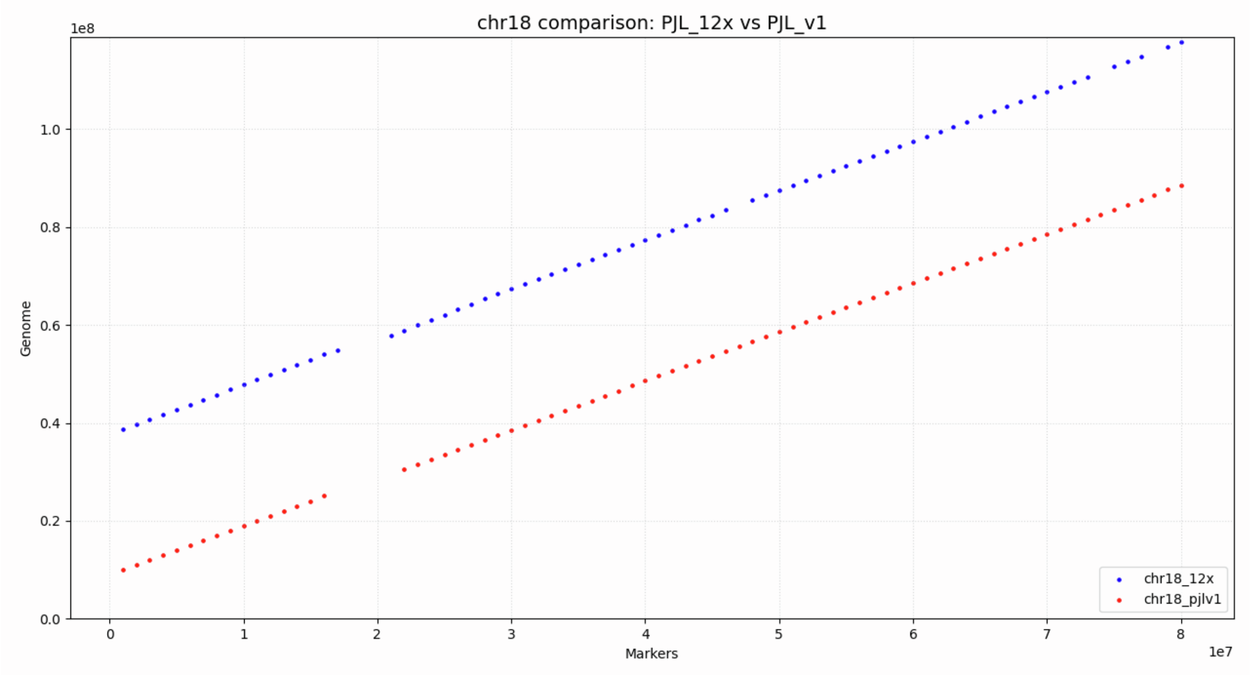
chr18 comparison: PJL_12x vs PJL_v1
1e8
1.0
0.8
0.6
0.4
0.2
0.0
Genome
0
1
2
3
4
5
6
7
8
Markers
1e7
chr18_12x
chr18_pjlv1

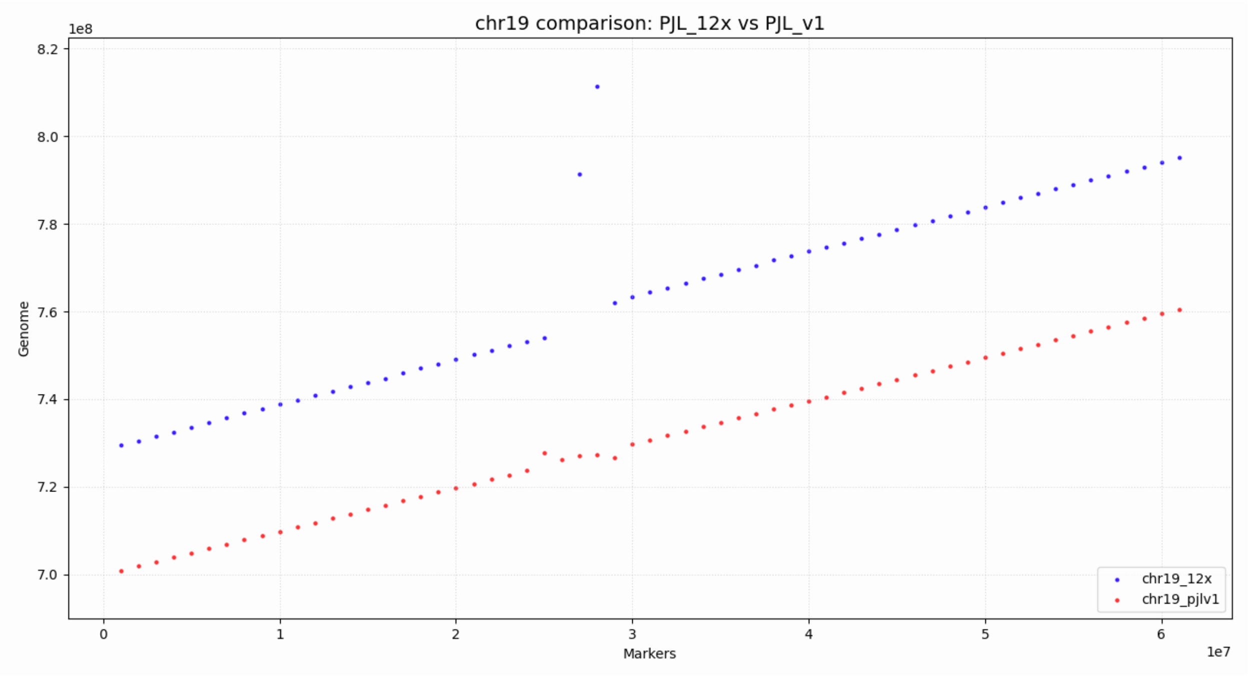
chr19 comparison: PJL_12x vs PJL_v1
1e8
8.2
8.0
7.8
7.6
7.4
7.2
7.0
Genome
0
1
2
3
4
5
6
Markers
1e7
chr19_12x
chr19_pjlv1

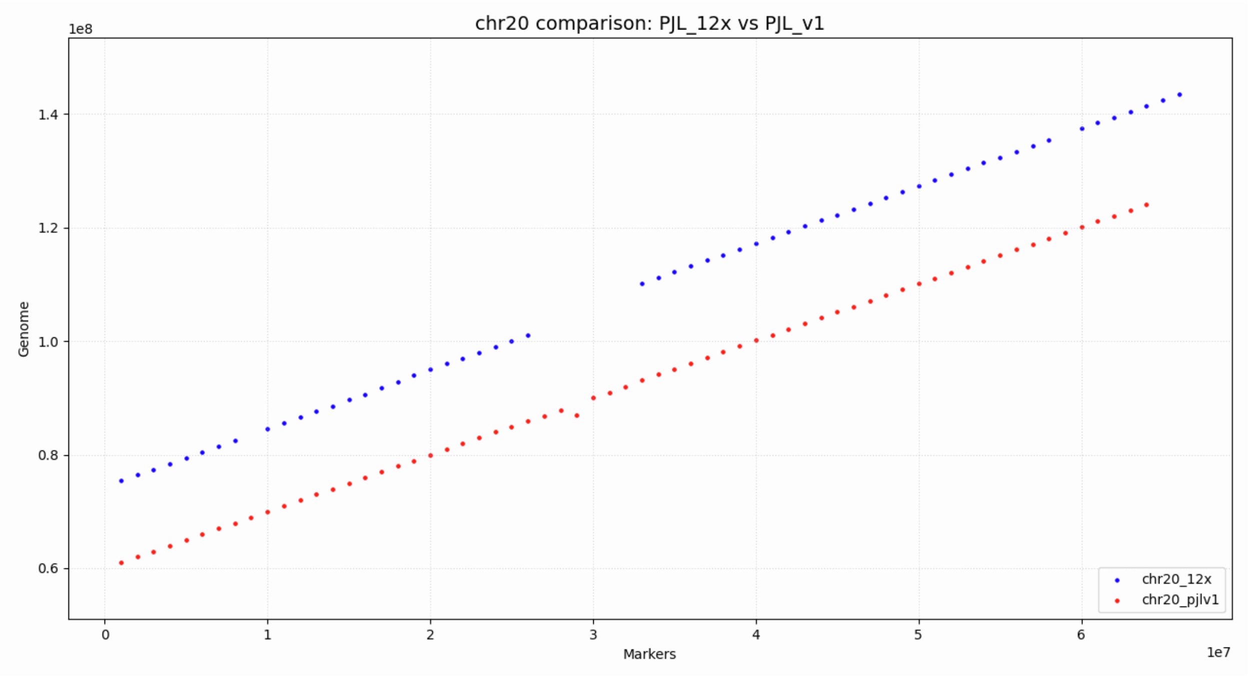
chr20 comparison: PJL_12x vs PJL_v1
1e8
1.4
1.2
1.0
0.8
0.6
Genome
0
1
2
3
4
5
6
Markers
1e7
chr20_12x
chr20_pjlv1

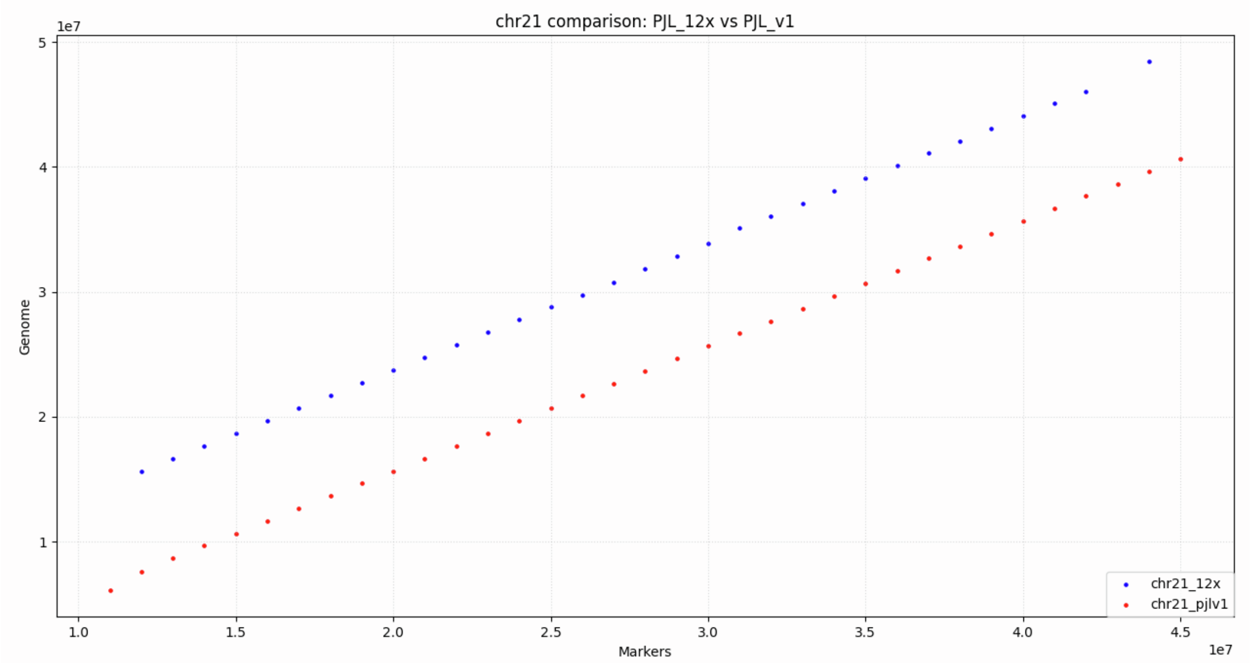
chr21 comparison: PJL_12x vs PJL_v1
1e7
Genome
Markers
1e7
chr21_12x
chr21_pjlv1

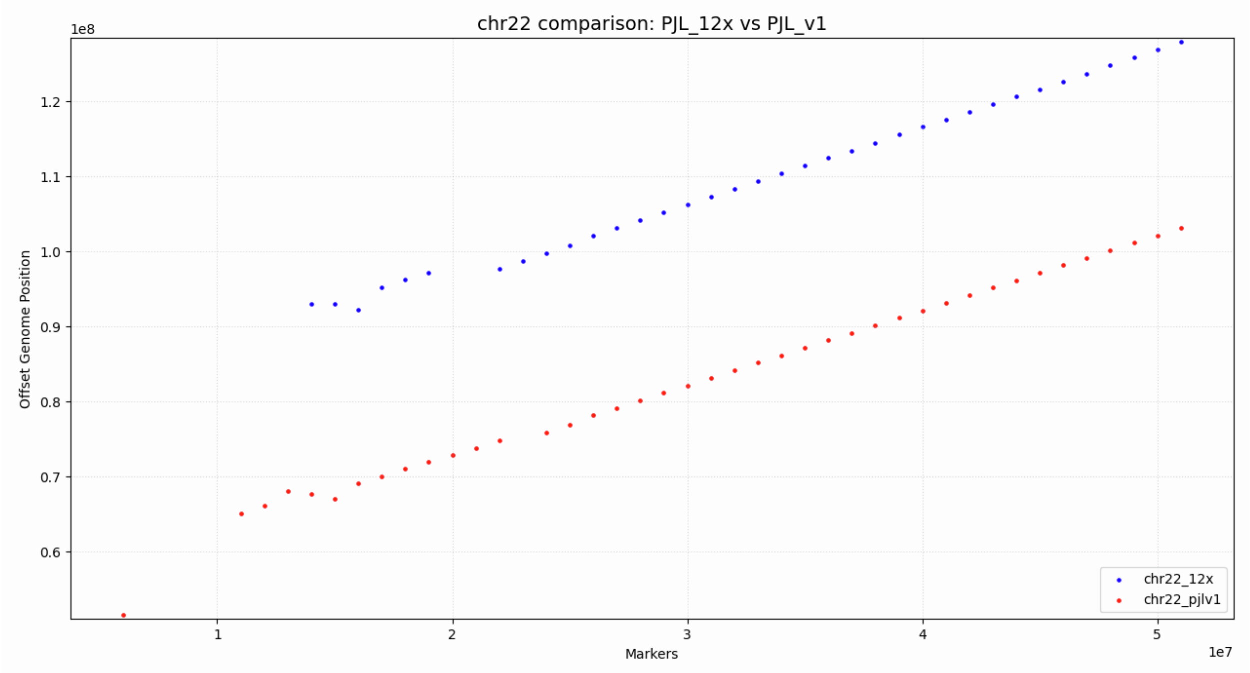
chr22 comparison: PJL_12x vs PJL_v1
1e8
Offset Genome Position
Markers
1e7
chr22_12x
chr22_pjlv1

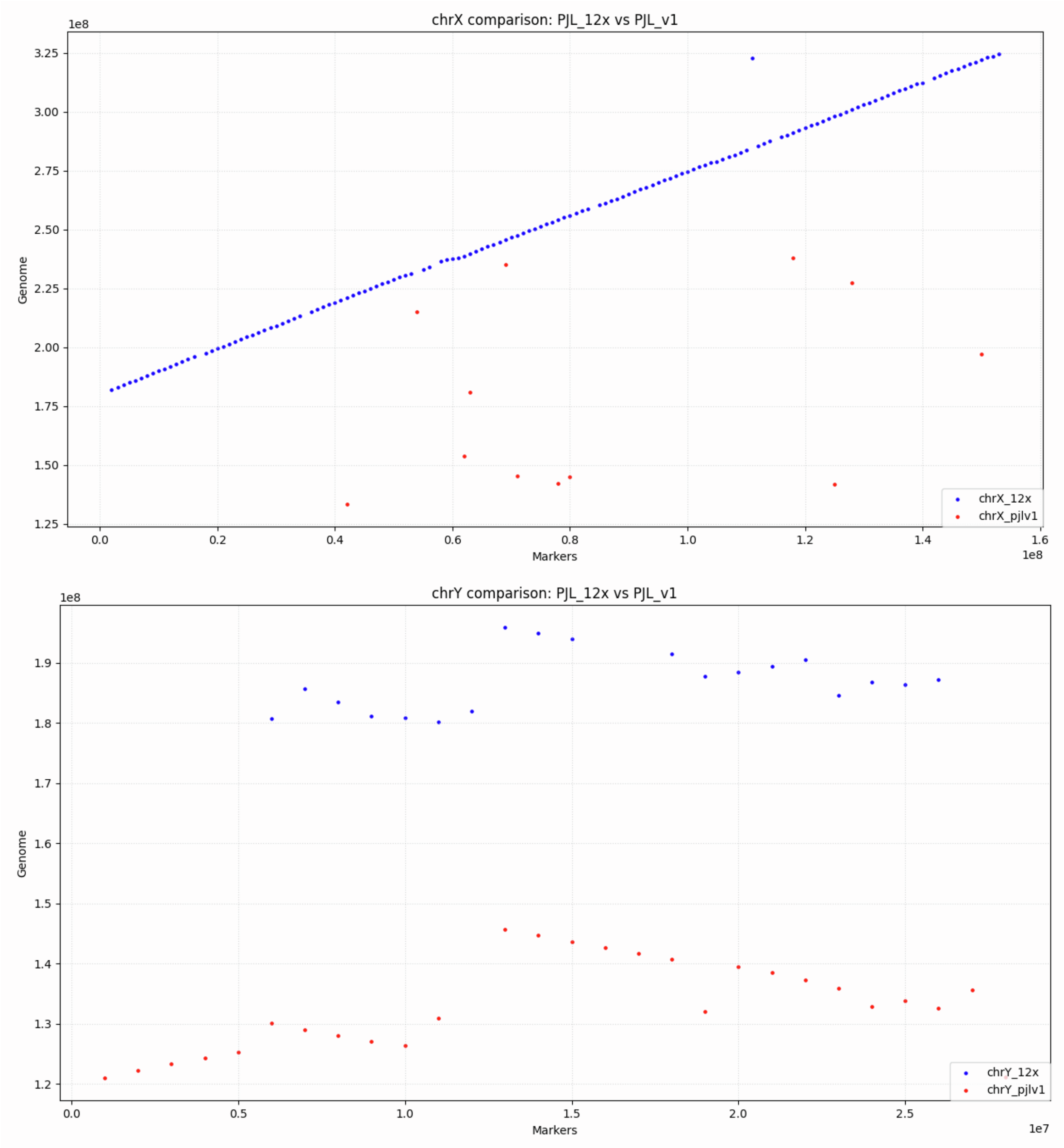

Supplementary Figure S4: Comparison of PJL1.12x against PJL1.v1: Bench marking results for 12x coverage by down-sampled assembly from 30x raw reads of PJL1

Supplementary Table S1:

rsID	Location	Expected Allele	hg38 Allele	Spanish homoINV	T2T Allele	KIn2 Allele	BIB1 Allele	GIH1 Allele	Han1 Allele	ITU1 Allele	PJL_ITU Allele	PJL_Pat Allele	PR1 Allele	Ash1 Allele	GWD1 Allele
rs17627505	chr8:8656549	C>G/C>T	C	C	C	C	C	T	C	C	C	C	C	C	C
rs10503393	chr8:8744686	C>A/C>G	C	G	C	C	C	C	C	C	C	C	C	C	C
rs2428	chr8:8783635	C>A/C>G/C>T	C	T	C	T	C	T	T	C	C	C	C	C	T
rs11774860	chr8:8794369	C>A/C>T	C	C	T	C	C	C	C	C	C	C	C	C	C
rs3827811	chr8:8801528	C>G/C>T	C	G	T	T	C	C	C	T	T	T	C	T	C
rs171547	chr8:8802049	A>G	A	A	A	A	G	A	A	G	G	G	A	G	A
rs1876836	chr8:8824246	T>A/T>C/T>G	T	G	C	C	T	C	C	T	T	T	T	C	C
rs1039916	chr8:8828344	A>C/A>G/A>T	A	G	A	G	A	G	G	A	A	A	A	A	G
rs1178061	chr6: 50946658	C>T	C	T	T	C	C	C	T	C(Hits on chr8)	C(Hits on chr8)	C	T	C	T
rs1178247	not available			C											
rs3885690	chr8:10969359	T>C/T>G	T	A	C	No hits	T	T	T	C		C	C	T	T
rs2409691	chr8:11085766	C>A/C>T/C>G	C	C	T	C	T	C	C	T	T	T	T	T	T
rs13266785	chr8:11132011	G>A	G	G	A	G	A	G	G	G	A	A	A	A	G
rs10282848	chr8:11139157	A>G	A	A	A	A	G	A	A	A	A	A	A	A	A
rs10503417	chr8:11139303	G>A	G	G	A	G	G	G	G	G	A	A	A	A	G
rs2409719	chr8:11159379	A>C/A>G	A	A	G	A	A	A	A	A	G	G	A	A	A

Supplementary Methods:

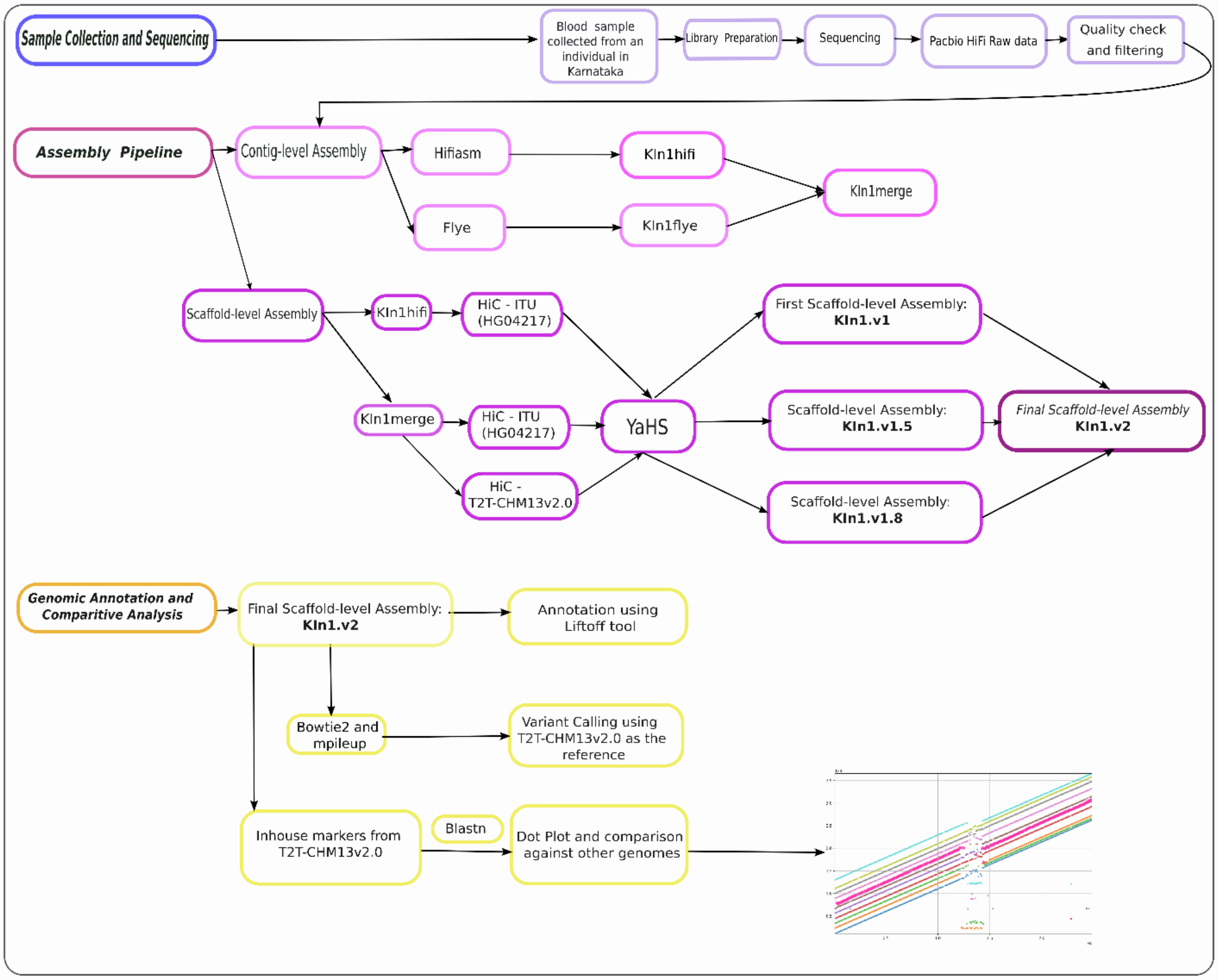

Methodology Flowchart

Commands used in Methods Section:

Liftoff:

- liftofftools variants -r <reference>.fa -t <target.fa> -rg c<reference>.gff3 -tg<target>.gff3
- liftofftools synteny -r <reference>.fa -t <target>.fa -rg<reference>.gff3 -tg<target>.gff3

Seqtk:

- seqtk sample -s100 merged.ccs.fastq.gz N > 12x_merge_ccs.fastq.
